# BDH1-mediated βOHB metabolism ameliorates diabetic kidney disease by activation of NRF2-mediated antioxidative pathway

**DOI:** 10.18632/aging.205248

**Published:** 2023-11-27

**Authors:** Sheng-Rong Wan, Fang-Yuan Teng, Wei Fan, Bu-Tuo Xu, Xin-Yue Li, Xiao-Zhen Tan, Man Guo, Chen-Lin Gao, Chun-Xiang Zhang, Zong-Zhe Jiang, Yong Xu

**Affiliations:** 1 Department of Endocrinology and Metabolism, The Affiliated Hospital of Southwest Medical University, Luzhou, Sichuan 646000, China; 2 Metabolic Vascular Disease Key Laboratory of Sichuan Province, Luzhou, Sichuan 646000, China; 3 Sichuan Clinical Research Center for Nephropathy, Luzhou, Sichuan 646000, China; 4 Department of Orthopaedics, The Affiliated Hospital of Southwest Medical University, Luzhou, Sichuan 646000, China

**Keywords:** βOHB, BDH1, diabetic kidney disease, NRF2, metabolism

## Abstract

A ketogenic diet (KD) and β-hydroxybutyrate (βOHB) have been widely reported as effective therapies for metabolic diseases. β-Hydroxybutyrate dehydrogenase 1 (BDH1) is the rate-limiting enzyme in ketone metabolism. In this study, we examined the BDH1-mediated βOHB metabolic pathway in the pathogenesis of diabetic kidney disease (DKD). We found that BDH1 is downregulated in the kidneys in DKD mouse models, patients with diabetes, and high glucose- or palmitic acid-induced human renal tubular epithelial (HK-2) cells. BDH1 overexpression or βOHB treatment protects HK-2 cells from glucotoxicity and lipotoxicity by inhibiting reactive oxygen species overproduction. Mechanistically, BDH1-mediated βOHB metabolism activates NRF2 by enhancing the metabolic flux of βOHB-acetoacetate-succinate-fumarate. Moreover, *in vivo* studies showed that adeno-associated virus 9-mediated BDH1 renal expression successfully reverses fibrosis, inflammation, and apoptosis in the kidneys of C57 BKS *db/db* mice. Either βOHB supplementation or KD feeding could elevate the renal expression of BDH1 and reverse the progression of DKD. Our results revealed a BDH1-mediated molecular mechanism in the pathogenesis of DKD and identified BDH1 as a potential therapeutic target for DKD.

## INTRODUCTION

Diabetes mellitus is a chronic and serious metabolic disease that has a significant impact on patients and their families all worldwide. The latest report from the International Diabetes Federation predicts that the number of individuals with diabetes worldwide will reach 700 million by 2045 [1]. Diabetic kidney disease (DKD) is the most common microvascular complication of diabetes mellitus and the main cause of chronic kidney disease and end-stage renal disease [2–4]. Thus, studies aimed at elucidating the pathogenesis of DKD and exploring novel therapeutic targets to treat DKD are urgently required.

Under physiological conditions, the production and elimination of reactive oxygen species (ROS) reach a status of dynamic balance or maintain a homeostasis. The abnormal increase in ROS in patients with diabetes leads to oxidative stress injury and inflammatory response and ultimately promotes the occurrence and development of DKD [5–7]. Transcription factor NRF2 is known to maintain intracellular redox homeostasis and reduce cell damage by regulating the expression of antioxidant genes, such as glutamate-cysteine ligase modifier (*Gclm*), glutathione peroxidase 4 (*Gpx4*) and superoxide dismutase 2 (*Sod2*) [8–10]. The protective effect of mitoQ, a mitochondria-targeted antioxidant, on high glucose-treated HK-2 cells is partially blocked by NRF2 knockdown [11]. Fumarate, an intermediate product of the tricarboxylic acid (TCA) cycle, is well known to activate NRF2-mediated antioxidant response [12–14]. However, the role of metabolic regulation in the NRF2-mediated anti-ROS pathway and the pathogenesis of DKD remains undetermined.

In prolonged fasting, strenuous exercise, or disease states, fatty acids produce ketone bodies in the liver through β-oxidation. These ketone bodies are then released into the blood and transported to extrahepatic organs, such as the brain, heart, and kidneys, where they are used as metabolic fuel for the TCA cycle [15]. In the hearts of patients with diabetes, ketone bodies present increased intake and are utilized as an energy source, thereby partially replacing glucose [16]. A ketogenic diet (KD) can elevate endogenous ketone bodies and was initially introduced as a therapeutic method for epilepsy [17]. Moreover, this diet has been reported to be beneficial for a variety of diseases, including diabetic cardiomyopathy and diabetic tractional retinal detachment [18–21]. In addition, KD interventions have been reported to have beneficial effects on type 2 diabetes mellitus (T2DM) and DKD [22–24]. Although increasing evidence supports the relationship between KD and disease, the underlying molecular mechanisms remain unknown.

Ketone bodies are composed of β-hydroxybutyrate (βOHB), acetoacetate (AcAc), and acetone. As the main component of ketone bodies, βOHB has been reported to act as an alternative energy source for the body and mediate signal transduction in metabolic processes, thereby influencing antioxidant production, anti-inflammation, and antiaging [25, 26]. Β-Hydroxybutyrate dehydrogenase 1 (BDH1) is a rate-limiting enzyme of ketone metabolism that can directly catalyze the metabolism of βOHB and promote the reciprocal transformation between βOHB and AcAc [25]. Heart-specific overexpression of *Bdh1* has been reported to significantly ameliorate heart failure by inhibiting oxidative stress [27]. Moreover, upregulation of BDH1-mediated βOHB metabolism increases the concentration of fumarate, which subsequently activates NRF2 to induce the expression of antioxidant stress response elements, thereby inhibiting retinal degeneration under ischemic conditions [28]. However, the role of BDH1-mediated βOHB metabolism in the pathogenesis of DKD is still undetermined.

In this study, we report that reduced BDH1 levels are associated with the pathogenesis of DKD *in vivo* and with glucotoxicity and lipotoxicity *in vitro.* We demonstrate that BDH1 functions as a previously unrecognized activator of NRF2 through the enhancement of βOHB-AcAc-succinate-fumarate metabolic flux. Adeno-associated virus 9 (AAV9)-mediated renal expression of *Bdh1* attenuates the progression of DKD, and either βOHB supplementation or KD feeding could elevate the renal expression of BDH1 and reverse the progression of DKD. In brief, our findings suggest a promising new therapy for DKD via targeting BDH1-mediated βOHB metabolism.

## RESULTS

### BDH1 is downregulated in diabetic kidney and high glucose- or palmitic acid-treated HK-2 cells

**Figure 1:**
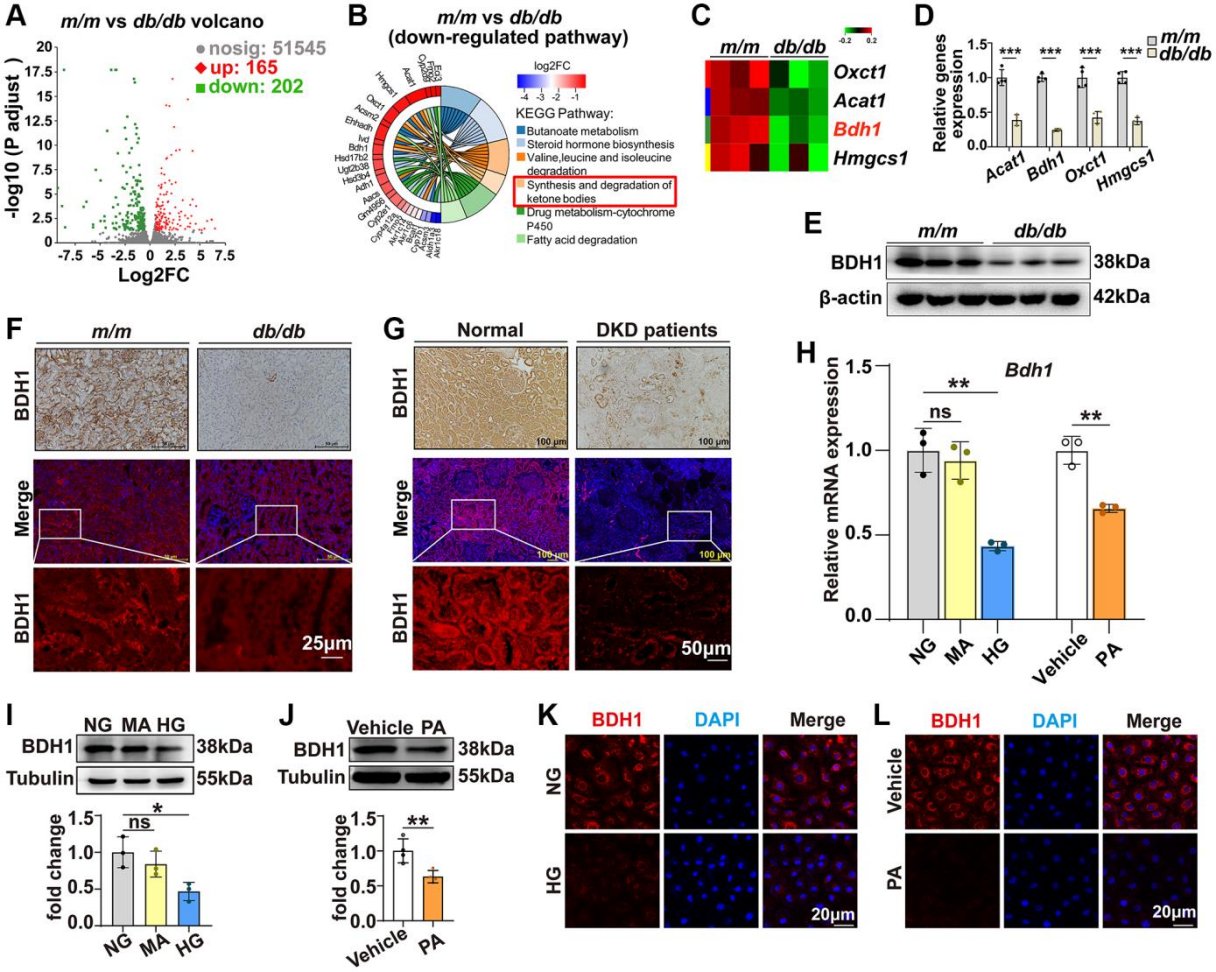
**Expression of BDH1 is downregulated in diabetic kidney and HG- or PA-treated HK-2 cells.** (**A**) Volcano plot showing differentially expressed genes (DEGs) (red, upregulated genes; green, downregulated genes) in the kidneys of *m/m* and *db/db* mice (*n* = 3 mice per group). (**B**) KEGG enrichment analysis showing the top 6 downregulated pathways. (**C**) Four DEGs involved in ketone body synthesis and degradation pathways. (**D**) qRT-PCR analysis showing the mRNA levels of *Acat1*, *Bdh1*, *Oxct1*, and *Hmgcs1* in the kidneys of *m/m* and *db/db* mice (*n* = 3 mice per group). WB (**E**), IHC, and IF (**F**) showing the protein level of BDH1 in the kidneys of *m/m* and *db/db* mice. IHC and IF (**G**) showing the protein level of BDH1 in the kidneys of normal subjects (*n* = 9) and patients with DKD (*n* = 8). (**H**) qRT-PCR showing the mRNA level of *Bdh1* in HK-2 cells treated with HG or PA. Representative WB images showing the protein level of BDH1 in HK-2 cells stimulated with HG (**I**) or PA (**J**). Representative IF images showing the protein level of BDH1 in HK-2 cells stimulated with HG (**K**) or PA (**L**). All results are representative of three independent experiments. Values are presented as mean ± standard deviation. Bar: 50 and 25 μm in **F**, and 100 and 50 μm in **G**, 20 μm in **K** and **L**. Abbreviations: BDH1: β-hydroxybutyrate dehydrogenase 1; NG: normal glucose; HG: high glucose; PA: palmitic acid; MA: mannitol; DEGs: differentially expressed genes; WB: western blot; IHC: immunohistochemistry; IF: immunofluorescence; DKD: diabetic kidney disease. ^*^P < 0.05; ^**^P < 0.01; ^***^P < 0.001; Abbreviation: ns: no significance.

DKD represents a major microvascular complication in patients with diabetes and is the leading cause of chronic kidney disease and end-stage renal disease [2–4]. To gain a comprehensive understanding of potential DKD regulators, we performed RNA-seq analysis by comparing the gene expression in the kidneys of *db/db* and *m/m* mice at the age of 20 weeks when *db/db* mice exhibited an apparent increase in the urinary albumin-to-creatinine ratio (ACR) (Supplementary Figure 1). The volcano plot in Figure 1A shows that 165 upregulated differentially expressed genes (DEGs) and 202 downregulated DEGs were identified in the kidneys of *db/db* mice (detailed information on DEGs is provided in the Supplementary Table 1). Kyoto Encyclopedia of Genes and Genomes (KEGG) analysis shows that the “synthesis and degradation of ketone bodies” pathway is significantly downregulated (Figure 1B, 1C). Given that KD has been reported as an effective treatment for diabetes [29, 30], we presumed that the downregulation of the synthesis and degradation of ketone body pathways participates in the pathogenesis of DKD. Consistently, qRT-PCR analysis confirmed that the expression of *Bdh1*, *Oxct1*, *Acat1*, and *Hmgcs1* was altered (Figure 1D). Among these four pathway members, *Bdh1* has been reported to protect the heart from heart failure in a transverse aortic constriction mouse model [27]. Thus, to identify whether *Bdh1* is involved in the pathogenesis of DKD, we further identified the protein level of BDH1. As shown in Figure 1E, the protein level of BDH1 in the kidneys of *db/db* mice is significantly lower than that in the kidneys of *m/m* mice. Moreover, the decrease in BDH1 expression was confirmed using immunohistochemistry and immunofluorescence (IF) analysis (Figure 1F). Consistent with the DKD mouse model, we observed the downregulation of BDH1 in the renal tissues of diabetic patients with kidney disease using immunohistochemistry and IF staining (Figure 1G). The characteristics of the control subjects and DKD patients are summarized in Supplementary Table 2.

Hyperglycemia and hyperlipidemia are the two most evident characteristics of type 2 diabetes [31]. Therefore, we established high glucose (HG)-induced glucotoxicity and palmitic acid (PA)-induced lipotoxicity cell models using HK-2 cells to evaluate the effects of HG or PA on BDH1 expression. As expected, both the mRNA and protein levels of BDH1 were reduced by the HG and PA treatments in HK-2 cells (Figure 1H–1J), which was confirmed using IF analysis (Figure 1K, 1L). These results indicated that decreased BDH1 expression is related to the pathogenesis of DKD.

Next, we investigated the mechanism underlying the decreased BDH1 expression in DKD mice and HG- or PA-treated HK-2 cells. Given that the mRNA expression of *BDH1* could be transcriptionally regulated by *MTA2*-triggered R-loop in hepatocellular carcinoma stem cells [32], we hypothesized that HG- and PA-induced *BDH1* reduction might be mediated by *MTA2*. To confirm this, we measured *MTA2* expression levels in a diabetic mouse model and in glucotoxicity and lipotoxicity cell models. We identified that *MTA2* expression was up-regulated in the kidneys of *db/db* mice (Supplementary Figure 2A) and HG- (Supplementary Figure 2B) and PA-treated HK-2 cells (Supplementary Figure 2C), indicating that the reduction in *BDH1* in DKD mouse models or HK-2 cells treated with HG or PA may be related to *MTA2* reduction.

### AAV9-mediated BDH1 renal expression alleviates the progression of DKD

**Figure 2:**
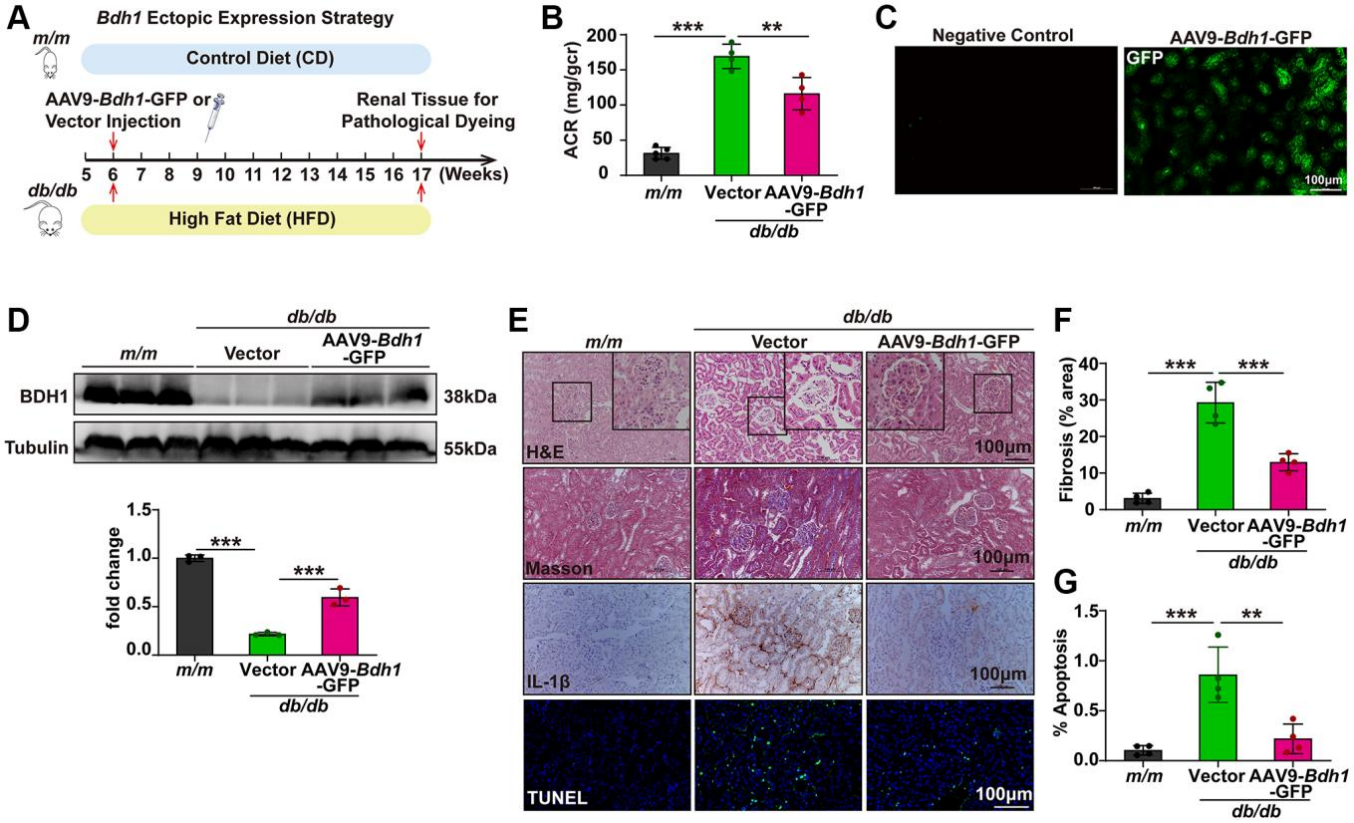
**AAV9-mediated BDH1 ectopic expression in kidneys alleviates the progression of DKD.** (**A**) Schematic diagram illustrating the animal experimental design. During the experiment, *m/m* mice were fed a control diet (CD), while *db/db* mice were fed an HFD. Six-week-old male *db/db* mice were randomized to receive tail vein injection of AAV9-*Bdh1*-GFP (3.40E+12 vg/mL) or vector (1.90E+13 vg/mL). Mice were euthanized at 11 weeks after AAV9 injection. (**B**) Urinary ACR values of mice in the indicated groups (*n* = 5 in *m/m* group, *n* = 4 in vector, and AAV-*Bdh1*-GFP injected *db/db* group). (**C**) Representative renal fluorescent images of the mice 11 weeks after caudal vein delivery of AAV-*Bdh1*-GFP revealing GFP expression in the kidneys. (**D**) Representative WB image showing the protein level of BDH1 in the kidneys of indicated groups. (**E**) Representative photomicrographs of H&E, Masson, IHC (IL-1β), and TUNEL staining showing the pathological changes in the kidneys of indicated groups. (**F**) Quantification of the fibrosis area in the kidneys of indicated groups (*n* = 4 per group). (**G**) Quantification of apoptosis-positive cells in the kidneys of indicated groups (*n* = 4 per group). All results are representative of three independent experiments. Values are presented as mean ± standard deviation. Bar: 100 μm in **C** and **E**. Abbreviations: CD: control diet; HFD: high fat diet; ACR: albumin-to-creatinine ratio; AAV9: adeno-associated virus 9; BDH1: β-hydroxybutyrate dehydrogenase 1; DKD: diabetic kidney disease; WB: western blot; H&E: hematoxylin and eosin; IHC: immunohistochemistry. ^*^P < 0.05; ^**^P < 0.01; ^***^P < 0.001.

Based on the pronounced ability of BDH1 to inhibit ROS overproduction and inflammation, we explored the therapeutic efficacy of BDH1 in *db/db* mice. AAV9 is a gene delivery system that is relatively specific for renal expression (Supplementary Figure 3A), therefore, we used AAV9 as a gene delivery tool for renal BDH1 expression. The experimental strategy is illustrated in Figure 2A. Compared with vector-injected *db/db* mice, AAV9-*Bdh1*-GFP-injected *db/db* mice showed increased blood levels of βOHB (Supplementary Figure 3B) and unchanged plasma levels of total cholesterol (Supplementary Figure 3C) and total triglycerides (Supplementary Figure 3D). Eleven weeks after injection of the control or *Bdh1*-encoding virus, we performed an assay of ACR, which is the most important indicator of kidney function. Although the BDH1 renal expression did not affect the body weight, fasting blood glucose (Supplementary Figure 4A, 4B), and kidney-to-body weight ratio (Supplementary Table 3), we observed a significantly lower ACR in AAV9-*Bdh1*-GFP-injected mice than that in vector-injected mice (Figure 2B). To confirm whether *Bdh1* was effectively expressed in the kidneys of mice based on the AAV9 vector, we measured the fluorescence intensity of GFP, which was co-expressed with *Bdh1*. AAV9-encoding *Bdh1* was successfully delivered to the kidneys 11 weeks after injection (Figure 2C). As expected, we observed increased BDH1 expression in the kidneys of AAV9-*Bdh1*-GFP-injected mice compared with vector-injected mice (Figure 2D). In further histological analyses, AAV9-*Bdh1*-GFP-injected *db/db* mice showed a normal glomerular morphology while vector-injected mice presented glomerular hypertrophy (Figure 2E). In addition, DKD pathology-related fibrosis, inflammation, and apoptosis were substantially reduced by AAV9-*Bdh1*-GFP injection (Figure 2E–2G). Collectively, these findings strongly support the promising application of BDH1 as a therapeutic target for DKD.

### BDH1-mediated βOHB metabolism reverses HG- or PA-induced ROS overproduction and inflammation

**Figure 3:**
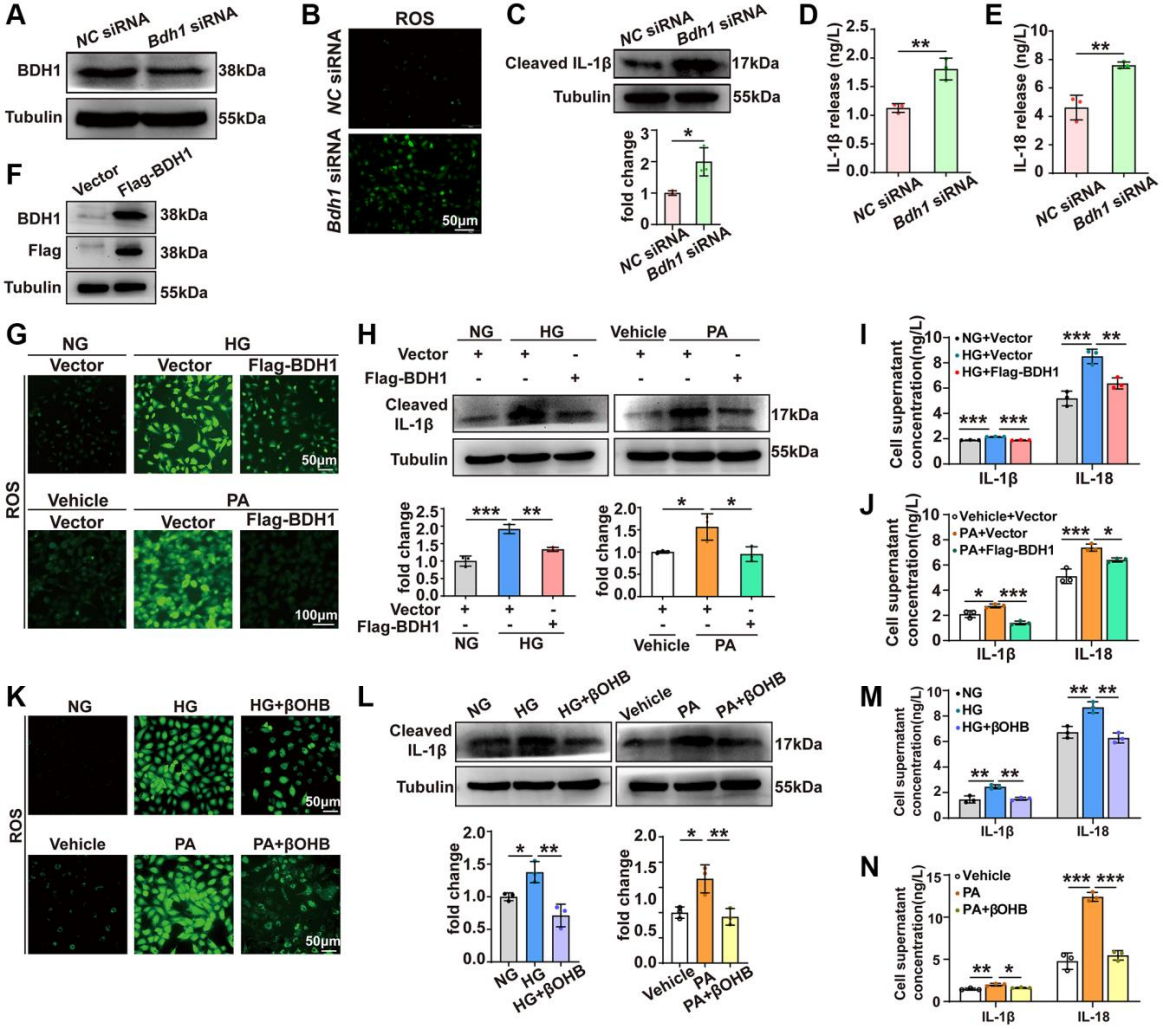
**BDH1-mediated βOHB metabolism reverses HG or PA-induced ROS overproduction and inflammation.** (**A**) Representative WB image showing the protein level of BDH1 in HK-2 cells transfected with *NC* siRNA or *Bdh1* siRNA. (**B**) DCFH-DA probe was used to identify the level of ROS in HK-2 cells transfected with *NC* siRNA or *Bdh1* siRNA. (**C**) Representative WB image showing the protein level of IL-1β in HK-2 cells transfected with *NC* siRNA or *Bdh1* siRNA. (**D**, **E**) ELISA showing the levels of IL-1β (**D**) and IL-18 (**E**) in the cell culture supernatant of HK-2 cells transfected with *NC* siRNA or *Bdh1* siRNA. (**F**) Representative WB image showing the protein level of BDH1 in HK-2 cells transfected with vector or Flag-BDH1. (**G**) DCFH-DA probe was used to identify the level of ROS in HK-2 cells based on the indicated treatment. (**H**) Representative WB image showing the protein level of IL-1β in HK-2 cells based on the indicated treatment. (**I**, **J**) ELISA showing the levels of IL-1β and IL-18 in the cell culture supernatant of BDH1-overexpressed HK-2 cells treated with HG (**I**) or PA (**J**). For samples used in **G**-**J**, HK-2 cells were transfected with the plasmids overexpressing Flag-BDH1 or vector for 6 h and then treated with HG or PA for 48 h. (**K**) DCFH-DA probe was used to identify ROS levels in HK-2 cells based on the indicated treatment. (**L**) Representative WB image showing the protein level of IL-1β in HK-2 cells based on the indicated treatment. (**M**, **N**) ELISA showing the levels of IL-1β and IL-18 in the cell culture supernatant of βOHB-supplemented HK-2 cells treated with HG (**M**) or PA (**N**). For samples used in **K**-**N**, HK-2 cells were treated with vehicle or βOHB and HG or PA for 48 h. All results are representative of three independent experiments. Values are presented as mean ± standard deviation. Bar: 50 μm in **B**, 50 μm in top panels and 100 μm in bottom panels in **G**, 50 μm in **K**. Abbreviations: BDH1: β-hydroxybutyrate dehydrogenase 1; βOHB: β-hydroxybutyrate; HG: high glucose; PA: palmitic acid; ROS: reactive oxygen species; WB: western blot. ^*^P < 0.05; ^**^P < 0.01; ^***^P < 0.001.

To examine whether BDH1 downregulation contributes to HG- or PA-induced cell injury, we knocked down BDH1 in HK-2 cells (Figure 3A). Given that increased ROS plays a central role in the pathogenesis of diabetic microvascular complications including DKD [33], and that ROS overproduction is related to inflammation [34], we next identified the ROS levels and observed a significant increase in ROS in HK-2 cells transfected with *Bdh1* siRNA (Figure 3B). In addition, BDH1 knockdown elevated the protein level of the activated pro-inflammatory factor cleaved IL-1β (Figure 3C) and secretory IL-1β and IL-18 (Figure 3D, 3E). Collectively, these results suggest that BDH1 deficiency mediates HG- or PA-induced cell injury through the loss of anti-ROS function.

Since BDH1 deficiency led to increased ROS production and inflammation, we next attempted to determine whether HG- or PA-induced BDH1 reduction mediates HG- or PA-induced cell injury. Thus, we transfected HK-2 cells with a Flag-BDH1 overexpression plasmid to block HG- or PA-induced BDH1 reduction (Figure 3F). ROS assay showed that BDH1 overexpression significantly reduced HG-induced ROS overproduction (Figure 3G, top panels). In PA-treated cells, BDH1 overexpression nearly completely reversed PA-induced ROS overproduction (Figure 3G, bottom panels). Regarding inflammation, BDH1 overexpression reversed the HG- or PA-induced activation of IL-1β (Figure 3H) and the upregulation of secretory IL-1β and IL-18 (Figure 3I, 3J). This suggests that BDH1 may play a protective role in DKD pathogenesis and that pathological hyperglycemia- and hyperlipidemia-induced BDH1 reduction might mediate cell injury.

Given that BDH1 is a key enzyme that catalyzes the first step of βOHB metabolism, we next attempted to determine whether βOHB supplementation could exert a protective effect on HG- or PA-treated HK-2 cells. Consistent with the results for BDH1 overexpression, βOHB supplementation markedly reversed HG- or PA-induced ROS overproduction (Figure 3K). Similarly, βOHB supplementation reversed the HG- or PA-induced activation of IL-1β (Figure 3L) and upregulation of secretory IL-1β and IL-18 (Figure 3M, 3N). In generally, these findings suggest that BDH1-mediated βOHB metabolism plays an important role in protection against HG- or PA-induced cell injury.

### BDH1-mediated βOHB metabolism promotes NRF2 nuclear translocation

**Figure 4:**
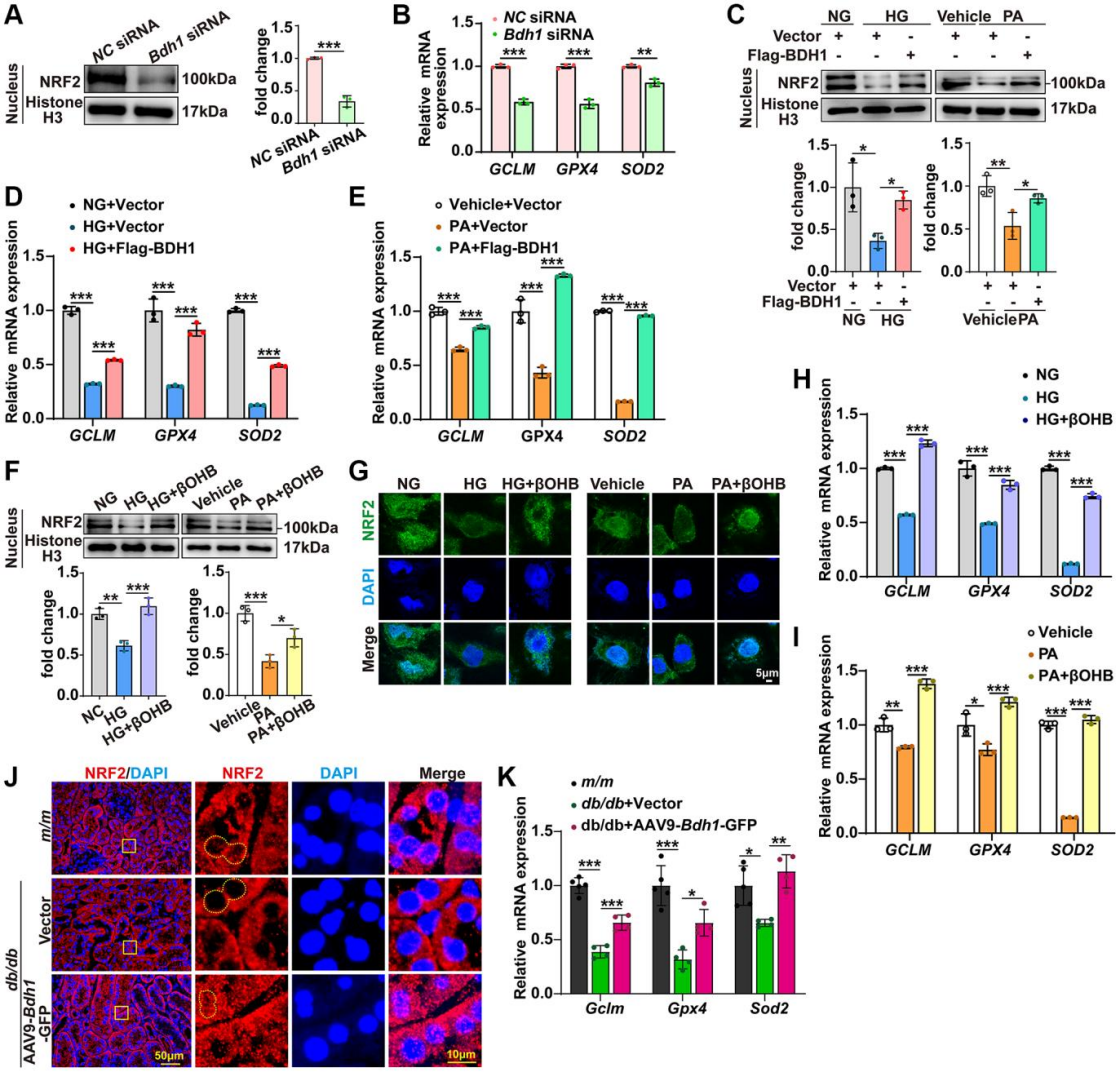
**BDH1-mediated βOHB metabolism promotes NRF2 nuclear translocation.** (**A**) Representative WB image showing the protein level of NRF2 protein in the nuclear translocation of HK-2 cells transfected with *NC* siRNA or *Bdh1* siRNA. (**B**) mRNA expression of *GCLM*, *GPX4*, and *SOD2* in HK-2 cells transfected with *NC* siRNA or *Bdh1* siRNA. (**C**) Representative WB images showing the protein level of NRF2 in the nuclear translocation of BDH1-overexpressed HK-2 cells treated with HG or PA. (**D**, **E**) mRNA expression of *GCLM*, *GPX4*, and *SOD2* in BDH1-overexpressed HK-2 cells stimulated with HG (**D**) or PA (**E**). (**F**) Representative WB images showing the protein level of NRF2 in the nuclear translocation of βOHB-supplemented HK-2 cells treated with HG or PA. (**G**) Representative IF images showing the location of NRF2 in βOHB-supplemented HK-2 cells treated with HG or PA. (**H**, **I**) The mRNA expression of *GCLM*, *GPX4*, and *SOD2* in βOHB-supplemented HK-2 cells stimulated with HG (**H**) or PA (**I**). (**J**) Representative IF images showing the location of NRF2 in kidneys of indicated groups. (**K**) mRNA levels of *Gclm*, *Gpx4*, and *Sod2* in the kidneys of *m/m* group (*n* = 5), *db/db* + Vector (*n* = 4), and *db/db* + AAV-*Bdh1*-GFP group (*n* = 4). All results are representative of three independent experiments. Values are presented as mean ± standard deviation. Bar: 5 μm in **G**, 50 μm and 10 μm in **J**. Abbreviations: BDH1: β-hydroxybutyrate dehydrogenase 1; βOHB: β-hydroxybutyrate; WB: western blot; HG: high glucose; PA: palmitic acid; IF: immunofluorescence. ^*^P < 0.05; ^**^P < 0.01; ^***^P < 0.001.

Given that NRF2 is a well-known transcription factor that regulates the transcriptional induction of antioxidant response element-containing genes encoding antioxidant enzymes in response to cellular stress, including ROS [8–10], we next attempted to determine whether BDH1 mediates anti-ROS function by activating NRF2. Because NRF2 is a nuclear transcription factor, we determined the protein levels of NRF2 in nuclear extracts using western blotting. In HK-2 cells transfected with *Bdh1* siRNA, the cytoplasmic NRF2 protein levels (Supplementary Figure 5A) were significantly up-regulated while the nuclear NRF2 protein levels (Figure 4A) and downstream antioxidant genes (*GCLM*, *GPX4*, and *SOD2*) (Figure 4B) were significantly down-regulated compared with those in cells transfected with normal control (*NC*) siRNA. Moreover, both HG and PA induced a reduction in NRF2 in nuclear extracts, whereas BDH1 overexpression reversed the reduction in HG- and PA-induced NRF2 (Figure 4C) and downstream antioxidative targets (*GCLM*, *GPX4*, and *SOD2*) (Figure 4D, 4E). In contrast, the cytoplasmic NRF2 protein level decreased in BDH1-overexpressed HK-2 cells (Supplementary Figure 5B). Consistent with the observations for BDH1 overexpression, βOHB supplementation reversed HG- and PA-induced NRF2 reductions in nuclear extracts (Figure 4F), which was further confirmed by NRF2 nuclear translocation assay with immunostaining (Figure 4G). Similarly, the expression of NRF2 downstream antioxidant genes, *GCLM*, *GPX4*, and *SOD2* was significantly decreased in both HG- and PA-induced HK-2 cells and reversed by βOHB supplementation (Figure 4H, 4I), which was consistent with the NRF2 nuclear translocation results. In contrast, the cytoplasmic NRF2 protein level was decreased in HK-2 cells supplemented with βOHB (Supplementary Figure 5C) while the *NRF2* mRNA level was not affected in HK-2 cells treated with HG, PA, or βOHB (Supplementary Figure 6). Additionally, AAV9-*Bdh1*-GFP injection promoted nuclear translocation of NRF2 (Figure 4J) and its downstream antioxidant genes (*Gclm*, *Gpx4*, and *Sod2*) (Figure 4K) in the kidneys of *db/db* mice. These data indicate that BDH1-mediated βOHB metabolism promoted NRF2 nuclear translocation.

### Fumarate participates in BDH1-mediated renal protection through the AcAc-succinate-fumarate metabolic pathway

**Figure 5:**
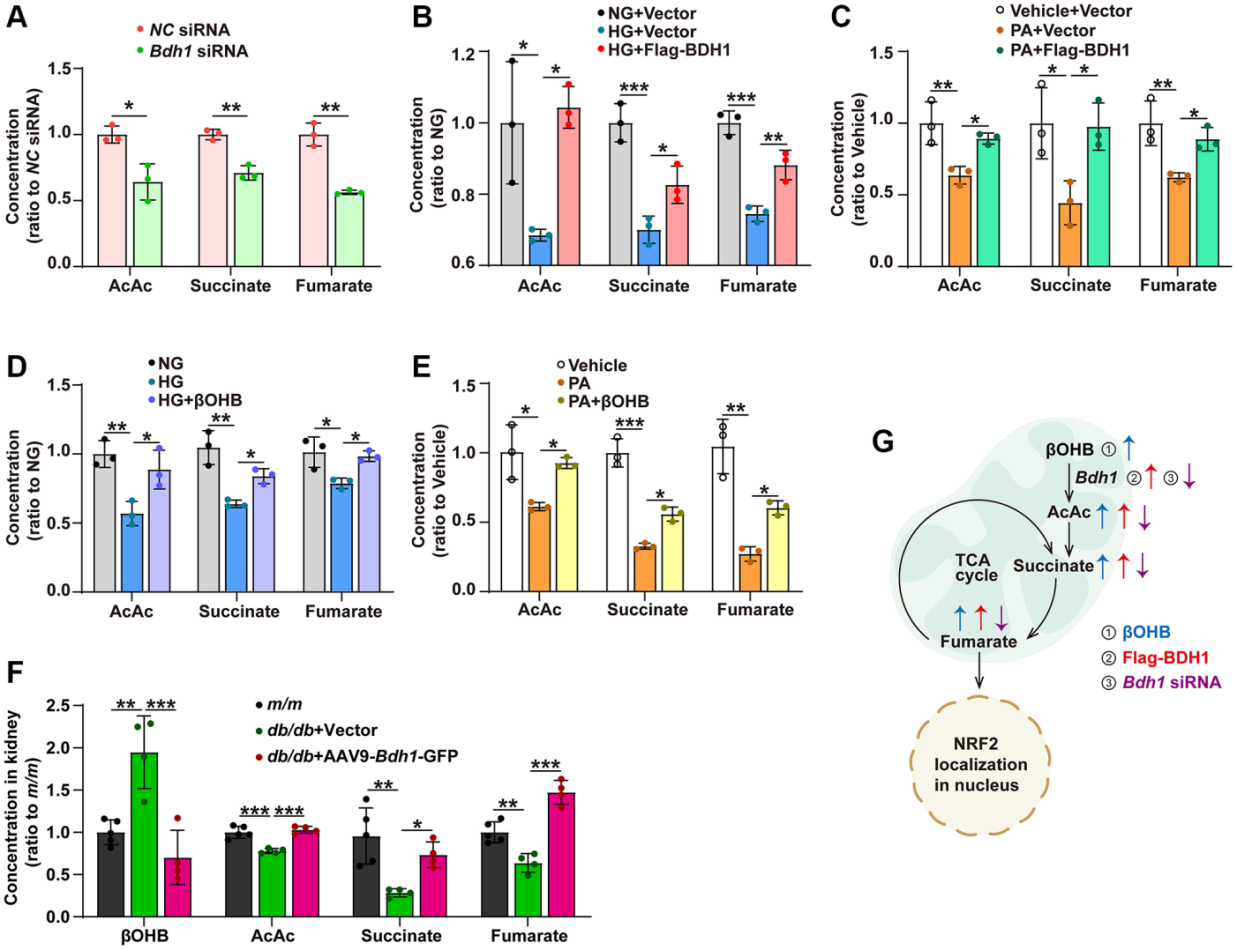
**BDH1-mediated βOHB metabolism promotes fumarate production through the AcAc–succinate–fumarate metabolic pathway.** (**A**) Concentrations of AcAc, succinate, and fumarate in HK-2 cells transfected with *NC* siRNA or *Bdh1* siRNA. (**B**, **C**) Concentrations of AcAc, succinate, and fumarate in BDH1-overexpressed HK-2 cells stimulated with HG (**B**) or PA (**C**). (**D**, **E**) Concentrations of AcAc, succinate, and fumarate in βOHB-supplemented HK-2 cells stimulated with HG (**D**) or PA (**E**). (**F**) Concentrations of βOHB, AcAc, succinate, and fumarate in the kidneys of indicated groups (*n* = 5 in *m/m* group, *n* = 4 in vector, and AAV-*Bdh1*-GFP injected *db/db* group). (**G**) Scheme showing the BDH1-regulated metabolic flux composed of βOHB–AcAc–succinate–fumarate. All results are representative of three independent experiments. Values are presented as mean ± standard deviation. Abbreviations: BDH1: β-hydroxybutyrate dehydrogenase 1; βOHB: β-hydroxybutyrate; AcAc: acetoacetate; TCA: tricarboxylic acid; HG: high glucose; PA: palmitic acid; βOHB: β-hydroxybutyrate; AcAc: acetoacetate. ^*^P < 0.05; ^**^P < 0.01; ^***^P < 0.001.

In the BDH1-mediated βOHB metabolism pathway, BDH1 first metabolizes βOHB into AcAc, which can enter the TCA cycle, and then AcAc is metabolized into succinate and fumarate in turn. Since fumarate is a well-known activator of NRF2 signaling, we examined whether BDH1 activates NRF2 by increasing in fumarate levels. We identified that AcAc, succinate, and fumarate concentrations were reduced in HK-2 cells transfected with *Bdh1* siRNA (Figure 5A). Similar to what was observed in *Bdh1* siRNA-transfected HK-2 cells, both HG and PA treatments reduced the levels of AcAc, succinate, and fumarate, which were successfully blocked by BDH1 overexpression (Figure 5B, 5C). Similarly, βOHB supplementation reversed the HG- or PA-induced reduction of AcAc, succinate, and fumarate (Figure 5D–5E). As the substrate of BDH1, βOHB was reduced in the kidneys of AAV9-*Bdh1*-GFP–injected *db/db* mice (Figure 5F). Consistent with the results observed in HK-2 cells, the levels of AcAc, succinate, and fumarate were reduced in the kidneys of *db/db* mice injected with the vector, although these changes were reversed by AAV9-*Bdh1*-GFP injection (Figure 5F). These findings collectively reveal a metabolic flux composed of βOHB-AcAc-succinate-fumarate that could be regulated by BDH1 or βOHB and affect downstream NRF2 signaling (Figure 5G).

**Figure 6:**
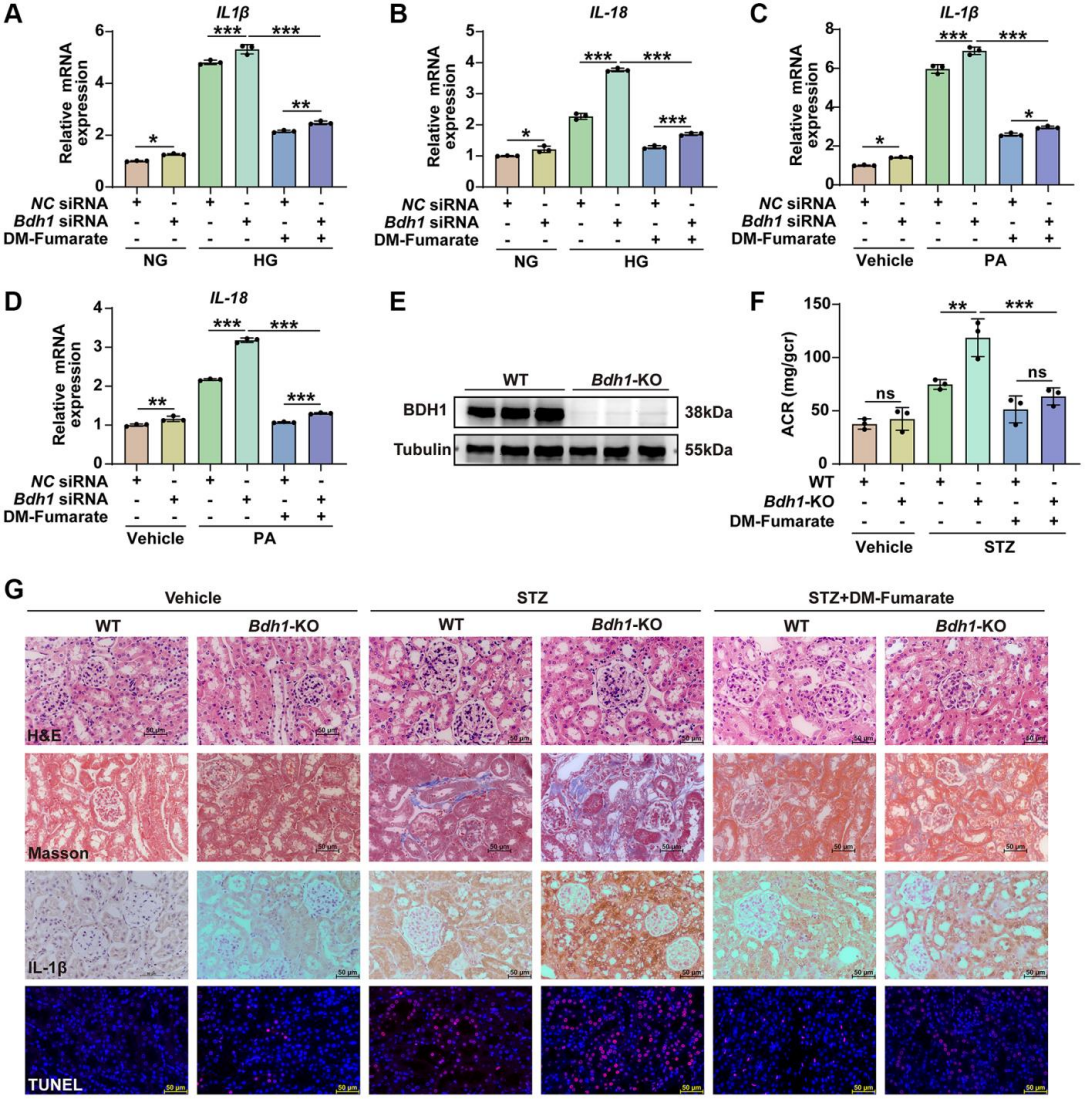
**DM-Fumarate treatment reverses the *Bdh1* deficiency-induced increase of renal inflammation and pathological injury.** (**A**–**D**) qRT-PCR analysis showing the *IL-1β* and *IL-18* mRNA levels of indicated groups (5 μM DM-Fumarate for 48 h). (**E**) Representative WB image showing the protein level of BDH1 in the kidneys of WT and *Bdh1*-KO mice. (**F**) Urinary ACR values of mice in indicated groups (*n* = 3 in per group). (**G**) Representative photomicrographs of H&E, Masson, IHC (IL-1β), and TUNEL staining showing the pathological changes in the kidneys of indicated groups. All results are representative of three independent experiments. Values are presented as mean ± standard deviation. Bar: 50 μm in **G**. Abbreviations: ACR: albumin-to-creatinine ratio; *Bdh1*: β-hydroxybutyrate dehydrogenase 1; DKD: diabetic kidney disease; WB: western blot; H&E: hematoxylin and eosin; IHC: immunohistochemistry; STZ: streptozotocin; DM-Fumarate: dimethyl fumarate. ^*^P < 0.05; ^**^P < 0.01; ^***^P < 0.001; Abbreviation: ns: no significance.

To further determine whether the decrease in fumarate-mediated *Bdh1* reduction-induced aggravation of glucotoxicity and lipotoxicity, we established a glucotoxicity and lipotoxicity cell model using *Bdh1* siRNA-transfected HK-2 cells *in vitro* and a streptozotocin (STZ)-induced diabetes mouse model using *Bdh1* knockout mice *in vivo*, and treated them with dimethyl fumarate (DM-Fumarate). The results showed that the DM-Fumarate treatment reversed the *Bdh1* knockdown-induced increase in glucotoxicity (Figure 6A, 6B) and lipotoxicity (Figure 6C, 6D) in HK-2 cells. Moreover, the DM-Fumarate treatment reversed the *Bdh1* knockout-induced increase in ACR (Figure 6E, 6F) and pathological injury to the kidney (Figure 6G) in STZ-induced diabetic mice. These data indicate that *Bdh1* reduction aggravates glucotoxicity and lipotoxicity by down-regulating fumarate, which provides strong support for our conclusion that *Bdh1* over-expression ameliorate glucotoxicity and lipotoxicity through βOHB-AcAc-succinate-fumarate metabolic flux in DKD.

### βOHB supplementation alleviates the progression of DKD

**Figure 7:**
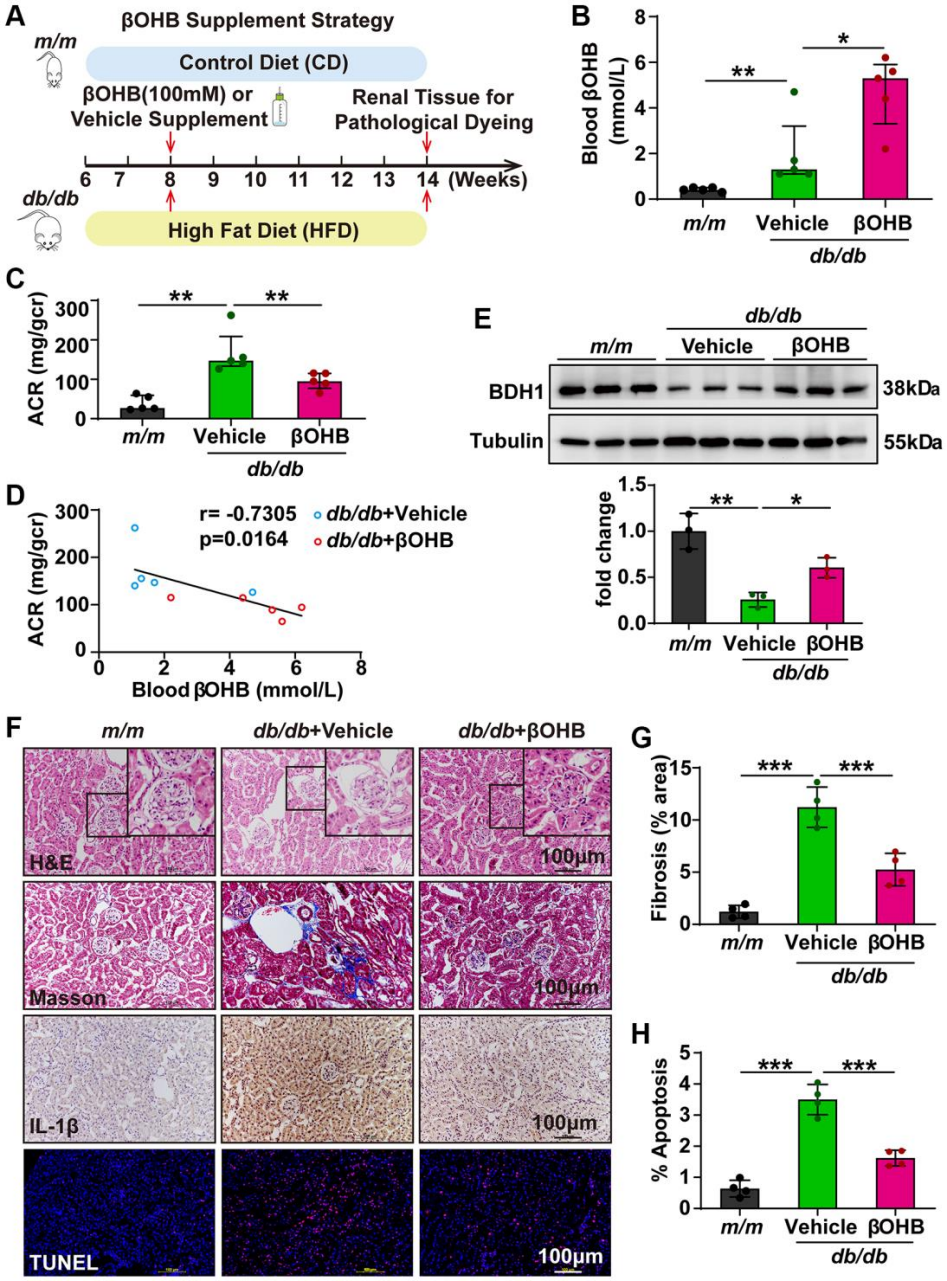
**βOHB supplementation alleviates the progression of DKD.** (**A**) Schematic diagram illustrating the animal experimental design. During the experiment, *m/m* mice were fed with CD, while *db/db* mice were fed with HFD. Eight-week-old male *db/db* mice were randomized to receive vehicle or βOHB, which was dissolved in drinking water at a final concentration of 100 mM. Mice were euthanized at six weeks after βOHB intervention. (**B**) Blood level of βOHB in mice from indicated groups (*n* = 5 per group). (**C**) Urinary ACR values of mice from indicated groups (*n* = 5 per group). (**D**) Negative correlation between serum βOHB level and ACR in *db/db* mice with vehicle and βOHB treatment. (**E**) Representative WB image showing the protein level of BDH1 in the kidneys of indicated groups. (**F**) Representative photomicrographs of H&E, Masson, IHC (IL-1β), and TUNEL staining showing the pathological changes in the kidneys of indicated groups. (**G**) Quantification of the fibrosis area in the kidneys of indicated groups (*n* = 4 per group). (**H**) Quantification of apoptosis-positive cells in the kidneys of indicated groups (*n* = 4 per group). All results are representative of three independent experiments. In **B** and **C**, values are presented as median (interquartile range). In **E**, **G**, and **H**, values are presented as mean ± standard deviation. Bar: 100 μm in **F**. Abbreviations: CD: control diet; HFD: high fat diet; ACR: albumin-to-creatinine ratio; BDH1: β-hydroxybutyrate dehydrogenase 1; βOHB: β-hydroxybutyrate; DKD: diabetic kidney disease; WB: western blot; H&E: hematoxylin and eosin; IHC: immunohistochemistry. ^*^P < 0.05; ^**^P < 0.01; ^***^P < 0.001.

As βOHB supplementation showed a similar effect as BDH1 overexpression on HG- or PA-induced ROS overproduction and inflammation in HK-2 cells, we next attempted to determine whether βOHB supplementation could ameliorate DKD. The experimental strategy is illustrated in Figure 7A. At six weeks after supplementation of βOHB (100 mM) in drinking water, we identified the blood βOHB levels and observed an increased βOHB levels in *db/db* mice supplemented with βOHB (Figure 7B). Subsequently, an ACR assay was performed. Although βOHB supplementation did not affect body weight, fasting blood glucose levels (Supplementary Figure 4C, 4D), and the kidney-to-body weight ratio (Supplementary Table 3), we observed that ACR was significantly lower in *db/db* mice supplemented with βOHB compared with mice supplemented with vehicle (Figure 7C). Moreover, the serum level of βOHB was negatively correlated with the value of ACR, indicating a strong ACR reduction capability of βOHB in DKD (Figure 7D). We observed increased BDH1 expression in the kidneys of *db/db* mice treated with βOHB (Figure 7E). Further histological analysis revealed that *db/db* mice supplemented with βOHB showed a normal glomerular morphology whereas *db/db* mice supplemented with vehicle showed glomerular hypertrophy (Figure 7F). Consistent with AAV9-mediated BDH1 renal expression, DKD pathology-related fibrosis, inflammation, and apoptosis were substantially reduced by βOHB supplementation (Figure 7F–7H), whereas the expression of downstream targets of NRF2 was elevated (Supplementary Figure 7A). These results indicate that βOHB supplementation might ameliorate DKD by increasing the renal expression of BDH1, which promotes βOHB metabolism.

### Ketogenic diet alleviates the progression of DKD

**Figure 8:**
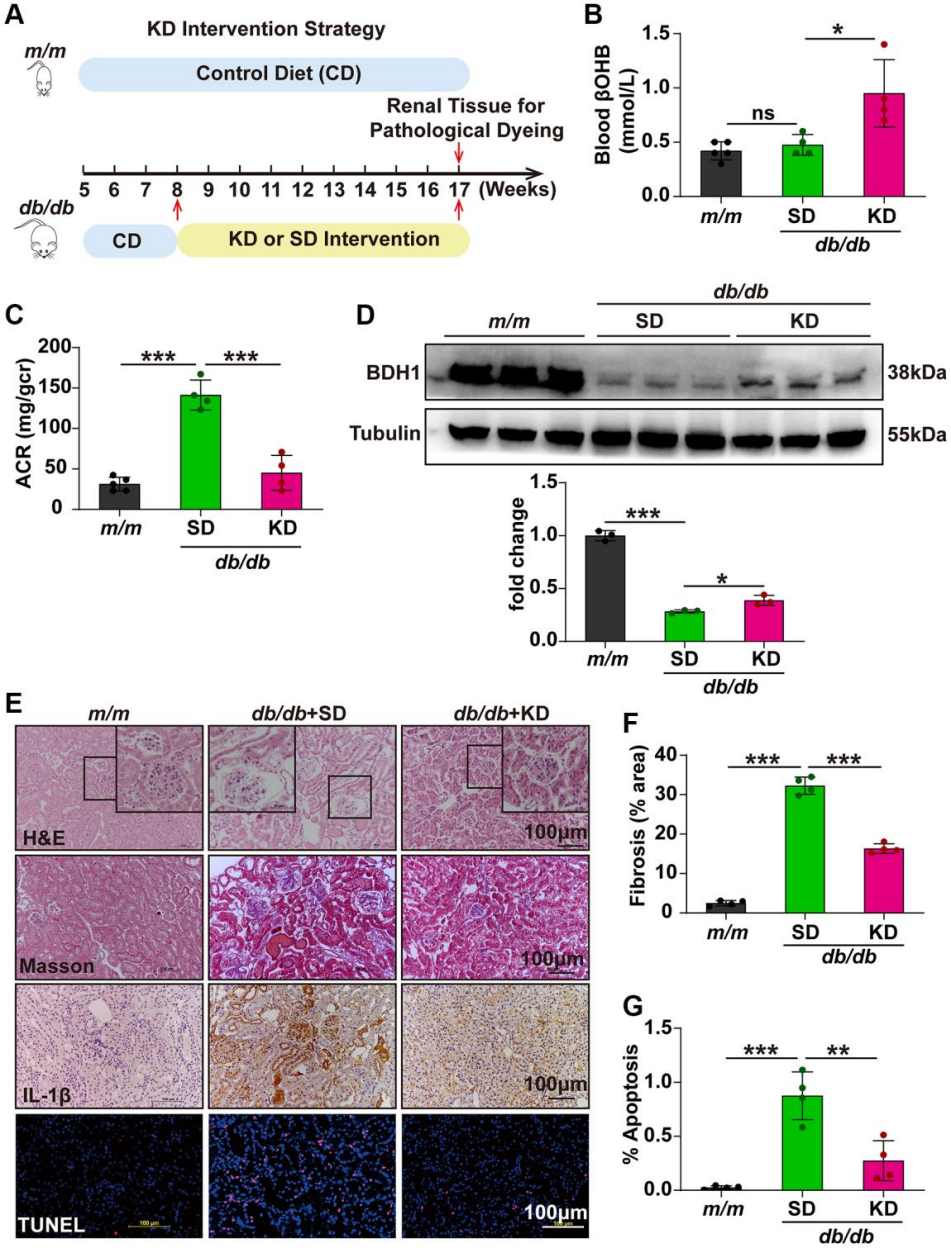
**Ketogenic diet alleviates the progression of DKD.** (**A**) Schematic diagram illustrating the animal experimental design. During the experiment, *m/m* mice were fed with CD, while *db/db* mice were fed with SD or KD starting at eight-week-old. Mice were euthanized at nine weeks after KD intervention. A schematic representation of the KD intervention strategy in *db/db* mice. (**B**) Blood level of βOHB in mice from indicated groups (*n* = 5 in *m/m* group; *n* = 4 in SD and KD groups). (**C**) Urinary ACR values of mice in indicated groups (*n* = 5 in *m/m* group; *n* = 4 in SD and KD groups). (**D**) Representative WB image showing the protein level of BDH1 in the kidneys of indicated groups. (**E**) Representative photomicrographs of H&E, Masson, IHC (IL-1β), and TUNEL staining showing the pathological changes in the kidneys of indicated groups. (**F**) Quantification of the fibrosis area in the kidneys of indicated groups (*n* = 4 per group). (**G**) Quantification of apoptosis-positive cells in the kidneys of indicated groups (*n* = 4 per group). All results are representative of three independent experiments. Values are presented as mean ± standard deviation. Bar: 100 μm in **E**. Abbreviations: ACR: albumin-to-creatinine ratio; SD: standard diet; KD: ketogenic diet; DKD: diabetic kidney disease; BDH1: β-hydroxybutyrate dehydrogenase 1; βOHB: β-hydroxybutyrate; WB: western blot; H&E: hematoxylin and eosin; IHC: immunohistochemistry. ^*^P < 0.05; ^**^P < 0.01; ^***^P < 0.001. Abbreviation: ns: no significance.

KD has been widely used in clinical studies and reported to have antidiabetic effects; however, the underlying mechanisms have not been fully described. Because KD mainly produces βOHB and BDH1-mediated βOHB metabolism plays a protective role in DKD, we next attempted to determine whether KD could ameliorate DKD and identify whether KD functions through the BDH1-mediated βOHB metabolism pathway. As shown in Figure 8A, *m/m* and *db/db* mice were fed a standard diet (SD) or KD for nine weeks starting at eight weeks of age. Although KD feeding did not affect the body weight (Supplementary Figure 4E), the fasting blood glucose levels returned to normal levels (Supplementary Figure 4F) and the kidney-to-body weight ratio was significantly reduced in KD-fed *db/db* mice (Supplementary Table 3). Compared with SD-fed *db/db* mice, KD-fed *db/db* mice showed increased blood levels of βOHB (Figure 8B). The KD treatment nearly completely reversed the increase in ACR in SD-fed *db/db* mice (Figure 8C). We observed increased BDH1 expression in the kidneys of the KD-fed *db/db* mice (Figure 8D). Further histological analysis showed that the KD-fed *db/db* mice showed significant pathological remission in the kidneys, including fibrosis, inflammation, and apoptosis (Figure 8E–8G), whereas the expression of downstream targets of NRF2 was elevated (Supplementary Figure 7B). These results indicate that KD might ameliorate DKD by increasing blood βOHB and renal expression of BDH1, which ultimately promotes βOHB metabolism.

## DISCUSSION

We improved this study based on the results discussed in our previous preprint [35] on the Research Square platform. In this study, we demonstrated that BDH1 in renal cells is a potential therapeutic target for DKD. We showed that the expression of BDH1 was reduced in DKD and HG- or PA-treated HK-2 cells. BDH1 overexpression or βOHB treatment protects HK-2 cells from glucotoxicity and lipotoxicity. Mechanistically, BDH1-mediated βOHB metabolism inhibits oxidative stress by activating NRF2 via fumarate production upregulation. Of note, AAV9-mediated *Bdh1* renal expression, βOHB supplementation, and KD feeding can reverse fibrosis, inflammation, and apoptosis in DKD. Thus, the finding of BDH1-mediated βOHB metabolism in the kidney may lead to new treatments for DKD.

In the kidneys, renal proximal tubular epithelial cells have a reabsorption function and play an important role in the pathogenesis of DKD. However, the molecular mechanisms underlying the effect of tubular epithelial cells on DKD remain unclear. *Bdh1* mRNA and protein expression was much higher in HK-2 cells than that in Mpc5 cells (podocyte cell line) and SV40-MES cells (glomerular mesangial cell line) (Supplementary Figure 8). Thus, a renal tubular BDH1-mediated mechanism involved in the progression of DKD has been identified.

In the pathophysiology of diabetic kidney disease, increased oxidant species have been identified as a single unifying upstream event that plays a central role in the progression of DKD [36]. *Nrf2*, a master positive regulator for genes related to antioxidant effects, is negatively regulated by kelch-like epichlorohydrin-associated protein 1 (*Keap1*) [37]. Thus, clinical trials on *Keap1* inhibitors or *Nrf2* inducers have been conducted for the treatment of DKD [38]. In addition, fumarate is known to inhibit the binding of KEAP1 to NRF2 by modifying cysteine residues [12, 26]. In this study, we identified that BDH1 regulates the activity of NRF2 by modulating fumarate levels during the progression of DKD. We showed that BDH1 overexpression upregulated the fumarate level by promoting a metabolic flux composed of βOHB-AcAc-succinate-fumarate (Figure 5).

In recent years, gene therapy has emerged as a novel therapeutic modality with the potential to cure serious diseases. As a gene delivery vector, AAV has achieved preclinical and clinical success in the treatment of human diseases through gene replacement, gene silencing, and gene editing, and it has been identified as a safe, well-tolerated, and effective therapeutic vector [39]. The US Food and Drug Administration recently approved AAV-based gene therapy for infant GM1 ganglioside storage disease and frontotemporal lobe dementia caused by granule protein mutation [40]. However, reports on AAV-based gene therapy for DKD are lacking. In this study, we successfully observed the renal expression of GFP by intravenous vector injection, indicating that AAV9 is an effective gene delivery tool for kidney targets in mice (Figure 2). Moreover, AAV9-mediated *Bdh1* renal expression significantly inhibited DKD progression in *db/db* mice. Although AAV9 is not a kidney-specific vector and the delivery tool for kidney targets are not as efficient as that for heart and liver targets [41], the effective amelioration of DKD observed in AAV9-*Bdh1*-GFP-injected *db/db* mice strongly suggests that AAV9-mediated gene-targeting therapy is a promising treatment.

Ketone body metabolism refers to the processes of liver production (ketogenesis) and utilization in extrahepatic organs (ketolysis) [25]. KD is a diet with high fat and low carbohydrate contents, and they can stimulate fasting metabolism and place the body in a state of ketogenesis [30]. However, the roles of ketone bodies and KD in various diseases remain unclear and even contradictory. βOHB and KD are clinically beneficial in several common human neurodegenerative diseases [26] and have been reported to ameliorate hyperglycemia, mitochondrial dysfunction, and cardiomyopathy in *db/db* mice [18]. Furthermore, KD was reported to reverse diabetic kidney disease in T1DM and T2DM mouse models as early as 2011 [24], which is consistent with our data (Figure 8). However, the underlying mechanism is still undetermined. In another clinical study, increased levels of ketone bodies were reported to promote DKD progression in patients with diabetes [42]. Feeding a KD promoted liver fibrosis and led to liver damage in a T2DM mouse model [43]. Furthermore, high protein intake under a KD may accelerate the progression of kidney disease [44]. In this study, we showed that either βOHB supplementation or KD feeding could alleviate the progression of renal fibrosis, inflammation, and apoptosis in *db/db* mice (Figure 8). Both βOHB supplementation and KD feeding elevated renal expression of *Bdh1*, which functions as a strong antioxidant by activating NRF2. Unexpectedly, in our study, the serum levels of βOHB in vehicle-treated *db/db* mice (Figure 7B) appeared to be consistent with those in KD-fed *db/db* mice (Figure 8B), which may have been related to the reversal of hyperglycemia to nearly normal levels in *db/db* mice (Supplementary Figure 4F) after five weeks of a KD. Given that hyperglycemia in diabetes is the main factor promoting ketogenesis, the KD induced normal blood glucose levels, as shown in Figure 8B, which inhibited endogenous ketogenesis compared to hyperglycemia in *db/db* mice, as shown in Figure 7B. We further confirmed the protective effect of βOHB and KD on DKD and identified a *Bdh1*-mediated mechanism. Collectively, βOHB and KD might have unknown side effects when applied to protect individuals from disease, which further suggests that BDH1-targeted therapy is a more appropriate treatment.

**Figure 9:**
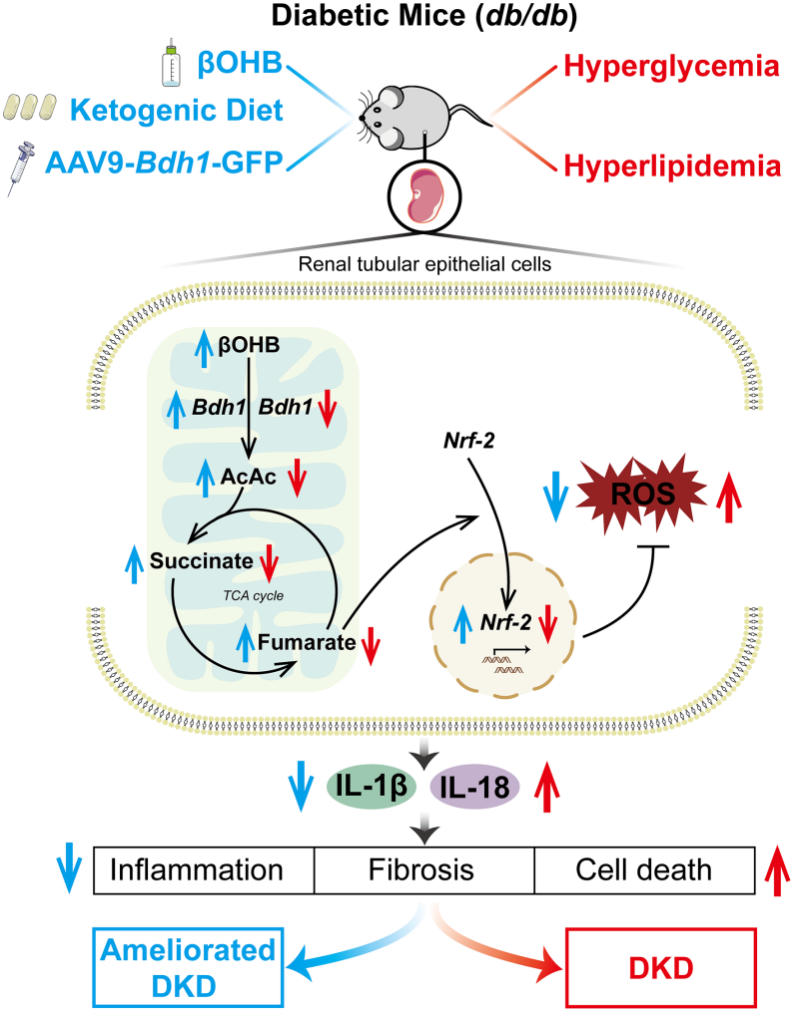
**Schematic diagram depicting the mechanism by which BDH1-mediated βOHB metabolism ameliorates DKD.** Abbreviations: BDH1: β-hydroxybutyrate dehydrogenase 1; βOHB: β-hydroxybutyrate; DKD: diabetic kidney disease.

In generally, our results provide evidence for the proposed mechanism, as shown in Figure 9. Hyperglycemia and hyperlipidemia in *db/db* mice downregulated the expression of BDH1, which subsequently downregulated the fumarate level via βOHB-AcAc-succinate-fumarate metabolic flux. Fumarate reduction decreased the nuclear translocation of NRF2, a key ROS inhibitor. ROS overproduction finally activated DKD-related inflammation, fibrosis, and apoptosis in the kidneys. Our findings highlight the feasibility of inducing BDH1 renal expression, administering βOHB, and feeding KD as treatments for attenuating DKD.

## MATERIALS AND METHODS

### Animals

*Bdh1* knock-out (KO) mice with a C57BL/6J background were generated by Cyagen (Cyagen Biosciences, Guangzhou, China). Eight-week-old male *Bdh1*-KO mice and their wild-type (WT) littermates received i.p. injections of low-dose STZ (50 mg/kg; S8050, Solarbio, Beijing, China). The STZ injection was repeated daily for five consecutive days. Five days after the final STZ injection, mice with non-fasting blood glucose >16.7 mmol/L (as measured with the Accu-Chek Advantage blood glucose monitoring system; Roche Diagnostics, Basel, Switzerland) were considered diabetic. Mice treated with sodium citrate buffer were used as controls. Two weeks after STZ injection, mice were randomized to receive DM-Fumarate (15 mg/kg body weight twice daily) or vehicle via oral gavage, as previously described [14]. Three mice were randomly assigned to each treatment group. Mice were euthanized eight weeks after STZ injection.

Five-week-old C57 BKS *db/db* male mice (*n* = 20; 31.94 ± 2.08 g) and *db/db* littermate controls (*m/m*; *n* = 5; 19.96 ± 0.79 g) were purchased from GemPharmatech Co., Ltd., (Nanjing, China). Animals were acclimatized for at least one week before the experiments and then randomly divided into five groups (*m/m*, *db/db*+AAV9-Vector, *db/db*+AAV9-*Bdh1*-GFP, *db/db*+standard diet (SD), and *db/db*+KD; *n* = 5 in each group). A 60% high-fat diet was purchased from Research Diets, Inc. (D12492, USA), SD (TP201442) and KD (74.2% fat, 8.9% protein, and 3.2% carbohydrates; TP201414) were purchased from Trophic Animal Feed High-Tech Co., Ltd., (Jiangsu, China). For the βOHB supplementation experiment, 5-week-old C57 BKS *db/db* male mice (*n* = 10; 31.08 ± 1.51 g) and *db/db* littermate controls (*m/m*; *n* = 5; 18.7 ± 1.32 g) were purchased from GemPharmatech Co., Ltd., (Nanjing, China). Animals were acclimatized before the experiments for at least one week and then randomly divided into three groups (*m/m*, *db/db*+Vehicle, and *db/db*+βOHB; *n* = 5 in each group). All animal experiments were performed under the following conditions: room temperature 23°C ± 1°C, relative humidity 60% ± 10%, and alternating 12 h light–dark cycles in individually ventilated cages. The animal experiments were approved by the Institutional Animal Ethics Committee of Southwest Medical University and conducted in accordance with the National Institutes of Health Guide for the Care and Use of Laboratory Animals.

### Human renal samples

Samples from patients who had been diagnosed with DKD were collected at the Department of Pathology of the Affiliated Hospital of Southwest Medical University. Normal samples were collected from individuals without diabetes or renal disease who had undergone tumor nephrectomy. The study was conducted in accordance with the principles of the Declaration of Helsinki and was approved by the Research Ethics Committee of The Affiliated Hospital of Southwest Medical University. Informed consent was obtained from all patients.

### Animal experiments

For AAV9-mediated *Bdh1* renal expression, 100 μL of pAAV-ITR-CAG-*Bdh1*-IRES-EGFP-WPRE-Sv40polyA-ITR (AAV9-*Bdh1*-GFP, 3.40E+12 vg/mL, Beijing Syngentech Co., Ltd., Beijing, China) and vector (1.90E+13 vg/mL) was injected into *db/db* or *m/m* mice via the caudal vein. For βOHB supplementation, to reduce the risk of accidental death of mice, sodium β-hydroxybutyrate (CAS:150-83-4; Shanghai Macklin Biochemical Co., Ltd., Shanghai, China) was dissolved in drinking water at a final concentration of 100 mM as previously described [45] and supplied to the *db/db* mice. Body weights and blood glucose levels were recorded weekly.

### HK-2 cell culture

HK-2 cells (ATCC, USA) were cultured in Dulbecco’s Modified Eagle Medium (HyClone, Logan, UT, USA) containing 10% fetal bovine serum (ScienCell, Carlsbad, CA, USA) and supplemented with 1% penicillin–streptomycin (Beyotime, Shanghai, China). HK-2 cells were cultured at 37°C with 5% CO_2_ until 60–70% confluence [46]. Then, the cells were treated with normal glucose (NG; 5.5 mM), mannitol (MA; 24.5 mM), high glucose (HG; 30 mM), vehicle (40 μM BSA), palmitic acid (PA; 200 μM, Sigma-Aldrich, Saint Louis, MO, USA), βOHB (5 mM), and DM-Fumarate (5 μM) for 48 h. PA [47], βOHB [48], and DMF [49] were prepared as previously described.

### RNA-seq study and analysis

Total RNA from renal tissues (*n* = 3 each for the *m/m* and *db/db* groups) was extracted separately using TRIzol Reagent (Invitrogen, Carlsbad, CA, USA) following the manufacturer’s procedure. All purified libraries were sequenced using an Illumina NovaSeq 6000 (LC Sciences, CA, USA) at Shanghai Majorbio Bio-Pharm Technology Co., Ltd., (Shanghai, China). Gene expression was normalized to the number method of fragments per kilobase per million reads. The resulting *P*-values were adjusted following the Benjamini and Hochberg method [50] to regulate the false discovery rate. DEGs were identified based on an adjusted *P*-values of < 0.05 and | log2 (fold change) | ≧1.5. To evaluate significantly enriched metabolic or signal transduction pathways, pathway enrichment analysis was performed using the KEGG database (version 2017.08) [51, 52]. Categories with false discovery rates (FDRs) <0.05 were considered significant KEGG pathways. Raw sequencing data are available from the NCBI BioProject SRA database (PRJNA876094).

### Histopathological examination

Kidney tissues were fixed in 4% paraformaldehyde for 24 h, embedded in paraffin, and sectioned to a thickness of 4-μm. The sections were stained with hematoxylin and eosin (H&E) or Masson’s trichrome for light microscopy and morphometry.

### Immunohistochemistry staining

Briefly, 4-μm-thick paraffin sections were dewaxed, hydrated, and stained with primary antibodies against BDH1 (1:100, ab193156, Abcam, UK) and IL-1β (1:100, #12242, Cell Signaling Technology, USA). The sections were stained with biotin-labeled goat anti-rabbit IgG or biotin-labeled anti-mouse IgG and then treated with horseradish enzyme-labeled oopaltin from *Streptomyces* (Beijing ZSGB Biological Technology Co., Ltd., Beijing, China). The stained sections were scanned under a light microscope.

### Immunofluorescence staining

IF staining of kidney paraffin sections and HK-2 cells was performed using anti-BDH1 antibody (1:100) and NRF2 (1:50, sc518033, Santa Cruz, USA). The membranes were then incubated with Cy3/FITC fluorescent dye-conjugated secondary antibodies (1:200 dilution, Biosynthesis Biotech, USA) for 60 min at room temperature in the dark. Nuclei were labeled with DAPI, and images were obtained using a fluorescence microscope (Leica, Wetzlar, Germany).

### ROS and TUNEL assay

ROS levels in HK-2 cells were measured using a DCFH-DA fluorescent probe according to the manufacturer protocol (Beyotime, Shanghai, China). TUNEL staining of the kidney paraffin sections was performed according to the TUNEL Kit protocol (Roche, Indianapolis, IN, USA).

### Human *Bdh1* cDNA transfection

Human *Bdh1*-overexpressed plasmid (pCMV3-BDH1-Flag) and vector plasmid (pCMV3) were purchased from Beijing Sino Biological, Inc., (Beijing, China) and transfected into HK-2 cells using Lipofectamine 3000 (Invitrogen).

### SiRNA transfection

The siRNAs for *BDH1* (stB0005692B) and *NC* (siN0000001-1-5) were purchased from RiboBio (Guangzhou, China). The siRNA sequence of the human *BDH1* gene was 5′-GCCTAAACAGTGACCGATT-3′. The *BDH1* and *NC* siRNA were transfected into HK-2 cells with ribo*FECT*™ CP Reagent and ribo*FECT*™ CP Buffer (RiboBio, Guangzhou, China).

### Measurement of βOHB, AcAc, succinate, and fumarate

The βOHB content was measured using mouse βOHB ELISA kits (F9242-A, Shanghai Fankel Industrial Co., Ltd., Shanghai, China). AcAc content was measured using human acetoacetate ELISA kits (JL15388, Shanghai J&L Biological, Shanghai, China) and mouse acetoacetate ELISA kits (2M-KMLJM220724m, Nanjing Camilo Bioengineering Co., Ltd., Nanjing, China). The succinate and fumarate contents were measured using Succinate Assay kits (MAK335, Sigma-Aldrich, Saint Louis, MO, USA) and Fumarate Assay kits (MAK060, Sigma-Aldrich, Saint Louis, MO, USA) according to the manufacturer’s instructions.

### qRT-PCR analysis

Total RNA from renal tissue and HK-2 cells was extracted using TRIzol reagent (Invitrogen, USA). ReverTra Ace qPCR RT Master Mix (FSQ-201, Toyobo, Japan) was used for reverse transcription, and a QuantiNova SYBR Green PCR Kit (QIAGEN, Germany) was used for qRT-PCR. qRT-PCR was performed with Analytikjena qTOWER 3G real-time PCR system (Analytik Jena, Jena, Germany) according to the manufacturer’s instructions. All primers used in this study are listed in Supplementary Table 4. β-actin was used as an internal reference gene to normalize target gene expression. All samples were measured in triplicate. The 2^−ΔΔCt^ method [53] was used to calculate gene expression relative to that of the reference gene.

### Western blot analysis

Total proteins from kidneys of mice and HK-2 cells were extracted using an extraction buffer (RIPA). Nuclear proteins were extracted using a Nucleoprotein Extraction Kit (Shanghai Sangon Biotech, Shanghai, China). Protein samples were separated using sodium dodecyl sulfate–polyacrylamide gel electrophoresis and transferred onto a PVDF membrane (Millipore, Burlington, MA, USA). The membranes were incubated with 5% BSA to block other contaminants and then with primary antibodies. Immunoblotting was performed using anti-BDH1 antibody (1:1000 dilution), anti-NRF2 antibody (1:500 dilution), anti-IL-1β antibody (1:1000 dilution), anti-β-actin antibody (1:4000 dilution, AF0003, Beyotime, China), anti-Tubulin antibody (1:4000 dilution, AF0001, Beyotime, China), anti-Histone H3 antibody (1:1000 dilution, AF0009, Beyotime, China), and anti-Flag antibody (1:1000 dilution, AF5051, Beyotime, China).

### Statistical analysis

All *in vitro* experiments were repeated three times. ImageJ software (version 1.80; National Institutes of Health, USA) was used for western blot analysis. Data were expressed as the mean ± standard deviation or median (interquartile range). Statistical analyses and figure generation were performed using GraphPad Prism 9. Continuous variables were analyzed using the two-tailed unpaired Student’s *t*-test or the Mann-Whitney *U* test, followed by normality tests for data from two groups. One-way ANOVA tests were performed for multiple comparisons of values, followed by Tukey’s test. Differences at *P* < 0.05 were considered statistically significant. Statistical significance was set at ^*^P < 0.05, ^**^P < 0.001, *P* < 0.01, and ^***^P < 0.001.

### Data availability

Data supporting the findings of this study are available from the corresponding author upon reasonable request.

## Supplementary Materials

Supplementary Figures

Supplementary Table 1

Supplementary Tables 2-4
