# Supplementary material for: BDH1-mediated βOHB metabolism ameliorates diabetic kidney disease by activation of NRF2-mediated antioxidative pathway
Source: Aging (Albany NY). 2023 Nov 27;15(22):13384–410. doi: 10.18632/aging.205248 (PMC11622694; doi:10.18632/aging.205248)
Supplement: Supplementary Figures [file aging-15-205248-s001.pdf]

SUPPLEMENTARY FIGURES

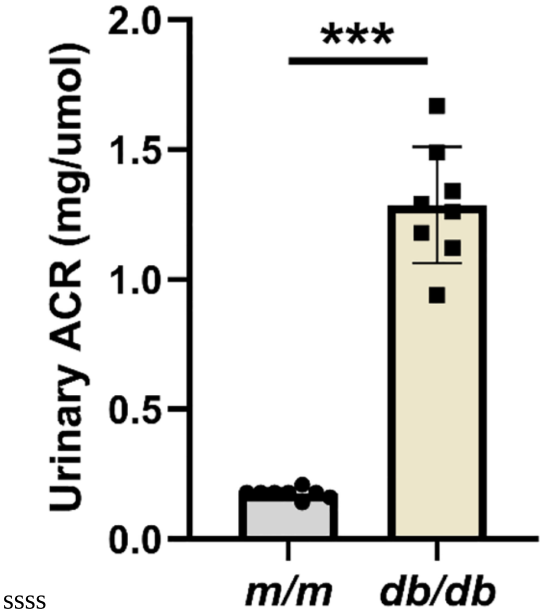

ssss

Supplementary Figure 1. Urinary ACR in *m/m* and *db/db* group (*n* = 8 in per group). Abbreviation: ACR: albumin-to-creatinine ratio. ***$P < 0.001$.

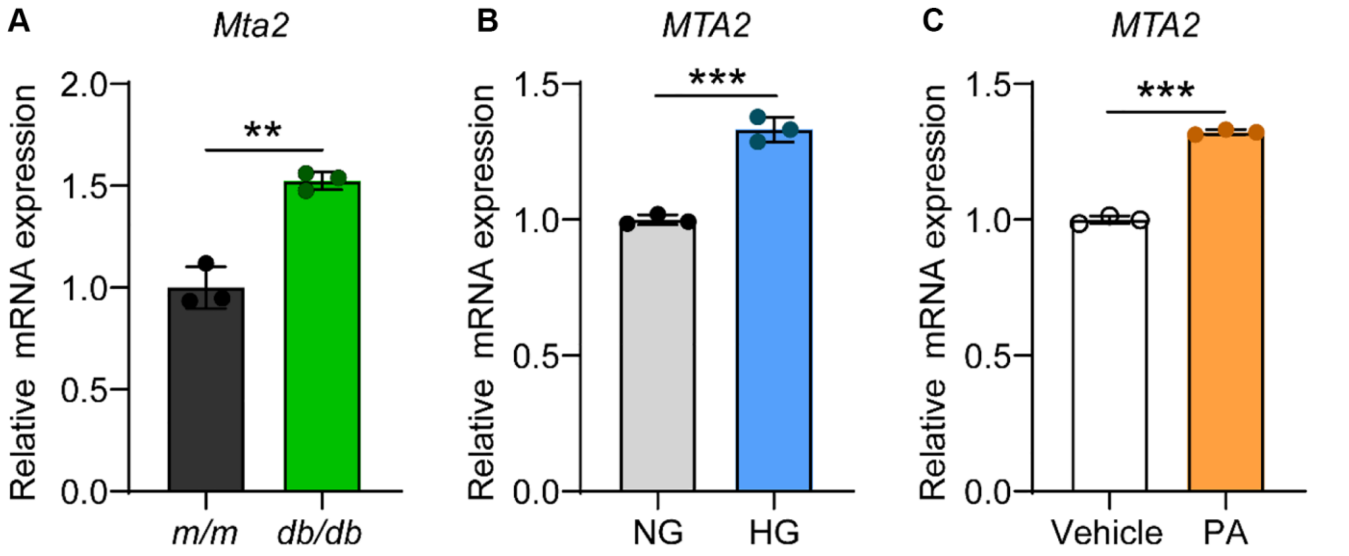

Supplementary Figure 2. qRT-PCR analysis showing the mRNA level of *Mta2* in the kidneys (**A**) of *m/m* and *db/db* mice (*n* = 3 per group) and HK-2 cells treated with HG (**B**) or PA (**C**). All results are representative of three independent experiments. Values are presented as mean ± standard deviation. **$P < 0.01$; ***$P < 0.001$.

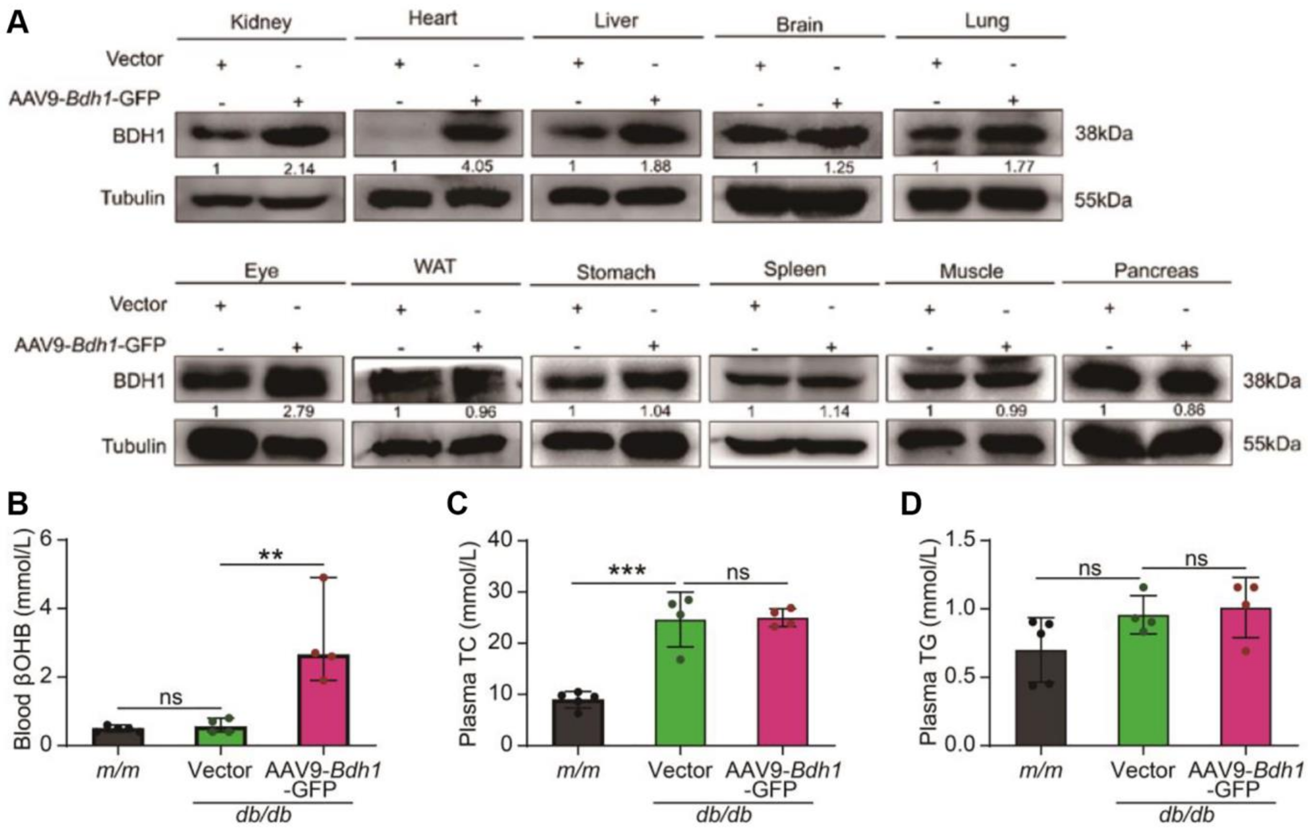

Supplementary Figure 3. (**A**) BDH1 protein expression levels after AAV9-*Bdh1*-GFP injection in all major organs in the mice body were detected. (**B**) Blood level of βOHB in mice from the indicated groups (n = 5 in *m/m* group, n = 4 in vector, and AAV-*Bdh1*-GFP injected db/db group). (**C**) Plasma TC in mice from the indicated groups (n = 5 in *m/m* group, n = 4 in vector, and AAV-*Bdh1*-GFP injected *db/db* group). (**D**) Plasma TG in mice from the indicated groups (n = 5 in *m/m* group, n = 4 in vector, and AAV-*Bdh1*-GFP injected *db/db* group). Abbreviations: BDH1: β-hydroxybutyrate dehydrogenase 1; TC: total cholesterol; TG: total triglyceride. $^{**}P < 0.01$; $^{***}P < 0.001$; Abbreviation: ns: no significance.

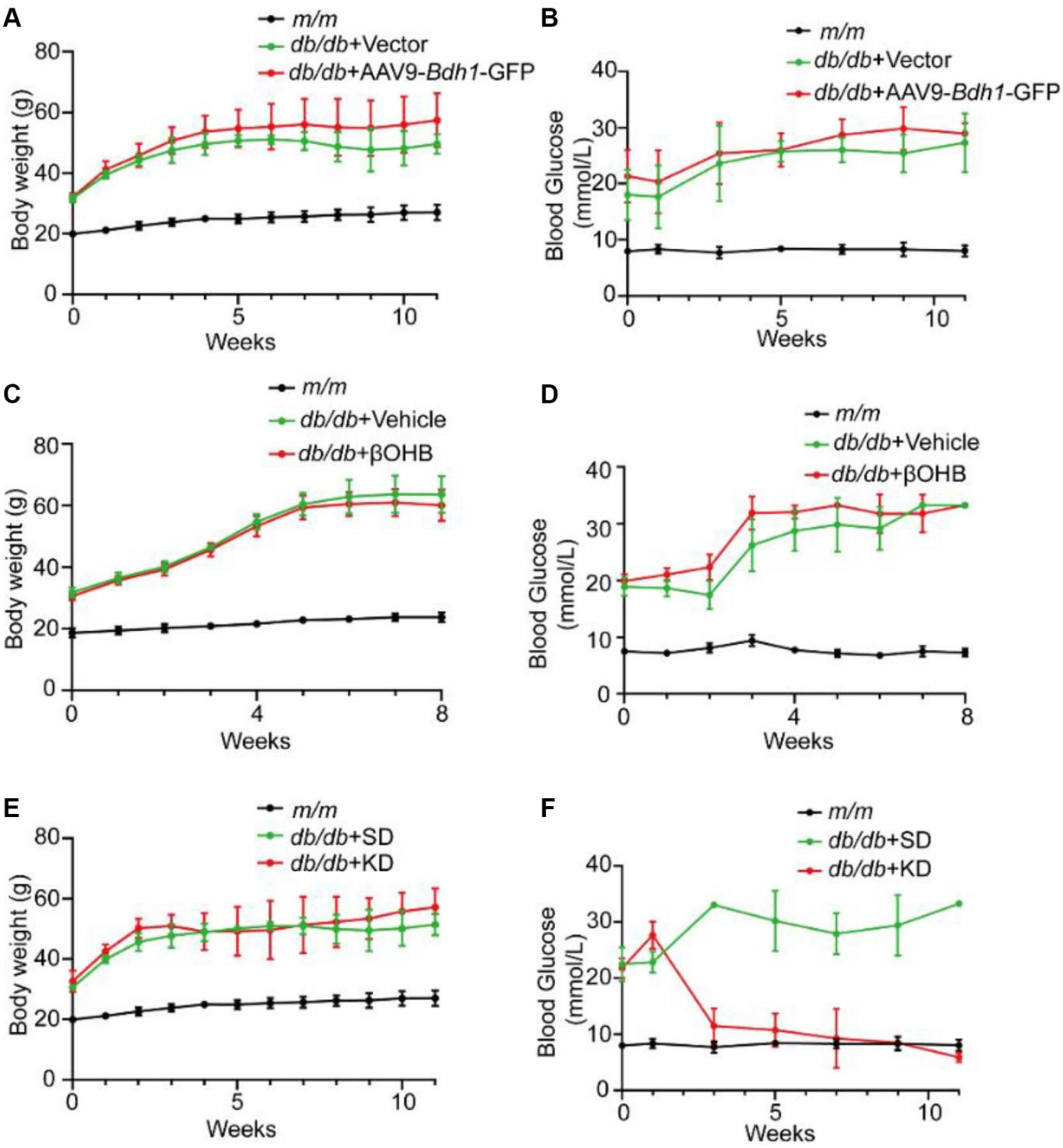

Supplementary Figure 4. Body weight (**A**) and fasted blood glucose (**B**) of mice in the indicated groups (*n* = 5 in *m/m* group, *n* = 4 in vector, and AAV-*Bdh1*-GFP injected *db/db* group). Body weight (**C**) and fasted blood glucose (**D**) of mice in the indicated groups (*n* = 5 per group). Body weight (**E**) and fasted blood glucose (**F**) of mice in the indicated groups (*n* = 5 in *m/m* group; *n* = 4 in SD and KD groups).

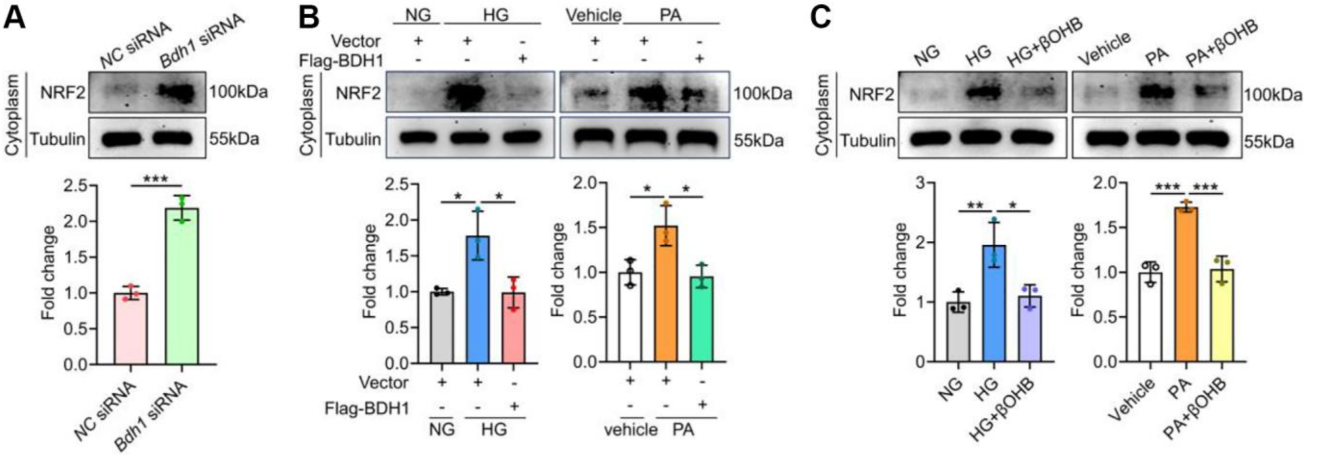

Supplementary Figure 5. (**A**) Representative WB image showing the protein level of NRF2 protein in the cytoplasm of HK-2 cells transfected with *NC* siRNA or *Bdh1* siRNA. (**B**) Representative WB images showing the protein level of NRF2 in the cytoplasm of BDH1-overexpressed HK-2 cells treated with HG or PA. (**C**) Representative WB images showing the protein level of NRF2 in the cytoplasm of βOHB-supplemented HK-2 cells treated with HG or PA. All results are representative of three independent experiments. Values are presented as mean ± standard deviation. Abbreviations: BDH1: β-hydroxybutyrate dehydrogenase 1; βOHB: β-hydroxybutyrate; WB: western blot; HG: high glucose; PA: palmitic acid. $^{*}P < 0.05$; $^{**}P < 0.01$; $^{***}P < 0.001$.

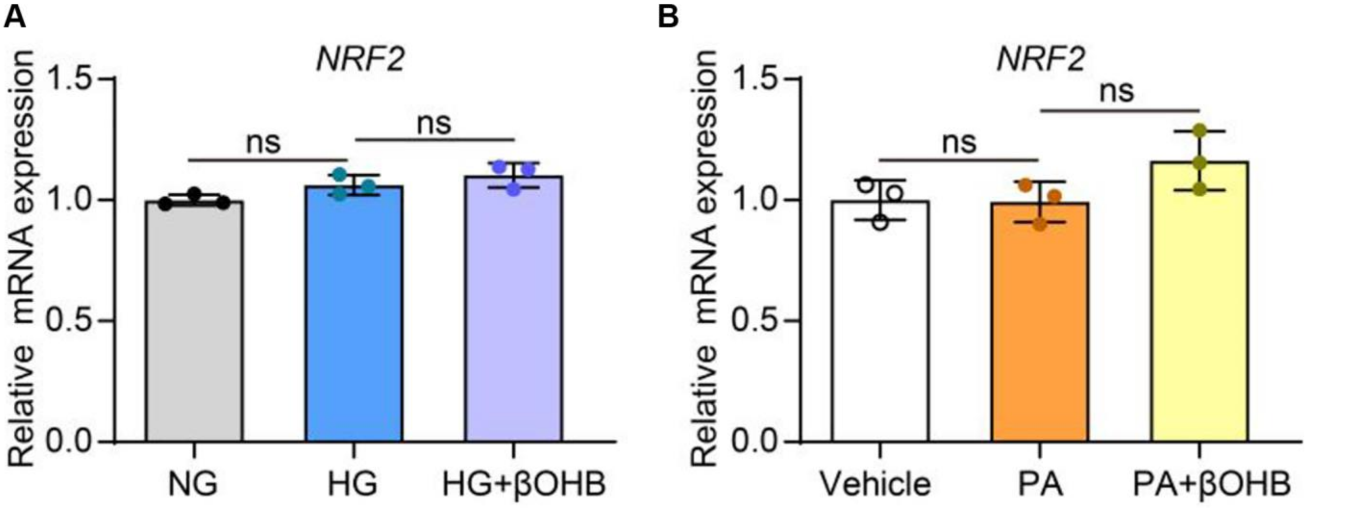

Supplementary Figure 6. qRT-PCR analysis showing the *NRF2* mRNA levels of βOHB-supplemented HK-2 cells treated with HG (**A**) or PA (**B**). All results are representative of three independent experiments. Values are presented as mean ± standard deviation. Abbreviation: ns: no significance.

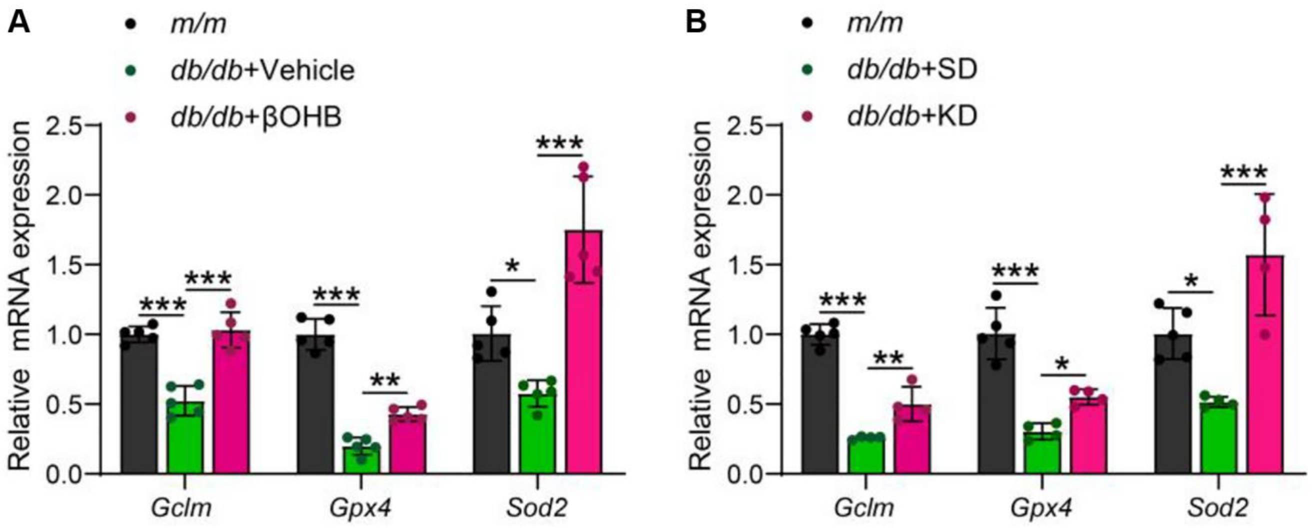

Supplementary Figure 7. qRT-PCR analysis showing the mRNA levels of *Gclm*, *Gpx4*, and *Sod2* in mice kidneys. (**A**) The mRNA levels of *Gclm*, *Gpx4*, and *Sod2* in the kidneys of *m/m* group (n = 5), *db/db* + Vehicle (n = 4), and *db/db* + βOHB group (n = 4). (**B**) The mRNA levels of *Gclm*, *Gpx4*, and *Sod2* in the kidneys of *m/m* group (n = 5), *db/db* + SD (n = 4), and *db/db* + KD group (n = 4). All results are representative of three independent experiments. Values are presented as mean ± standard deviation. Abbreviations: βOHB: β-hydroxybutyrate; SD: standard diet; KD: ketogenic diet. $^{*}P < 0.05$; $^{**}P < 0.01$; $^{***}P < 0.001$.

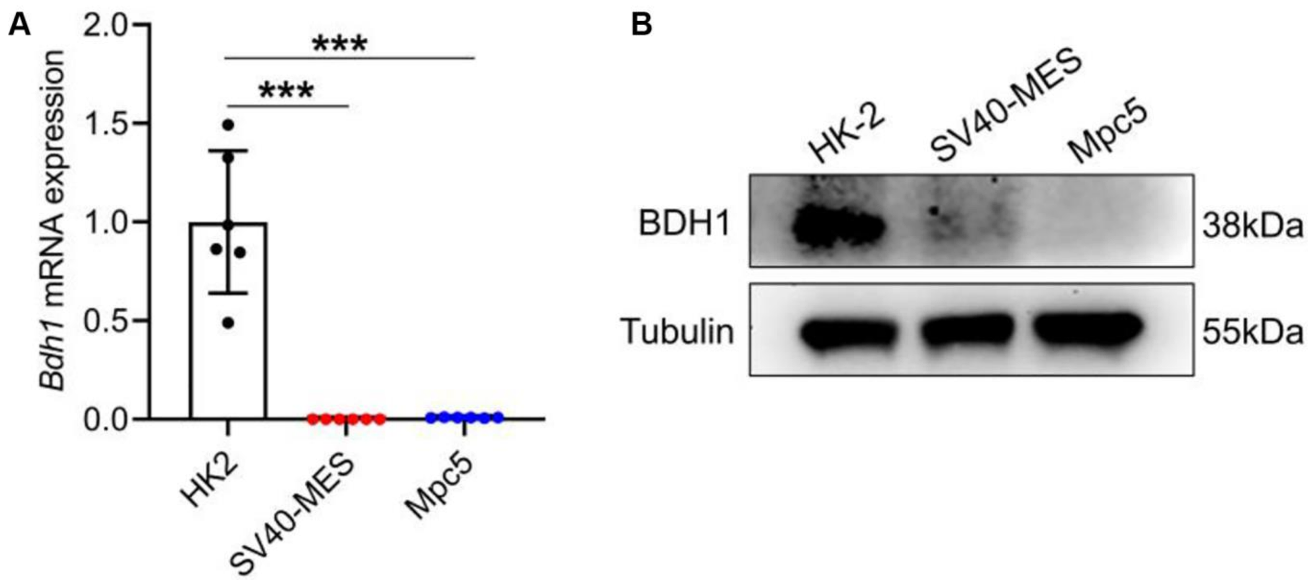

Supplementary Figure 8. (**A**) qRT-PCR analysis showing the mRNA level of *Bdh1* in HK-2 cells, SV40-MES cells, and Mpc5 cells. (**B**) WB analysis showing the protein level of BDH1 in HK-2 cells, SV40-MES cells, and Mpc5 cells. $^{***}P < 0.001$.
