# Supplementary material for: BDH1-mediated βOHB metabolism ameliorates diabetic kidney disease by activation of NRF2-mediated antioxidative pathway
Source: Aging (Albany NY). 2023 Nov 27;15(22):13384–410. doi: 10.18632/aging.205248 (PMC11622694; doi:10.18632/aging.205248)
Supplement: Supplementary Table 1 [file aging-15-205248-s003.docx]

| **Supplementary Table 1. Details on differentially expressed genes.** | | | | | | | | | | | | | | | | |
| --- | --- | --- | --- | --- | --- | --- | --- | --- | --- | --- | --- | --- | --- | --- | --- | --- |
| Gene_id | Gene name | Gene description | FC(db_db/WT) | Log2FC(db_db/WT) | Pvalue | Padjust | Significant | Regulate | WT1 | WT2 | WT3 | db_db1 | db_db2 | db_db3 | WT | db_db |
| ENSMUSG00000099146 | 0610031O16Rik | RIKEN cDNA 0610031O16 gene [Source:MGI Symbol;Acc:MGI:1915619] | 0.095 | -3.398975169 | 2.21E-05 | 0.002254314 | yes | down | 11.18 | 1.48 | 2.22 | 0.07 | 0.42 | 1 | 4.96 | 0.496666667 |
| ENSMUSG00000108207 | 1810059H22Rik | RIKEN cDNA 1810059H22 gene [Source:MGI Symbol;Acc:MGI:1917070] | 0.486 | -1.041714163 | 0.000630501 | 0.032297787 | yes | down | 11.56 | 24.82 | 17.42 | 7.74 | 6.07 | 11.13 | 17.93333333 | 8.313333333 |
| ENSMUSG00000097503 | 3110045C21Rik | RIKEN cDNA 3110045C21 gene [Source:MGI Symbol;Acc:MGI:1914553] | 0.539 | -0.89205543 | 0.000280743 | 0.017406095 | yes | down | 6.21 | 7.25 | 7.43 | 2.88 | 4.03 | 4.77 | 6.963333333 | 3.893333333 |
| ENSMUSG00000053714 | 4732471J01Rik | RIKEN cDNA 4732471J01 gene [Source:MGI Symbol;Acc:MGI:3603586] | 0.453 | -1.141961808 | 0.000315838 | 0.019227187 | yes | down | 1.5 | 1.83 | 2.1 | 0.56 | 0.88 | 1.12 | 1.81 | 0.853333333 |
| ENSMUSG00000109089 | 4833411C07Rik | RIKEN cDNA 4833411C07 gene [Source:MGI Symbol;Acc:MGI:1918874] | 0.162 | -2.626946041 | 0.000276386 | 0.017199404 | yes | down | 1.44 | 0.97 | 0.47 | 0.22 | 0.12 | 0.11 | 0.96 | 0.15 |
| ENSMUSG00000103441 | 7530428D23Rik | RIKEN cDNA 7530428D23 gene [Source:MGI Symbol;Acc:MGI:2442162] | 0.077 | -3.693623954 | 1.55E-06 | 0.000234604 | yes | down | 5.72 | 4.11 | 6.14 | 0.46 | 0.45 | 0.46 | 5.323333333 | 0.456666667 |
| ENSMUSG00000048489 | 8430408G22Rik | RIKEN cDNA 8430408G22 gene [Source:MGI Symbol;Acc:MGI:1918730] | 0.15 | -2.733092971 | 7.89E-09 | 2.37E-06 | yes | down | 16.84 | 13.18 | 4.63 | 2.49 | 1.55 | 1.03 | 11.55 | 1.69 |
| ENSMUSG00000029482 | Aacs | acetoacetyl-CoA synthetase [Source:MGI Symbol;Acc:MGI:1926144] | 0.403 | -1.311671722 | 9.83E-07 | 0.000165093 | yes | down | 29.88 | 28.16 | 16.93 | 7.86 | 14.35 | 7.38 | 24.99 | 9.863333333 |
| ENSMUSG00000015243 | Abca1 | ATP-binding cassette, sub-family A (ABC1), member 1 [Source:MGI Symbol;Acc:MGI:99607] | 1.611 | 0.688087297 | 0.0006949 | 0.034239639 | yes | up | 0.9 | 0.96 | 0.67 | 1.13 | 1.62 | 1.29 | 0.843333333 | 1.346666667 |
| ENSMUSG00000032849 | Abcc4 | ATP-binding cassette, sub-family C (CFTR/MRP), member 4 [Source:MGI Symbol;Acc:MGI:2443111] | 1.664 | 0.734297968 | 0.000992654 | 0.04484717 | yes | up | 4.32 | 5.79 | 3.77 | 5.63 | 11.18 | 6.42 | 4.626666667 | 7.743333333 |
| ENSMUSG00000058835 | Abi1 | abl-interactor 1 [Source:MGI Symbol;Acc:MGI:104913] | 1.834 | 0.875182377 | 0.00108759 | 0.047341158 | yes | up | 20.67 | 18.89 | 19.03 | 29.96 | 32.5 | 20.11 | 19.53 | 27.52333333 |
| ENSMUSG00000111551 | AC115034.1 | - | 0.096 | -3.385665513 | 0.000965153 | 0.044066571 | yes | down | 0.28 | 0.28 | 0.15 | 0 | 0 | 0.06 | 0.236666667 | 0.02 |
| ENSMUSG00000113175 | AC117226.2 | - | 0.051 | -4.284952216 | 3.50E-15 | 3.68E-12 | yes | down | 9.5 | 5.65 | 6.42 | 0.41 | 0.1 | 0.25 | 7.19 | 0.253333333 |
| ENSMUSG00000110980 | AC122428.2 | - | 13.614 | 3.766984675 | 3.54E-05 | 0.003272502 | yes | up | 0.05 | 0.09 | 0 | 0.41 | 0.87 | 0.48 | 0.046666667 | 0.586666667 |
| ENSMUSG00000112057 | AC147512.1 | - | 0.276 | -1.858351471 | 9.39E-06 | 0.00111879 | yes | down | 1.85 | 4.13 | 4.09 | 0.7 | 0.99 | 1.05 | 3.356666667 | 0.913333333 |
| ENSMUSG00000036880 | Acaa2 | acetyl-Coenzyme A acyltransferase 2 (mitochondrial 3-oxoacyl-Coenzyme A thiolase) [Source:MGI Symbol;Acc:MGI:1098623] | 1.557 | 0.63884118 | 0.000102354 | 0.00781705 | yes | up | 105.29 | 145.96 | 110.43 | 189.27 | 186.22 | 172.02 | 120.56 | 182.5033333 |
| ENSMUSG00000032047 | Acat1 | acetyl-Coenzyme A acetyltransferase 1 [Source:MGI Symbol;Acc:MGI:87870] | 0.607 | -0.720574009 | 1.39E-05 | 0.001587694 | yes | down | 348.63 | 314.86 | 310.97 | 196.49 | 205.42 | 183.94 | 324.82 | 195.2833333 |
| ENSMUSG00000062480 | Acat3 | acetyl-Coenzyme A acetyltransferase 3 [Source:MGI Symbol;Acc:MGI:109182] | 1.928 | 0.94691017 | 5.58E-05 | 0.004775412 | yes | up | 7.66 | 6.33 | 7.05 | 9.78 | 19.89 | 12.48 | 7.013333333 | 14.05 |
| ENSMUSG00000020681 | Ace | angiotensin I converting enzyme (peptidyl-dipeptidase A) 1 [Source:MGI Symbol;Acc:MGI:87874] | 0.463 | -1.112009015 | 3.64E-07 | 7.04E-05 | yes | down | 11.67 | 12.5 | 16.87 | 5.29 | 7.95 | 4.87 | 13.68 | 6.036666667 |
| ENSMUSG00000021620 | Acot12 | acyl-CoA thioesterase 12 [Source:MGI Symbol;Acc:MGI:1921406] | 0.617 | -0.695504512 | 0.001021972 | 0.045668018 | yes | down | 43.64 | 47.57 | 47.6 | 32.3 | 29.57 | 21.46 | 46.27 | 27.77666667 |
| ENSMUSG00000076435 | Acsf2 | acyl-CoA synthetase family member 2 [Source:MGI Symbol;Acc:MGI:2388287] | 3.589 | 1.843523259 | 3.91E-05 | 0.003530118 | yes | up | 1.36 | 2.13 | 1.68 | 3.14 | 12.42 | 2.6 | 1.723333333 | 6.053333333 |
| ENSMUSG00000030945 | Acsm2 | acyl-CoA synthetase medium-chain family member 2 [Source:MGI Symbol;Acc:MGI:2385289] | 0.547 | -0.869662347 | 7.30E-05 | 0.005872452 | yes | down | 1356.54 | 1388.4 | 1598.39 | 796.39 | 527.38 | 797.05 | 1447.776667 | 706.94 |
| ENSMUSG00000030935 | Acsm3 | acyl-CoA synthetase medium-chain family member 3 [Source:MGI Symbol;Acc:MGI:99538] | 0.094 | -3.418430531 | 1.03E-07 | 2.27E-05 | yes | down | 143.9 | 52.14 | 113.28 | 15.02 | 8.64 | 8.01 | 103.1066667 | 10.55666667 |
| ENSMUSG00000023262 | Acy1 | aminoacylase 1 [Source:MGI Symbol;Acc:MGI:87913] | 0.621 | -0.688232383 | 0.000172128 | 0.01162481 | yes | down | 55.72 | 50.63 | 53.26 | 27.6 | 31.08 | 39.17 | 53.20333333 | 32.61666667 |
| ENSMUSG00000024866 | Acy3 | aspartoacylase (aminoacylase) 3 [Source:MGI Symbol;Acc:MGI:1918920] | 0.384 | -1.380281839 | 1.50E-07 | 3.23E-05 | yes | down | 456.95 | 533.38 | 843.71 | 247.1 | 184.37 | 251.94 | 611.3466667 | 227.8033333 |
| ENSMUSG00000074207 | Adh1 | alcohol dehydrogenase 1 (class I) [Source:MGI Symbol;Acc:MGI:87921] | 0.406 | -1.299859254 | 7.61E-07 | 0.000136016 | yes | down | 270.46 | 144.6 | 159.86 | 68.31 | 86.95 | 81.5 | 191.64 | 78.92 |
| ENSMUSG00000025911 | Adhfe1 | alcohol dehydrogenase, iron containing, 1 [Source:MGI Symbol;Acc:MGI:1923437] | 0.528 | -0.920677964 | 2.93E-06 | 0.00042075 | yes | down | 34.89 | 37.83 | 48.14 | 19.97 | 18.79 | 23.96 | 40.28666667 | 20.90666667 |
| ENSMUSG00000031980 | Agt | angiotensinogen (serpin peptidase inhibitor, clade A, member 8) [Source:MGI Symbol;Acc:MGI:87963] | 2.082 | 1.058154331 | 7.37E-06 | 0.000952777 | yes | up | 9.59 | 11.34 | 11.78 | 16.33 | 34.99 | 17.63 | 10.90333333 | 22.98333333 |
| ENSMUSG00000105987 | AI506816 | expressed sequence AI506816 [Source:MGI Symbol;Acc:MGI:2140929] | 0.09 | -3.472483817 | 0.000699577 | 0.03436927 | yes | down | 0.63 | 0.9 | 0.04 | 0.02 | 0.02 | 0.14 | 0.523333333 | 0.06 |
| ENSMUSG00000012123 | Aim1l | absent in melanoma 1-like [Source:MGI Symbol;Acc:MGI:1334463] | 3.028 | 1.598244722 | 6.73E-05 | 0.005518286 | yes | up | 1.11 | 0.48 | 0.45 | 1.8 | 1.82 | 1.08 | 0.68 | 1.566666667 |
| ENSMUSG00000033715 | Akr1c14 | aldo-keto reductase family 1, member C14 [Source:MGI Symbol;Acc:MGI:2145458] | 0.205 | -2.288014162 | 8.31E-17 | 1.27E-13 | yes | down | 104.52 | 53.85 | 110.56 | 15.6 | 20.08 | 20 | 89.64333333 | 18.56 |
| ENSMUSG00000021214 | Akr1c18 | aldo-keto reductase family 1, member C18 [Source:MGI Symbol;Acc:MGI:2145420] | 0.043 | -4.54259919 | 7.90E-05 | 0.006257976 | yes | down | 9.89 | 4.4 | 4.6 | 0.62 | 0.08 | 0.04 | 6.296666667 | 0.246666667 |
| ENSMUSG00000071551 | Akr1c19 | aldo-keto reductase family 1, member C19 [Source:MGI Symbol;Acc:MGI:2653678] | 1.623 | 0.698450921 | 0.000886496 | 0.040920082 | yes | up | 11.85 | 12.55 | 9.53 | 12.64 | 23.99 | 22.68 | 11.31 | 19.77 |
| ENSMUSG00000021210 | Akr1c6 | aldo-keto reductase family 1, member C6 [Source:MGI Symbol;Acc:MGI:1933427] | 0.194 | -2.368789462 | 1.52E-05 | 0.001677853 | yes | down | 1.3 | 2.11 | 1.09 | 0.42 | 0.43 | 0.35 | 1.5 | 0.4 |
| ENSMUSG00000053279 | Aldh1a1 | aldehyde dehydrogenase family 1, subfamily A1 [Source:MGI Symbol;Acc:MGI:1353450] | 2.239 | 1.162751594 | 2.08E-05 | 0.002141636 | yes | up | 38.75 | 26.92 | 52.81 | 98.44 | 83.14 | 79.25 | 39.49333333 | 86.94333333 |
| ENSMUSG00000015134 | Aldh1a3 | aldehyde dehydrogenase family 1, subfamily A3 [Source:MGI Symbol;Acc:MGI:1861722] | 0.046 | -4.436052159 | 0.000522896 | 0.028069305 | yes | down | 5.46 | 5.06 | 0.13 | 0.23 | 0.19 | 0.09 | 3.55 | 0.17 |
| ENSMUSG00000024747 | Aldh1a7 | aldehyde dehydrogenase family 1, subfamily A7 [Source:MGI Symbol;Acc:MGI:1347050] | 1.953 | 0.965465433 | 0.000176794 | 0.011881996 | yes | up | 34.41 | 26.8 | 47.61 | 74.24 | 60.06 | 74.21 | 36.27333333 | 69.50333333 |
| ENSMUSG00000028307 | Aldob | aldolase B, fructose-bisphosphate [Source:MGI Symbol;Acc:MGI:87995] | 1.427 | 0.513065881 | 0.000512672 | 0.027697479 | yes | up | 1962.06 | 2344.58 | 2191.47 | 2454.22 | 4052.08 | 2765.29 | 2166.036667 | 3090.53 |
| ENSMUSG00000018924 | Alox15 | arachidonate 15-lipoxygenase [Source:MGI Symbol;Acc:MGI:87997] | 0.091 | -3.465558797 | 2.06E-30 | 1.16E-26 | yes | down | 3.35 | 4.85 | 3.66 | 0.6 | 0.47 | 0.36 | 3.953333333 | 0.476666667 |
| ENSMUSG00000022244 | Amacr | alpha-methylacyl-CoA racemase [Source:MGI Symbol;Acc:MGI:1098273] | 0.562 | -0.830486715 | 9.02E-06 | 0.001082622 | yes | down | 54.67 | 61.81 | 79.74 | 34.05 | 34.81 | 39.52 | 65.40666667 | 36.12666667 |
| ENSMUSG00000028553 | Angptl3 | angiopoietin-like 3 [Source:MGI Symbol;Acc:MGI:1353627] | 3.088 | 1.626644572 | 4.75E-08 | 1.13E-05 | yes | up | 2.74 | 3.22 | 2.92 | 5.05 | 10.28 | 12.85 | 2.96 | 9.393333333 |
| ENSMUSG00000047822 | Angptl8 | angiopoietin-like 8 [Source:MGI Symbol;Acc:MGI:3643534] | 4.998 | 2.321232272 | 5.63E-05 | 0.004775412 | yes | up | 0.42 | 0.49 | 0.33 | 2.25 | 1.96 | 1.81 | 0.413333333 | 2.006666667 |
| ENSMUSG00000029811 | Aoc1 | amine oxidase, copper-containing 1 [Source:MGI Symbol;Acc:MGI:1923757] | 2.282 | 1.190220761 | 0.000334477 | 0.020071019 | yes | up | 0.92 | 0.73 | 0.53 | 1.42 | 2.07 | 1.41 | 0.726666667 | 1.633333333 |
| ENSMUSG00000033096 | Apmap | adipocyte plasma membrane associated protein [Source:MGI Symbol;Acc:MGI:1919131] | 1.378 | 0.462713645 | 0.000344966 | 0.02048099 | yes | up | 28.43 | 36.63 | 30.91 | 36.03 | 53 | 42.46 | 31.99 | 43.83 |
| ENSMUSG00000040564 | Apoc1 | apolipoprotein C-I [Source:MGI Symbol;Acc:MGI:88053] | 6.054 | 2.597797043 | 5.12E-13 | 3.74E-10 | yes | up | 7.25 | 3.36 | 5.66 | 24.97 | 44.26 | 32.16 | 5.423333333 | 33.79666667 |
| ENSMUSG00000032081 | Apoc3 | apolipoprotein C-III [Source:MGI Symbol;Acc:MGI:88055] | 3.215 | 1.684876269 | 2.43E-08 | 6.36E-06 | yes | up | 29.29 | 20.67 | 20.46 | 50.05 | 118.04 | 60.41 | 23.47333333 | 76.16666667 |
| ENSMUSG00000002985 | Apoe | apolipoprotein E [Source:MGI Symbol;Acc:MGI:88057] | 3.106 | 1.635188435 | 4.42E-12 | 2.56E-09 | yes | up | 179.97 | 223.39 | 120.86 | 351.58 | 725.65 | 634.7 | 174.74 | 570.6433333 |
| ENSMUSG00000024411 | Aqp4 | aquaporin 4 [Source:MGI Symbol;Acc:MGI:107387] | 0.473 | -1.0803087 | 1.46E-05 | 0.001638843 | yes | down | 18.96 | 13.95 | 14.34 | 6.25 | 6.14 | 9.84 | 15.75 | 7.41 |
| ENSMUSG00000030762 | Aqp8 | aquaporin 8 [Source:MGI Symbol;Acc:MGI:1195271] | 4.732 | 2.242521442 | 0.000140218 | 0.010025279 | yes | up | 0.16 | 0.24 | 0.37 | 1.02 | 0.79 | 1.43 | 0.256666667 | 1.08 |
| ENSMUSG00000041219 | Arhgap11a | Rho GTPase activating protein 11A [Source:MGI Symbol;Acc:MGI:2444300] | 2.988 | 1.579078094 | 0.000150022 | 0.010546763 | yes | up | 0.28 | 0.21 | 0.24 | 0.68 | 1.09 | 0.4 | 0.243333333 | 0.723333333 |
| ENSMUSG00000030654 | Arl6ip1 | ADP-ribosylation factor-like 6 interacting protein 1 [Source:MGI Symbol;Acc:MGI:1858943] | 0.644 | -0.635171542 | 6.04E-05 | 0.005000305 | yes | down | 246.51 | 238.84 | 229.1 | 136 | 188.55 | 130.99 | 238.15 | 151.8466667 |
| ENSMUSG00000031382 | Asb11 | ankyrin repeat and SOCS box-containing 11 [Source:MGI Symbol;Acc:MGI:1916104] | 7.676 | 2.940408446 | 3.93E-13 | 3.00E-10 | yes | up | 0.62 | 0.35 | 0.69 | 3.38 | 6.45 | 3.11 | 0.553333333 | 4.313333333 |
| ENSMUSG00000037686 | Aspg | asparaginase [Source:MGI Symbol;Acc:MGI:2144822] | 0.602 | -0.731978884 | 2.47E-05 | 0.002468532 | yes | down | 10.37 | 15.21 | 13.87 | 6.33 | 10.67 | 7.11 | 13.15 | 8.036666667 |
| ENSMUSG00000028207 | Asph | aspartate-beta-hydroxylase [Source:MGI Symbol;Acc:MGI:1914186] | 1.505 | 0.589333353 | 1.40E-05 | 0.00159133 | yes | up | 10.98 | 14.77 | 12.49 | 14.86 | 23.67 | 19.22 | 12.74666667 | 19.25 |
| ENSMUSG00000024654 | Asrgl1 | asparaginase like 1 [Source:MGI Symbol;Acc:MGI:1913764] | 1.837 | 0.877193535 | 1.06E-05 | 0.001235848 | yes | up | 2.82 | 2.53 | 1.61 | 2.47 | 6.05 | 2.86 | 2.32 | 3.793333333 |
| ENSMUSG00000076441 | Ass1 | argininosuccinate synthetase 1 [Source:MGI Symbol;Acc:MGI:88090] | 0.669 | -0.580081697 | 4.25E-05 | 0.003795437 | yes | down | 939.51 | 1335.47 | 1101.75 | 710.69 | 760.9 | 731.46 | 1125.576667 | 734.35 |
| ENSMUSG00000018585 | Atox1 | antioxidant 1 copper chaperone [Source:MGI Symbol;Acc:MGI:1333855] | 1.534 | 0.617180917 | 0.001159086 | 0.049681043 | yes | up | 119.11 | 137.88 | 104.9 | 164.73 | 237.75 | 145.73 | 120.63 | 182.7366667 |
| ENSMUSG00000031441 | Atp11a | ATPase, class VI, type 11A [Source:MGI Symbol;Acc:MGI:1354735] | 0.521 | -0.940489042 | 1.96E-06 | 0.000291892 | yes | down | 68.56 | 76.1 | 89.86 | 36.68 | 36.9 | 46.8 | 78.17333333 | 40.12666667 |
| ENSMUSG00000005553 | Atp4a | ATPase, H+/K+ exchanging, gastric, alpha polypeptide [Source:MGI Symbol;Acc:MGI:88113] | 2.075 | 1.053076886 | 2.23E-06 | 0.00032825 | yes | up | 2.16 | 2.68 | 3.49 | 5.76 | 6.12 | 6.65 | 2.776666667 | 6.176666667 |
| ENSMUSG00000006057 | Atp5g1 | ATP synthase, H+ transporting, mitochondrial F0 complex, subunit C1 (subunit 9) [Source:MGI Symbol;Acc:MGI:107653] | 1.431 | 0.516811289 | 1.26E-05 | 0.001457896 | yes | up | 411.01 | 489.53 | 416.14 | 583.54 | 721.23 | 601.89 | 438.8933333 | 635.5533333 |
| ENSMUSG00000022956 | Atp5o | ATP synthase, H+ transporting, mitochondrial F1 complex, O subunit [Source:MGI Symbol;Acc:MGI:106341] | 1.382 | 0.466468235 | 0.000113583 | 0.008429041 | yes | up | 370.4 | 415.79 | 367.81 | 488.89 | 579.83 | 508.18 | 384.6666667 | 525.6333333 |
| ENSMUSG00000054843 | Atrnl1 | attractin like 1 [Source:MGI Symbol;Acc:MGI:2147749] | 0.724 | -0.465068072 | 0.000667386 | 0.033274232 | yes | down | 11.01 | 12.45 | 10.11 | 6.09 | 7.84 | 6.6 | 11.19 | 6.843333333 |
| ENSMUSG00000085998 | AW822252 | expressed sequence AW822252 [Source:MGI Symbol;Acc:MGI:2148030] | 2.875 | 1.523597258 | 0.00077277 | 0.036886571 | yes | up | 8.18 | 4.18 | 10.03 | 7.45 | 36.3 | 18.4 | 7.463333333 | 20.71666667 |
| ENSMUSG00000037053 | Azgp1 | alpha-2-glycoprotein 1, zinc [Source:MGI Symbol;Acc:MGI:103163] | 0.281 | -1.829447637 | 2.61E-13 | 2.09E-10 | yes | down | 180.72 | 173.86 | 155.29 | 51.71 | 32.8 | 54.49 | 169.9566667 | 46.33333333 |
| ENSMUSG00000053706 | B430305J03Rik | RIKEN cDNA B430305J03 gene [Source:MGI Symbol;Acc:MGI:3697707] | 71.528 | 6.160446038 | 4.68E-05 | 0.004098077 | yes | up | 0 | 0 | 0 | 0.14 | 0.06 | 0.23 | 0 | 0.143333333 |
| ENSMUSG00000017929 | B4galt5 | UDP-Gal:betaGlcNAc beta 1,4-galactosyltransferase, polypeptide 5 [Source:MGI Symbol;Acc:MGI:1927169] | 0.483 | -1.049728058 | 7.66E-08 | 1.76E-05 | yes | down | 10.49 | 12.6 | 15.95 | 4.56 | 7.65 | 6.65 | 13.01333333 | 6.286666667 |
| ENSMUSG00000109628 | BC024386 | cDNA sequence BC023486 [Source:MGI Symbol;Acc:MGI:2669313] | 0.534 | -0.905891762 | 0.000166677 | 0.011385945 | yes | down | 31.91 | 43.79 | 40.22 | 18.17 | 17.39 | 26.01 | 38.64 | 20.52333333 |
| ENSMUSG00000030268 | Bcat1 | branched chain aminotransferase 1, cytosolic [Source:MGI Symbol;Acc:MGI:104861] | 0.179 | -2.478046569 | 0.000667204 | 0.033274232 | yes | down | 10.36 | 4.09 | 4.36 | 2.25 | 0.85 | 1.42 | 6.27 | 1.506666667 |
| ENSMUSG00000046598 | Bdh1 | 3-hydroxybutyrate dehydrogenase, type 1 [Source:MGI Symbol;Acc:MGI:1919161] | 0.468 | -1.094595351 | 2.08E-11 | 1.16E-08 | yes | down | 36.91 | 40.21 | 42.09 | 16.68 | 21.3 | 18.09 | 39.73666667 | 18.69 |
| ENSMUSG00000074768 | Bhmt | betaine-homocysteine methyltransferase [Source:MGI Symbol;Acc:MGI:1339972] | 3.529 | 1.819212484 | 2.99E-07 | 6.06E-05 | yes | up | 3.28 | 5.81 | 10.35 | 25 | 16.26 | 24.48 | 6.48 | 21.91333333 |
| ENSMUSG00000058914 | C1qtnf3 | C1q and tumor necrosis factor related protein 3 [Source:MGI Symbol;Acc:MGI:1932136] | 0.421 | -1.247884851 | 1.44E-09 | 5.37E-07 | yes | down | 43 | 43.34 | 65.61 | 17.51 | 22.62 | 23.83 | 50.65 | 21.32 |
| ENSMUSG00000097168 | C230088H06Rik | RIKEN cDNA C230088H06 gene [Source:MGI Symbol;Acc:MGI:2444536] | 0.323 | -1.629523766 | 9.39E-07 | 0.000160991 | yes | down | 7.69 | 12.6 | 5.84 | 4.91 | 2.5 | 3.51 | 8.71 | 3.64 |
| ENSMUSG00000024164 | C3 | complement component 3 [Source:MGI Symbol;Acc:MGI:88227] | 8.265 | 3.047081348 | 0.000287164 | 0.017620074 | yes | up | 6.11 | 5.59 | 2.05 | 10.6 | 93.37 | 18.71 | 4.583333333 | 40.89333333 |
| ENSMUSG00000015451 | C4a | complement component 4A (Rodgers blood group) [Source:MGI Symbol;Acc:MGI:98320] | 0.254 | -1.979123824 | 8.74E-07 | 0.000152007 | yes | down | 1.86 | 1.49 | 1.86 | 0.45 | 0.81 | 1.26 | 1.736666667 | 0.84 |
| ENSMUSG00000035031 | C8a | complement component 8, alpha polypeptide [Source:MGI Symbol;Acc:MGI:2668347] | 0.481 | -1.054682606 | 0.000692587 | 0.034226001 | yes | down | 4.68 | 3.37 | 3.8 | 2.34 | 1.18 | 1.99 | 3.95 | 1.836666667 |
| ENSMUSG00000017978 | Cadps2 | Ca2+-dependent activator protein for secretion 2 [Source:MGI Symbol;Acc:MGI:2443963] | 2.097 | 1.068208297 | 7.21E-05 | 0.005872452 | yes | up | 1.69 | 1.83 | 1.66 | 3.02 | 4.45 | 2.57 | 1.726666667 | 3.346666667 |
| ENSMUSG00000028222 | Calb1 | calbindin 1 [Source:MGI Symbol;Acc:MGI:88248] | 2.233 | 1.159084996 | 6.23E-09 | 1.94E-06 | yes | up | 38.41 | 28.68 | 45.45 | 76.36 | 119.02 | 88.19 | 37.51333333 | 94.52333333 |
| ENSMUSG00000027562 | Car2 | carbonic anhydrase 2 [Source:MGI Symbol;Acc:MGI:88269] | 1.459 | 0.545270366 | 4.60E-06 | 0.00063835 | yes | up | 179.95 | 219.23 | 211.91 | 259.04 | 330.97 | 293.27 | 203.6966667 | 294.4266667 |
| ENSMUSG00000028463 | Car9 | carbonic anhydrase 9 [Source:MGI Symbol;Acc:MGI:2447188] | 0.514 | -0.959197177 | 0.00044614 | 0.024986793 | yes | down | 5.32 | 9.46 | 8.89 | 3.55 | 3 | 5.39 | 7.89 | 3.98 |
| ENSMUSG00000051483 | Cbr1 | carbonyl reductase 1 [Source:MGI Symbol;Acc:MGI:88284] | 1.851 | 0.888163134 | 4.37E-09 | 1.38E-06 | yes | up | 20.23 | 27.83 | 21.49 | 38.98 | 48.33 | 39.23 | 23.18333333 | 42.18 |
| ENSMUSG00000024039 | Cbs | cystathionine beta-synthase [Source:MGI Symbol;Acc:MGI:88285] | 0.518 | -0.949324214 | 6.02E-05 | 0.005000305 | yes | down | 39.5 | 43.25 | 46.64 | 23.04 | 15.71 | 25.32 | 43.13 | 21.35666667 |
| ENSMUSG00000074715 | Ccl28 | chemokine (C-C motif) ligand 28 [Source:MGI Symbol;Acc:MGI:1861731] | 0.228 | -2.135220614 | 9.73E-07 | 0.000165093 | yes | down | 2.43 | 1.43 | 0.96 | 0.41 | 0.47 | 0.22 | 1.606666667 | 0.366666667 |
| ENSMUSG00000070348 | Ccnd1 | cyclin D1 [Source:MGI Symbol;Acc:MGI:88313] | 1.817 | 0.861245929 | 0.000238054 | 0.014869087 | yes | up | 29.64 | 64.74 | 59.1 | 72.3 | 115.69 | 86.49 | 51.16 | 91.49333333 |
| ENSMUSG00000049103 | Ccr2 | chemokine (C-C motif) receptor 2 [Source:MGI Symbol;Acc:MGI:106185] | 0.435 | -1.199734149 | 0.000340646 | 0.020296209 | yes | down | 0.93 | 1.08 | 11.66 | 0.47 | 0.67 | 0.37 | 4.556666667 | 0.503333333 |
| ENSMUSG00000025461 | Cd163l1 | CD163 molecule-like 1 [Source:MGI Symbol;Acc:MGI:2443796] | 11.376 | 3.507902562 | 0.000820127 | 0.038279178 | yes | up | 0.01 | 0.12 | 0.01 | 0.16 | 0.52 | 0.07 | 0.046666667 | 0.25 |
| ENSMUSG00000031494 | Cd209a | CD209a antigen [Source:MGI Symbol;Acc:MGI:2157942] | 0.157 | -2.673343285 | 0.000201751 | 0.013088096 | yes | down | 0.7 | 0.83 | 0.81 | 0.13 | 0.13 | 0.13 | 0.78 | 0.13 |
| ENSMUSG00000002944 | Cd36 | CD36 antigen [Source:MGI Symbol;Acc:MGI:107899] | 0.474 | -1.078253276 | 6.87E-06 | 0.000908283 | yes | down | 125.96 | 78.3 | 95.07 | 30.79 | 58.69 | 51.49 | 99.77666667 | 46.99 |
| ENSMUSG00000037664 | Cdkn1c | cyclin-dependent kinase inhibitor 1C (P57) [Source:MGI Symbol;Acc:MGI:104564] | 1.74 | 0.79943718 | 0.000573099 | 0.029997516 | yes | up | 10.62 | 7.69 | 6.4 | 12.8 | 11.24 | 15.28 | 8.236666667 | 13.10666667 |
| ENSMUSG00000054385 | Ceacam2 | carcinoembryonic antigen-related cell adhesion molecule 2 [Source:MGI Symbol;Acc:MGI:1347246] | 0.285 | -1.811999608 | 2.04E-07 | 4.23E-05 | yes | down | 10.07 | 5.45 | 6.34 | 1.36 | 1.72 | 3.23 | 7.286666667 | 2.103333333 |
| ENSMUSG00000031725 | Ces1f | carboxylesterase 1F [Source:MGI Symbol;Acc:MGI:2142687] | 0.454 | -1.137701242 | 7.24E-06 | 0.000942494 | yes | down | 279.56 | 247.04 | 243.72 | 146.35 | 95.88 | 94.04 | 256.7733333 | 112.09 |
| ENSMUSG00000057074 | Ces1g | carboxylesterase 1G [Source:MGI Symbol;Acc:MGI:88378] | 0.214 | -2.221856318 | 7.84E-06 | 0.000984102 | yes | down | 1.99 | 1.82 | 1.31 | 0.57 | 0.15 | 0.33 | 1.706666667 | 0.35 |
| ENSMUSG00000050097 | Ces2b | carboxyesterase 2B [Source:MGI Symbol;Acc:MGI:2448547] | 0.221 | -2.179850887 | 2.78E-05 | 0.002664937 | yes | down | 0.76 | 0.71 | 2.53 | 0.2 | 0.38 | 0.36 | 1.333333333 | 0.313333333 |
| ENSMUSG00000061825 | Ces2c | carboxylesterase 2C [Source:MGI Symbol;Acc:MGI:2385905] | 0.574 | -0.799702515 | 8.54E-06 | 0.001047343 | yes | down | 89.7 | 97.14 | 110.87 | 52.26 | 71.97 | 45.46 | 99.23666667 | 56.56333333 |
| ENSMUSG00000091813 | Ces2h | carboxylesterase 2H [Source:MGI Symbol;Acc:MGI:3648740] | 0.275 | -1.860946721 | 0.000596526 | 0.03093467 | yes | down | 0.7 | 0.71 | 1.13 | 0.14 | 0.14 | 0.41 | 0.846666667 | 0.23 |
| ENSMUSG00000061780 | Cfd | complement factor D (adipsin) [Source:MGI Symbol;Acc:MGI:87931] | 0.003 | -8.478564231 | 0.000462831 | 0.025749963 | yes | down | 6.18 | 67.33 | 0.24 | 0 | 0 | 0.18 | 24.58333333 | 0.06 |
| ENSMUSG00000040809 | Chil3 | chitinase-like 3 [Source:MGI Symbol;Acc:MGI:1330860] | 52.543 | 5.715425202 | 7.26E-05 | 0.005872452 | yes | up | 0.03 | 0 | 0 | 0.3 | 0.79 | 0.86 | 0.01 | 0.65 |
| ENSMUSG00000031283 | Chrdl1 | chordin-like 1 [Source:MGI Symbol;Acc:MGI:1933172] | 0.107 | -3.220727426 | 1.04E-12 | 6.72E-10 | yes | down | 1.23 | 0.71 | 1.22 | 0.11 | 0.09 | 0.09 | 1.053333333 | 0.096666667 |
| ENSMUSG00000034612 | Chst11 | carbohydrate sulfotransferase 11 [Source:MGI Symbol;Acc:MGI:1927166] | 0.607 | -0.719681962 | 0.000558574 | 0.029513107 | yes | down | 6.34 | 7.66 | 8.12 | 3.16 | 4.34 | 5.83 | 7.373333333 | 4.443333333 |
| ENSMUSG00000030278 | Cidec | cell death-inducing DFFA-like effector c [Source:MGI Symbol;Acc:MGI:95585] | 0.095 | -3.390221193 | 0.000130563 | 0.009455659 | yes | down | 0.51 | 3.77 | 0.29 | 0.14 | 0.12 | 0.26 | 1.523333333 | 0.173333333 |
| ENSMUSG00000032473 | Cldn18 | claudin 18 [Source:MGI Symbol;Acc:MGI:1929209] | 0.019 | -5.702202671 | 0.000505836 | 0.027416303 | yes | down | 0.03 | 0.12 | 0.25 | 0 | 0 | 0 | 0.133333333 | 0 |
| ENSMUSG00000022037 | Clu | clusterin [Source:MGI Symbol;Acc:MGI:88423] | 3.255 | 1.702761144 | 3.68E-12 | 2.21E-09 | yes | up | 26.92 | 33.48 | 31.04 | 74.3 | 157.85 | 68.66 | 30.48 | 100.27 |
| ENSMUSG00000032434 | Cmtm6 | CKLF-like MARVEL transmembrane domain containing 6 [Source:MGI Symbol;Acc:MGI:2447165] | 0.662 | -0.594757027 | 0.000785063 | 0.0372184 | yes | down | 28.52 | 32.09 | 42.47 | 18.73 | 26.24 | 22.95 | 34.36 | 22.64 |
| ENSMUSG00000024644 | Cndp2 | CNDP dipeptidase 2 (metallopeptidase M20 family) [Source:MGI Symbol;Acc:MGI:1913304] | 0.4 | -1.321978281 | 1.64E-15 | 1.84E-12 | yes | down | 183.2 | 285.88 | 282.41 | 88.4 | 111.86 | 94.41 | 250.4966667 | 98.22333333 |
| ENSMUSG00000001755 | Coasy | Coenzyme A synthase [Source:MGI Symbol;Acc:MGI:1918993] | 0.634 | -0.6583094 | 2.40E-06 | 0.000351163 | yes | down | 61.16 | 66 | 60.05 | 36.39 | 43.6 | 36.03 | 62.40333333 | 38.67333333 |
| ENSMUSG00000061518 | Cox5b | cytochrome c oxidase subunit Vb [Source:MGI Symbol;Acc:MGI:88475] | 1.326 | 0.407461206 | 0.001051797 | 0.046383989 | yes | up | 372.26 | 410.84 | 347.9 | 450.45 | 559.74 | 478.35 | 377 | 496.18 |
| ENSMUSG00000037852 | Cpe | carboxypeptidase E [Source:MGI Symbol;Acc:MGI:101932] | 0.191 | -2.389662022 | 2.90E-14 | 2.56E-11 | yes | down | 15.9 | 15.86 | 25.54 | 4.59 | 3.62 | 2.29 | 19.1 | 3.5 |
| ENSMUSG00000113495 | CT573034.3 | - | 0.176 | -2.506314063 | 0.001030637 | 0.045690664 | yes | down | 0.27 | 0.6 | 0.43 | 0.04 | 0.09 | 0.09 | 0.433333333 | 0.073333333 |
| ENSMUSG00000034855 | Cxcl10 | chemokine (C-X-C motif) ligand 10 [Source:MGI Symbol;Acc:MGI:1352450] | 2.962 | 1.566393587 | 2.79E-05 | 0.002664937 | yes | up | 1.46 | 2.12 | 2.04 | 5.68 | 7.48 | 3.07 | 1.873333333 | 5.41 |
| ENSMUSG00000058427 | Cxcl2 | chemokine (C-X-C motif) ligand 2 [Source:MGI Symbol;Acc:MGI:1340094] | 23.626 | 4.562308714 | 0.000182059 | 0.01209074 | yes | up | 0.22 | 0.1 | 0 | 2.22 | 2.43 | 0.05 | 0.106666667 | 1.566666667 |
| ENSMUSG00000029371 | Cxcl5 | chemokine (C-X-C motif) ligand 5 [Source:MGI Symbol;Acc:MGI:1096868] | 35.325 | 5.142598962 | 0.001165541 | 0.049686057 | yes | up | 0.03 | 0 | 0.03 | 0.27 | 2.02 | 0 | 0.02 | 0.763333333 |
| ENSMUSG00000029417 | Cxcl9 | chemokine (C-X-C motif) ligand 9 [Source:MGI Symbol;Acc:MGI:1352449] | 0.108 | -3.215124885 | 7.05E-07 | 0.000128302 | yes | down | 0.58 | 0.73 | 0.44 | 0.3 | 0.09 | 0.02 | 0.583333333 | 0.136666667 |
| ENSMUSG00000046213 | Cym | chymosin [Source:MGI Symbol;Acc:MGI:2684977] | 0.19 | -2.398939352 | 0.000366386 | 0.021301078 | yes | down | 1.06 | 1.16 | 0.94 | 0.04 | 0.28 | 0.29 | 1.053333333 | 0.203333333 |
| ENSMUSG00000074254 | Cyp2a4 | cytochrome P450, family 2, subfamily a, polypeptide 4 [Source:MGI Symbol;Acc:MGI:88596] | 0.428 | -1.224980805 | 8.53E-08 | 1.91E-05 | yes | down | 267.91 | 181.15 | 237.17 | 104.92 | 96.25 | 89.17 | 228.7433333 | 96.78 |
| ENSMUSG00000022445 | Cyp2d26 | cytochrome P450, family 2, subfamily d, polypeptide 26 [Source:MGI Symbol;Acc:MGI:1923529] | 2.035 | 1.024878906 | 4.40E-08 | 1.09E-05 | yes | up | 41.63 | 64.62 | 47.19 | 88.34 | 93.55 | 122.48 | 51.14666667 | 101.4566667 |
| ENSMUSG00000068086 | Cyp2d9 | cytochrome P450, family 2, subfamily d, polypeptide 9 [Source:MGI Symbol;Acc:MGI:88606] | 0.623 | -0.683284905 | 1.86E-05 | 0.001985844 | yes | down | 84.21 | 110.12 | 102.66 | 56.16 | 56.65 | 68.06 | 98.99666667 | 60.29 |
| ENSMUSG00000025479 | Cyp2e1 | cytochrome P450, family 2, subfamily e, polypeptide 1 [Source:MGI Symbol;Acc:MGI:88607] | 0.357 | -1.484929756 | 5.12E-16 | 7.17E-13 | yes | down | 723.12 | 672.59 | 606.25 | 239.78 | 275.26 | 195.62 | 667.32 | 236.8866667 |
| ENSMUSG00000066072 | Cyp4a10 | cytochrome P450, family 4, subfamily a, polypeptide 10 [Source:MGI Symbol;Acc:MGI:88611] | 1.947 | 0.961530813 | 0.000649891 | 0.032889984 | yes | up | 102.01 | 116.82 | 65.73 | 112.79 | 302.51 | 152.32 | 94.85333333 | 189.2066667 |
| ENSMUSG00000066071 | Cyp4a12a | cytochrome P450, family 4, subfamily a, polypeptide 12a [Source:MGI Symbol;Acc:MGI:88612] | 0.322 | -1.634142738 | 4.67E-10 | 2.01E-07 | yes | down | 61.87 | 38.72 | 78.93 | 15.07 | 23.77 | 19.82 | 59.84 | 19.55333333 |
| ENSMUSG00000028715 | Cyp4a14 | cytochrome P450, family 4, subfamily a, polypeptide 14 [Source:MGI Symbol;Acc:MGI:1096550] | 11.416 | 3.513039384 | 1.41E-05 | 0.00159133 | yes | up | 15.36 | 21.95 | 4 | 60.92 | 298.37 | 128.37 | 13.77 | 162.5533333 |
| ENSMUSG00000001467 | Cyp51 | cytochrome P450, family 51 [Source:MGI Symbol;Acc:MGI:106040] | 0.326 | -1.615606075 | 6.19E-21 | 1.73E-17 | yes | down | 32.45 | 31.54 | 38.08 | 8.63 | 13.72 | 11.25 | 34.02333333 | 11.2 |
| ENSMUSG00000039519 | Cyp7b1 | cytochrome P450, family 7, subfamily b, polypeptide 1 [Source:MGI Symbol;Acc:MGI:104978] | 0.095 | -3.392011053 | 1.70E-40 | 1.43E-36 | yes | down | 52.47 | 33.49 | 39.39 | 3.38 | 3.59 | 4.99 | 41.78333333 | 3.986666667 |
| ENSMUSG00000044816 | D630023F18Rik | RIKEN cDNA D630023F18 gene [Source:MGI Symbol;Acc:MGI:2138198] | 2.014 | 1.009909931 | 0.000579167 | 0.03012745 | yes | up | 14.79 | 23.17 | 27.84 | 30.04 | 34.18 | 65.87 | 21.93333333 | 43.36333333 |
| ENSMUSG00000031294 | D630029K05Rik | RIKEN cDNA D630029K05 gene [Source:MGI Symbol;Acc:MGI:2143561] | 0.452 | -1.145231707 | 4.65E-08 | 1.13E-05 | yes | down | 76.93 | 131.56 | 117.97 | 40.9 | 52.22 | 56.9 | 108.82 | 50.00666667 |
| ENSMUSG00000021263 | Degs2 | delta(4)-desaturase, sphingolipid 2 [Source:MGI Symbol;Acc:MGI:1917309] | 0.378 | -1.4025717 | 1.09E-09 | 4.34E-07 | yes | down | 20.75 | 27.14 | 25.5 | 10.61 | 7.29 | 8.42 | 24.46333333 | 8.773333333 |
| ENSMUSG00000025815 | Dhtkd1 | dehydrogenase E1 and transketolase domain containing 1 [Source:MGI Symbol;Acc:MGI:2445096] | 0.643 | -0.637450723 | 0.000460353 | 0.025697155 | yes | down | 3.66 | 6.73 | 5.44 | 2.23 | 5.02 | 5.28 | 5.276666667 | 4.176666667 |
| ENSMUSG00000038060 | Dlec1 | deleted in lung and esophageal cancer 1 [Source:MGI Symbol;Acc:MGI:2443671] | 0.613 | -0.707035506 | 0.000718944 | 0.034912427 | yes | down | 3.75 | 6.64 | 5.24 | 2.38 | 2.91 | 4.12 | 5.21 | 3.136666667 |
| ENSMUSG00000027314 | Dll4 | delta-like 4 (Drosophila) [Source:MGI Symbol;Acc:MGI:1859388] | 0.504 | -0.988814752 | 0.000871306 | 0.0404411 | yes | down | 0.97 | 1.28 | 1.02 | 0.39 | 0.93 | 0.66 | 1.09 | 0.66 |
| ENSMUSG00000026958 | Dpp7 | dipeptidylpeptidase 7 [Source:MGI Symbol;Acc:MGI:1933213] | 0.516 | -0.955181644 | 0.000181791 | 0.01209074 | yes | down | 22.14 | 26.7 | 25.3 | 14.15 | 9.25 | 14.4 | 24.71333333 | 12.6 |
| ENSMUSG00000037474 | Dtl | denticleless E3 ubiquitin protein ligase [Source:MGI Symbol;Acc:MGI:1924093] | 13.601 | 3.765638612 | 0.00036567 | 0.021301078 | yes | up | 0.02 | 0 | 0.02 | 0.1 | 0.35 | 0.11 | 0.013333333 | 0.186666667 |
| ENSMUSG00000082691 | Dynlt1-ps1 | dynein light chain Tctex-type 1, pseuodogene 1 [Source:MGI Symbol;Acc:MGI:3642625] | 73.204 | 6.193845751 | 5.11E-05 | 0.004399001 | yes | up | 0 | 0 | 0 | 7.82 | 2.13 | 6.17 | 0 | 5.373333333 |
| ENSMUSG00000024132 | Eci1 | enoyl-Coenzyme A delta isomerase 1 [Source:MGI Symbol;Acc:MGI:94871] | 1.534 | 0.617514229 | 0.000808344 | 0.038151106 | yes | up | 155.52 | 188.68 | 129.14 | 222.54 | 301.63 | 195.49 | 157.78 | 239.8866667 |
| ENSMUSG00000021416 | Eci3 | enoyl-Coenzyme A delta isomerase 3 [Source:MGI Symbol;Acc:MGI:1916373] | 0.637 | -0.650352444 | 0.00075638 | 0.036207119 | yes | down | 131.06 | 132.61 | 158.82 | 94.84 | 82.33 | 84.08 | 140.83 | 87.08333333 |
| ENSMUSG00000034457 | Eda2r | ectodysplasin A2 receptor [Source:MGI Symbol;Acc:MGI:2442860] | 4.03 | 2.010899916 | 0.000468707 | 0.025905328 | yes | up | 0.05 | 0.18 | 0.09 | 0.35 | 0.22 | 0.47 | 0.106666667 | 0.346666667 |
| ENSMUSG00000028017 | Egf | epidermal growth factor [Source:MGI Symbol;Acc:MGI:95290] | 0.596 | -0.747417513 | 3.40E-06 | 0.000479815 | yes | down | 117.08 | 109.02 | 130.53 | 66.23 | 68.54 | 76.11 | 118.8766667 | 70.29333333 |
| ENSMUSG00000022853 | Ehhadh | enoyl-Coenzyme A, hydratase/3-hydroxyacyl Coenzyme A dehydrogenase [Source:MGI Symbol;Acc:MGI:1277964] | 0.543 | -0.880324489 | 9.60E-09 | 2.69E-06 | yes | down | 97.11 | 111.59 | 108.16 | 54.2 | 68.16 | 47.24 | 105.62 | 56.53333333 |
| ENSMUSG00000021364 | Elovl2 | elongation of very long chain fatty acids (FEN1/Elo2, SUR4/Elo3, yeast)-like 2 [Source:MGI Symbol;Acc:MGI:1858960] | 1.745 | 0.803152262 | 1.66E-07 | 3.53E-05 | yes | up | 3.68 | 3.84 | 3.79 | 5.45 | 7.65 | 6.54 | 3.77 | 6.546666667 |
| ENSMUSG00000063524 | Eno1 | enolase 1, alpha non-neuron [Source:MGI Symbol;Acc:MGI:95393] | 1.642 | 0.715851048 | 0.000627525 | 0.032243647 | yes | up | 163.3 | 198.84 | 188.84 | 221.37 | 453.57 | 239.21 | 183.66 | 304.7166667 |
| ENSMUSG00000030929 | Eri2 | exoribonuclease 2 [Source:MGI Symbol;Acc:MGI:1918401] | 0.613 | -0.705051375 | 0.000979622 | 0.044485435 | yes | down | 2.13 | 2.1 | 2.55 | 1.07 | 1.67 | 1.49 | 2.26 | 1.41 |
| ENSMUSG00000042379 | Esm1 | endothelial cell-specific molecule 1 [Source:MGI Symbol;Acc:MGI:1918940] | 0.237 | -2.077641753 | 9.43E-13 | 6.34E-10 | yes | down | 5.23 | 11.22 | 10.1 | 1.91 | 1.71 | 2.44 | 8.85 | 2.02 |
| ENSMUSG00000029122 | Evc | EvC ciliary complex subunit 1 [Source:MGI Symbol;Acc:MGI:1890596] | 0.638 | -0.64822329 | 0.000217335 | 0.013981043 | yes | down | 50.84 | 64.4 | 54.67 | 35.84 | 31.14 | 36.66 | 56.63666667 | 34.54666667 |
| ENSMUSG00000010663 | Fads1 | fatty acid desaturase 1 [Source:MGI Symbol;Acc:MGI:1923517] | 0.611 | -0.711425951 | 5.03E-05 | 0.004354271 | yes | down | 13.64 | 15.37 | 10.97 | 7.11 | 9.9 | 7.22 | 13.32666667 | 8.076666667 |
| ENSMUSG00000030630 | Fah | fumarylacetoacetate hydrolase [Source:MGI Symbol;Acc:MGI:95482] | 0.693 | -0.528626307 | 7.44E-06 | 0.000954318 | yes | down | 123.96 | 145 | 125.85 | 80.64 | 103.6 | 86.53 | 131.6033333 | 90.25666667 |
| ENSMUSG00000026483 | Fam129a | family with sequence similarity 129, member A [Source:MGI Symbol;Acc:MGI:2137237] | 2.115 | 1.080837267 | 6.06E-06 | 0.000807882 | yes | up | 1.4 | 1.42 | 1.23 | 2.44 | 4.06 | 2.07 | 1.35 | 2.856666667 |
| ENSMUSG00000034871 | Fam151a | family with sequence simliarity 151, member A [Source:MGI Symbol;Acc:MGI:2657115] | 0.447 | -1.162768336 | 3.42E-05 | 0.003191367 | yes | down | 31.19 | 54.46 | 54.17 | 11.9 | 32.42 | 17.02 | 46.60666667 | 20.44666667 |
| ENSMUSG00000029463 | Fam216a | family with sequence similarity 216, member A [Source:MGI Symbol;Acc:MGI:1916198] | 1.503 | 0.587978265 | 0.001079746 | 0.047244492 | yes | up | 7.05 | 8.95 | 7.46 | 11.21 | 12.08 | 11.3 | 7.82 | 11.53 |
| ENSMUSG00000043681 | Fam25c | family with sequence similarity 25, member C [Source:MGI Symbol;Acc:MGI:1916384] | 3.059 | 1.613180996 | 0.00011982 | 0.008791339 | yes | up | 6.05 | 8.93 | 9.31 | 14.46 | 42.86 | 18.22 | 8.096666667 | 25.18 |
| ENSMUSG00000026415 | Fcamr | Fc receptor, IgA, IgM, high affinity [Source:MGI Symbol;Acc:MGI:1927803] | 0.356 | -1.489117326 | 4.71E-08 | 1.13E-05 | yes | down | 10.04 | 14.47 | 13.12 | 3.81 | 3.27 | 6.22 | 12.54333333 | 4.433333333 |
| ENSMUSG00000036585 | Fgf1 | fibroblast growth factor 1 [Source:MGI Symbol;Acc:MGI:95515] | 0.5 | -0.998744368 | 1.89E-05 | 0.001993192 | yes | down | 43.26 | 53.02 | 63.68 | 19.97 | 23.77 | 37.61 | 53.32 | 27.11666667 |
| ENSMUSG00000023092 | Fhl1 | four and a half LIM domains 1 [Source:MGI Symbol;Acc:MGI:1298387] | 0.695 | -0.525151155 | 0.000357989 | 0.021031226 | yes | down | 86.65 | 82.12 | 87.62 | 50.04 | 63.58 | 66.82 | 85.46333333 | 60.14666667 |
| ENSMUSG00000040170 | Fmo2 | flavin containing monooxygenase 2 [Source:MGI Symbol;Acc:MGI:1916776] | 0.635 | -0.654461691 | 0.000106733 | 0.008078071 | yes | down | 125.44 | 141.78 | 114.94 | 82.22 | 81.96 | 72.21 | 127.3866667 | 78.79666667 |
| ENSMUSG00000028088 | Fmo5 | flavin containing monooxygenase 5 [Source:MGI Symbol;Acc:MGI:1310004] | 0.261 | -1.937542794 | 3.83E-08 | 9.60E-06 | yes | down | 16.02 | 14.95 | 10.09 | 4.71 | 1.55 | 3.19 | 13.68666667 | 3.15 |
| ENSMUSG00000001773 | Folh1 | folate hydrolase 1 [Source:MGI Symbol;Acc:MGI:1858193] | 1.439 | 0.524967282 | 0.000331745 | 0.019978413 | yes | up | 47.46 | 61.27 | 48.01 | 70.4 | 72.71 | 82.4 | 52.24666667 | 75.17 |
| ENSMUSG00000024661 | Fth1 | ferritin heavy polypeptide 1 [Source:MGI Symbol;Acc:MGI:95588] | 1.644 | 0.717152537 | 7.10E-07 | 0.000128302 | yes | up | 4907.24 | 5504.14 | 5001.43 | 7143.15 | 10778.88 | 7375.2 | 5137.603333 | 8432.41 |
| ENSMUSG00000029211 | Gabra4 | gamma-aminobutyric acid (GABA) A receptor, subunit alpha 4 [Source:MGI Symbol;Acc:MGI:95616] | 5.135 | 2.360242902 | 0.000647161 | 0.032889984 | yes | up | 0.1 | 0.03 | 0.1 | 0.14 | 0.27 | 0.3 | 0.076666667 | 0.236666667 |
| ENSMUSG00000015027 | Galns | galactosamine (N-acetyl)-6-sulfate sulfatase [Source:MGI Symbol;Acc:MGI:1355303] | 0.638 | -0.648419838 | 0.000172276 | 0.01162481 | yes | down | 15.03 | 17.45 | 18.13 | 10.38 | 13.02 | 8.81 | 16.87 | 10.73666667 |
| ENSMUSG00000035540 | Gc | group specific component [Source:MGI Symbol;Acc:MGI:95669] | 4.7 | 2.232689321 | 6.15E-21 | 1.73E-17 | yes | up | 39.51 | 38.85 | 16.43 | 88.17 | 173.57 | 128.45 | 31.59666667 | 130.0633333 |
| ENSMUSG00000038508 | Gdf15 | growth differentiation factor 15 [Source:MGI Symbol;Acc:MGI:1346047] | 3.88 | 1.955925886 | 7.76E-06 | 0.000984102 | yes | up | 1.69 | 2 | 1.19 | 7.19 | 8.88 | 2.42 | 1.626666667 | 6.163333333 |
| ENSMUSG00000006345 | Ggt1 | gamma-glutamyltransferase 1 [Source:MGI Symbol;Acc:MGI:95706] | 0.659 | -0.601883756 | 0.000925096 | 0.042584814 | yes | down | 301.34 | 380.84 | 376.26 | 238.72 | 200.29 | 248.49 | 352.8133333 | 229.1666667 |
| ENSMUSG00000055737 | Ghr | growth hormone receptor [Source:MGI Symbol;Acc:MGI:95708] | 0.701 | -0.51338877 | 0.000885445 | 0.040920082 | yes | down | 230.59 | 230.16 | 225.66 | 154.29 | 145.9 | 151.7 | 228.8033333 | 150.63 |
| ENSMUSG00000021591 | Glrx | glutaredoxin [Source:MGI Symbol;Acc:MGI:2135625] | 1.375 | 0.459876464 | 0.001076047 | 0.047205571 | yes | up | 53.63 | 53.09 | 50.55 | 65.17 | 84.78 | 69.14 | 52.42333333 | 73.03 |
| ENSMUSG00000058360 | Gm10040 | predicted gene 10040 [Source:MGI Symbol;Acc:MGI:3708676] | 0.258 | -1.955355392 | 0.000128172 | 0.0093412 | yes | down | 1.83 | 2.2 | 0.82 | 0.23 | 0.44 | 0.59 | 1.616666667 | 0.42 |
| ENSMUSG00000074179 | Gm10639 | predicted gene 10639 [Source:MGI Symbol;Acc:MGI:3704339] | 7.8 | 2.963514822 | 2.07E-05 | 0.002141636 | yes | up | 1.8 | 5.28 | 2.68 | 7.73 | 23.87 | 41.18 | 3.253333333 | 24.26 |
| ENSMUSG00000083294 | Gm14957 | predicted gene 14957 [Source:MGI Symbol;Acc:MGI:3705824] | 0.153 | -2.708296921 | 2.62E-11 | 1.42E-08 | yes | down | 1.51 | 1.45 | 3.28 | 0.33 | 0.22 | 0.39 | 2.08 | 0.313333333 |
| ENSMUSG00000081143 | Gm15823 | predicted gene 15823 [Source:MGI Symbol;Acc:MGI:3801858] | 0.505 | -0.985532364 | 0.000820154 | 0.038279178 | yes | down | 3.44 | 4.47 | 5.08 | 1.38 | 2.28 | 2.94 | 4.33 | 2.2 |
| ENSMUSG00000086706 | Gm15848 | predicted gene 15848 [Source:MGI Symbol;Acc:MGI:3802054] | 0.394 | -1.345246384 | 4.09E-09 | 1.32E-06 | yes | down | 9.1 | 15.22 | 14.51 | 4.89 | 4.51 | 6.46 | 12.94333333 | 5.286666667 |
| ENSMUSG00000072905 | Gm2016 | predicted gene 2016 [Source:MGI Symbol;Acc:MGI:3780185] | 0.002 | -8.825552264 | 1.10E-06 | 0.000179272 | yes | down | 4.6 | 0.91 | 0.85 | 0 | 0 | 0 | 2.12 | 0 |
| ENSMUSG00000078886 | Gm2026 | predicted gene 2026 [Source:MGI Symbol;Acc:MGI:3780195] | 3.865 | 1.950637806 | 0.001026465 | 0.045690664 | yes | up | 0.12 | 0.3 | 0.84 | 1.35 | 1.93 | 1.35 | 0.42 | 1.543333333 |
| ENSMUSG00000070392 | Gm20634 | predicted gene 20634 [Source:MGI Symbol;Acc:MGI:5313081] | 0.26 | -1.945335485 | 3.64E-11 | 1.91E-08 | yes | down | 2.22 | 3.25 | 2.16 | 0.49 | 0.59 | 0.87 | 2.543333333 | 0.65 |
| ENSMUSG00000096878 | Gm21083 | predicted gene, 21083 [Source:MGI Symbol;Acc:MGI:5434438] | 16.075 | 4.006713143 | 0.000142579 | 0.010150903 | yes | up | 0.04 | 0 | 0.14 | 1.39 | 0.43 | 0.91 | 0.06 | 0.91 |
| ENSMUSG00000099762 | Gm21149 | predicted gene, 21149 [Source:MGI Symbol;Acc:MGI:5434504] | 6.726 | 2.749670415 | 0.000195631 | 0.012891989 | yes | up | 0.04 | 0.49 | 0.33 | 2.94 | 1.57 | 0.92 | 0.286666667 | 1.81 |
| ENSMUSG00000094230 | Gm21847 | predicted gene, 21847 [Source:MGI Symbol;Acc:MGI:5434011] | 7.278 | 2.863536736 | 7.16E-06 | 0.000939968 | yes | up | 0.21 | 0.42 | 0.71 | 3.25 | 3 | 3.27 | 0.446666667 | 3.173333333 |
| ENSMUSG00000096764 | Gm21985 | predicted gene 21985 [Source:MGI Symbol;Acc:MGI:5439454] | 0.019 | -5.700211224 | 0.000223768 | 0.014137678 | yes | down | 0.11 | 0.09 | 0.07 | 0 | 0 | 0 | 0.09 | 0 |
| ENSMUSG00000092822 | Gm23162 | predicted gene, 23162 [Source:MGI Symbol;Acc:MGI:5452939] | 0.003 | -8.631314584 | 4.72E-22 | 1.98E-18 | yes | down | 55.64 | 30.05 | 48.31 | 0 | 0 | 0.35 | 44.66666667 | 0.116666667 |
| ENSMUSG00000098650 | Gm28048 | predicted gene, 28048 [Source:MGI Symbol;Acc:MGI:5547784] | 1.504 | 0.589190379 | 0.000359594 | 0.021051897 | yes | up | 7.1 | 7.1 | 7.9 | 10.41 | 12.37 | 10.18 | 7.366666667 | 10.98666667 |
| ENSMUSG00000099669 | Gm29012 | predicted gene 29012 [Source:MGI Symbol;Acc:MGI:5579718] | 0.32 | -1.645316982 | 2.46E-08 | 6.36E-06 | yes | down | 9.76 | 14.25 | 12.44 | 2.8 | 5.25 | 4.5 | 12.15 | 4.183333333 |
| ENSMUSG00000100750 | Gm29084 | predicted gene 29084 [Source:MGI Symbol;Acc:MGI:5579790] | 0.193 | -2.369665771 | 1.22E-10 | 5.85E-08 | yes | down | 8.01 | 11.95 | 7.61 | 1.36 | 0.96 | 2.88 | 9.19 | 1.733333333 |
| ENSMUSG00000111709 | Gm3776 | predicted gene 3776 [Source:NCBI gene;Acc:100042295] | 19.308 | 4.271092396 | 1.76E-07 | 3.70E-05 | yes | up | 0.47 | 0.31 | 0.1 | 0.51 | 10.11 | 7.68 | 0.293333333 | 6.1 |
| ENSMUSG00000103949 | Gm38226 | predicted gene, 38226 [Source:MGI Symbol;Acc:MGI:5611454] | 0.121 | -3.048786098 | 0.000440992 | 0.024864253 | yes | down | 3.48 | 1.98 | 4.02 | 0 | 0.74 | 0.52 | 3.16 | 0.42 |
| ENSMUSG00000100426 | Gm4208 | predicted gene 4208 [Source:MGI Symbol;Acc:MGI:3782384] | 0.45 | -1.150590304 | 0.000488088 | 0.026800181 | yes | down | 4.87 | 8.64 | 6.15 | 2.45 | 2.18 | 3.97 | 6.553333333 | 2.866666667 |
| ENSMUSG00000105373 | Gm42429 | predicted gene 42429 [Source:MGI Symbol;Acc:MGI:5662566] | 0.184 | -2.44321851 | 0.000221849 | 0.014119334 | yes | down | 5.8 | 1.99 | 6.15 | 0.65 | 0.79 | 1.2 | 4.646666667 | 0.88 |
| ENSMUSG00000106044 | Gm42860 | predicted gene 42860 [Source:MGI Symbol;Acc:MGI:5662997] | 0.578 | -0.791988602 | 0.00017162 | 0.01162481 | yes | down | 5.36 | 8 | 5.46 | 2.49 | 4.88 | 3.48 | 6.273333333 | 3.616666667 |
| ENSMUSG00000109291 | Gm45043 | predicted gene 45043 [Source:MGI Symbol;Acc:MGI:5753619] | 0.082 | -3.613588946 | 0.000637584 | 0.03256134 | yes | down | 0.87 | 0.5 | 0.47 | 0.04 | 0.11 | 0 | 0.613333333 | 0.05 |
| ENSMUSG00000110631 | Gm45836 | predicted gene 45836 [Source:MGI Symbol;Acc:MGI:5804951] | 2.883 | 1.527546269 | 0.000339459 | 0.020296209 | yes | up | 0.35 | 0.53 | 1.1 | 0.83 | 1.76 | 2.25 | 0.66 | 1.613333333 |
| ENSMUSG00000110277 | Gm45871 | predicted gene 45871 [Source:MGI Symbol;Acc:MGI:5804986] | 0.431 | -1.213021927 | 8.51E-08 | 1.91E-05 | yes | down | 4.88 | 5.07 | 5.71 | 2.08 | 3.07 | 2.26 | 5.22 | 2.47 |
| ENSMUSG00000025936 | Gm4956 | predicted gene 4956 [Source:MGI Symbol;Acc:MGI:3647976] | 0.386 | -1.374144828 | 3.65E-05 | 0.003341456 | yes | down | 11.59 | 8.55 | 8.27 | 4.36 | 2.84 | 2.78 | 9.47 | 3.326666667 |
| ENSMUSG00000078706 | Gm53 | predicted gene 53 [Source:MGI Symbol;Acc:MGI:2684899] | 1.428 | 0.514216389 | 0.001117103 | 0.048127096 | yes | up | 6.57 | 6.57 | 6.69 | 9.06 | 13.14 | 9.11 | 6.61 | 10.43666667 |
| ENSMUSG00000079029 | Gm5662 | predicted gene 5662 [Source:MGI Symbol;Acc:MGI:3648257] | 0.012 | -6.366127515 | 3.28E-07 | 6.56E-05 | yes | down | 2.9 | 0.42 | 0.89 | 0.02 | 0 | 0.03 | 1.403333333 | 0.016666667 |
| ENSMUSG00000106069 | Gm6135 | prediticted gene 6135 [Source:MGI Symbol;Acc:MGI:3643755] | 2.954 | 1.56258155 | 0.000563564 | 0.029615964 | yes | up | 7 | 8.15 | 6.94 | 11.67 | 18.72 | 35.52 | 7.363333333 | 21.97 |
| ENSMUSG00000104375 | Gm6300 | predicted gene 6300 [Source:MGI Symbol;Acc:MGI:3648364] | 0.002 | -9.261345304 | 8.31E-09 | 2.45E-06 | yes | down | 3.53 | 0.97 | 5.34 | 0 | 0 | 0 | 3.28 | 0 |
| ENSMUSG00000079263 | Gm6614 | predicted gene 6614 [Source:MGI Symbol;Acc:MGI:3647159] | 4.411 | 2.140965277 | 1.61E-12 | 1.00E-09 | yes | up | 1.29 | 0.61 | 0.58 | 3.44 | 4.04 | 3.04 | 0.826666667 | 3.506666667 |
| ENSMUSG00000090713 | Gm8127 | predicted gene 8127 [Source:MGI Symbol;Acc:MGI:3646865] | 8.61 | 3.106015233 | 4.92E-06 | 0.000677735 | yes | up | 1.07 | 0.84 | 0.52 | 4.02 | 17.7 | 2.56 | 0.81 | 8.093333333 |
| ENSMUSG00000023120 | Gm853 | predicted gene 853 [Source:MGI Symbol;Acc:MGI:2685699] | 0.199 | -2.329482285 | 2.84E-09 | 9.85E-07 | yes | down | 9.07 | 13.02 | 22.15 | 1.81 | 1.88 | 5.34 | 14.74666667 | 3.01 |
| ENSMUSG00000045455 | Gm9797 | predicted pseudogene 9797 [Source:MGI Symbol;Acc:MGI:3704349] | 1.944 | 0.959019987 | 9.65E-05 | 0.007472112 | yes | up | 11.92 | 11.64 | 15.43 | 23.62 | 20.39 | 26.59 | 12.99666667 | 23.53333333 |
| ENSMUSG00000023019 | Gpd1 | glycerol-3-phosphate dehydrogenase 1 (soluble) [Source:MGI Symbol;Acc:MGI:95679] | 1.647 | 0.719931389 | 1.00E-06 | 0.000166643 | yes | up | 74.8 | 109.89 | 106.04 | 137.97 | 180.19 | 152.83 | 96.91 | 156.9966667 |
| ENSMUSG00000048216 | Gpr85 | G protein-coupled receptor 85 [Source:MGI Symbol;Acc:MGI:1927851] | 3.949 | 1.981466273 | 8.03E-06 | 0.000998943 | yes | up | 0.21 | 0.26 | 0.25 | 0.54 | 0.84 | 0.94 | 0.24 | 0.773333333 |
| ENSMUSG00000045441 | Gprin3 | GPRIN family member 3 [Source:MGI Symbol;Acc:MGI:1924785] | 3.433 | 1.779289466 | 3.46E-07 | 6.76E-05 | yes | up | 0.18 | 0.22 | 0.31 | 0.47 | 0.85 | 1.15 | 0.236666667 | 0.823333333 |
| ENSMUSG00000074934 | Grem1 | gremlin 1, DAN family BMP antagonist [Source:MGI Symbol;Acc:MGI:1344337] | 19.26 | 4.267520864 | 5.68E-13 | 3.98E-10 | yes | up | 0.07 | 0.01 | 0.04 | 0.63 | 1.17 | 0.58 | 0.04 | 0.793333333 |
| ENSMUSG00000050069 | Grem2 | gremlin 2, DAN family BMP antagonist [Source:MGI Symbol;Acc:MGI:1344367] | 5.32 | 2.411334271 | 1.05E-15 | 1.36E-12 | yes | up | 0.42 | 0.47 | 0.52 | 2.26 | 3.45 | 1.74 | 0.47 | 2.483333333 |
| ENSMUSG00000001986 | Gria3 | glutamate receptor, ionotropic, AMPA3 (alpha 3) [Source:MGI Symbol;Acc:MGI:95810] | 2.657 | 1.409615163 | 2.66E-06 | 0.000384879 | yes | up | 0.59 | 0.85 | 0.92 | 1.29 | 2.1 | 2.79 | 0.786666667 | 2.06 |
| ENSMUSG00000050107 | Gsg2 | germ cell associated 2, haspin [Source:MGI Symbol;Acc:MGI:1194498] | 3.12 | 1.641612165 | 0.000230005 | 0.014419969 | yes | up | 0.28 | 0.18 | 0.2 | 0.63 | 0.72 | 0.72 | 0.22 | 0.69 |
| ENSMUSG00000074183 | Gsta1 | glutathione S-transferase, alpha 1 (Ya) [Source:MGI Symbol;Acc:MGI:1095417] | 13.91 | 3.798033588 | 1.00E-18 | 2.11E-15 | yes | up | 2.13 | 2 | 0.69 | 12.05 | 40.62 | 19.49 | 1.606666667 | 24.05333333 |
| ENSMUSG00000025934 | Gsta3 | glutathione S-transferase, alpha 3 [Source:MGI Symbol;Acc:MGI:95856] | 1.448 | 0.534131352 | 0.001162176 | 0.049686057 | yes | up | 113.8 | 121.54 | 88.21 | 133.79 | 179.65 | 146.29 | 107.85 | 153.2433333 |
| ENSMUSG00000004032 | Gstm5 | glutathione S-transferase, mu 5 [Source:MGI Symbol;Acc:MGI:1309466] | 1.539 | 0.621775987 | 0.000995595 | 0.04484717 | yes | up | 229.56 | 253.86 | 170.4 | 274.66 | 456.87 | 310.35 | 217.94 | 347.2933333 |
| ENSMUSG00000025068 | Gsto1 | glutathione S-transferase omega 1 [Source:MGI Symbol;Acc:MGI:1342273] | 2.162 | 1.112437211 | 9.07E-09 | 2.58E-06 | yes | up | 24.42 | 30.57 | 22.48 | 58.32 | 57.98 | 45.76 | 25.82333333 | 54.02 |
| ENSMUSG00000058216 | Gstp3 | glutathione S-transferase pi 3 [Source:MGI Symbol;Acc:MGI:2385078] | 6.763 | 2.7577006 | 0.001086663 | 0.047341158 | yes | up | 0.68 | 0.1 | 0.18 | 1.13 | 4.08 | 1.53 | 0.32 | 2.246666667 |
| ENSMUSG00000032978 | Guca2b | guanylate cyclase activator 2b (retina) [Source:MGI Symbol;Acc:MGI:1270851] | 1.601 | 0.678959079 | 2.25E-05 | 0.002282199 | yes | up | 404.84 | 464.48 | 418.25 | 593.04 | 633.82 | 815.98 | 429.19 | 680.9466667 |
| ENSMUSG00000028497 | Hacd4 | 3-hydroxyacyl-CoA dehydratase 4 [Source:MGI Symbol;Acc:MGI:1914025] | 0.365 | -1.453428878 | 4.68E-05 | 0.004098077 | yes | down | 3.23 | 2.52 | 1.45 | 0.79 | 0.71 | 1 | 2.4 | 0.833333333 |
| ENSMUSG00000024486 | Hbegf | heparin-binding EGF-like growth factor [Source:MGI Symbol;Acc:MGI:96070] | 0.572 | -0.805600185 | 0.00031162 | 0.019039393 | yes | down | 4.36 | 5.73 | 3.95 | 2.53 | 3.16 | 2.2 | 4.68 | 2.63 |
| ENSMUSG00000027360 | Hdc | histidine decarboxylase [Source:MGI Symbol;Acc:MGI:96062] | 2.572 | 1.363140877 | 0.000111621 | 0.008335372 | yes | up | 0.44 | 0.69 | 0.73 | 1.06 | 1.95 | 1.53 | 0.62 | 1.513333333 |
| ENSMUSG00000006611 | Hfe | hemochromatosis [Source:MGI Symbol;Acc:MGI:109191] | 1.592 | 0.670523104 | 0.000522182 | 0.028069305 | yes | up | 6.41 | 9.16 | 6.81 | 8.55 | 15.45 | 11.53 | 7.46 | 11.84333333 |
| ENSMUSG00000036181 | Hist1h1c | histone cluster 1, H1c [Source:MGI Symbol;Acc:MGI:1931526] | 0.593 | -0.753028557 | 0.000148074 | 0.010453494 | yes | down | 50.53 | 53.35 | 45.16 | 26.74 | 24.66 | 35.36 | 49.68 | 28.92 |
| ENSMUSG00000064220 | Hist2h2aa1 | histone cluster 2, H2aa1 [Source:MGI Symbol;Acc:MGI:96097] | 0.429 | -1.219681184 | 9.76E-10 | 4.10E-07 | yes | down | 79.2 | 83.68 | 78.13 | 32.07 | 29.42 | 40.52 | 80.33666667 | 34.00333333 |
| ENSMUSG00000068854 | Hist2h2be | histone cluster 2, H2be [Source:MGI Symbol;Acc:MGI:2448415] | 0.449 | -1.155732213 | 0.00010597 | 0.008056581 | yes | down | 2.86 | 2.94 | 1.73 | 0.98 | 1.1 | 1.26 | 2.51 | 1.113333333 |
| ENSMUSG00000021670 | Hmgcr | 3-hydroxy-3-methylglutaryl-Coenzyme A reductase [Source:MGI Symbol;Acc:MGI:96159] | 0.436 | -1.197659116 | 3.36E-07 | 6.64E-05 | yes | down | 6.58 | 4.42 | 4.4 | 1.85 | 2.72 | 2.78 | 5.133333333 | 2.45 |
| ENSMUSG00000093930 | Hmgcs1 | 3-hydroxy-3-methylglutaryl-Coenzyme A synthase 1 [Source:MGI Symbol;Acc:MGI:107592] | 0.565 | -0.824052501 | 8.21E-05 | 0.006474777 | yes | down | 75.15 | 81.09 | 58.6 | 32.99 | 57.49 | 31.67 | 71.61333333 | 40.71666667 |
| ENSMUSG00000028572 | Hook1 | hook microtubule tethering protein 1 [Source:MGI Symbol;Acc:MGI:1925213] | 1.529 | 0.612618835 | 0.000749902 | 0.035999594 | yes | up | 11.67 | 11.61 | 13.58 | 13.83 | 15.88 | 18.89 | 12.28666667 | 16.2 |
| ENSMUSG00000022877 | Hrg | histidine-rich glycoprotein [Source:MGI Symbol;Acc:MGI:2146636] | 0.148 | -2.757820683 | 5.68E-05 | 0.004775412 | yes | down | 0.55 | 1.06 | 1.54 | 0.21 | 0.21 | 0.03 | 1.05 | 0.15 |
| ENSMUSG00000029311 | Hsd17b11 | hydroxysteroid (17-beta) dehydrogenase 11 [Source:MGI Symbol;Acc:MGI:2149821] | 0.452 | -1.146767877 | 2.13E-14 | 1.99E-11 | yes | down | 109.92 | 109.77 | 113.58 | 48.82 | 51.15 | 48.31 | 111.09 | 49.42666667 |
| ENSMUSG00000031844 | Hsd17b2 | hydroxysteroid (17-beta) dehydrogenase 2 [Source:MGI Symbol;Acc:MGI:1096386] | 0.451 | -1.149593797 | 0.000539131 | 0.028666065 | yes | down | 5.69 | 5.59 | 7.31 | 1.32 | 4.08 | 3.15 | 6.196666667 | 2.85 |
| ENSMUSG00000095143 | Hsd3b4 | hydroxy-delta-5-steroid dehydrogenase, 3 beta- and steroid delta-isomerase 4 [Source:MGI Symbol;Acc:MGI:96236] | 0.418 | -1.258673731 | 2.53E-05 | 0.002497423 | yes | down | 81.67 | 43 | 39.59 | 21.66 | 19.95 | 25.2 | 54.75333333 | 22.27 |
| ENSMUSG00000049511 | Htr1b | 5-hydroxytryptamine (serotonin) receptor 1B [Source:MGI Symbol;Acc:MGI:96274] | 2.419 | 1.274707491 | 0.000410416 | 0.023375607 | yes | up | 0.34 | 0.34 | 0.32 | 0.99 | 0.67 | 0.69 | 0.333333333 | 0.783333333 |
| ENSMUSG00000058258 | Idi1 | isopentenyl-diphosphate delta isomerase [Source:MGI Symbol;Acc:MGI:2442264] | 0.374 | -1.417203597 | 4.80E-07 | 8.97E-05 | yes | down | 21.99 | 21.94 | 42.84 | 8.79 | 9.83 | 14.05 | 28.92333333 | 10.89 |
| ENSMUSG00000031549 | Ido2 | indoleamine 2,3-dioxygenase 2 [Source:MGI Symbol;Acc:MGI:2142489] | 0.347 | -1.526864776 | 2.06E-08 | 5.49E-06 | yes | down | 4.92 | 5.34 | 4.97 | 1.19 | 1.32 | 3.53 | 5.076666667 | 2.013333333 |
| ENSMUSG00000003541 | Ier3 | immediate early response 3 [Source:MGI Symbol;Acc:MGI:104814] | 1.922 | 0.942759112 | 0.000395281 | 0.022667298 | yes | up | 11.96 | 12.73 | 7.96 | 23.3 | 22.8 | 15.26 | 10.88333333 | 20.45333333 |
| ENSMUSG00000066677 | Ifi208 | interferon activated gene 208 [Source:MGI Symbol;Acc:MGI:2442822] | 0.266 | -1.911539959 | 0.000616065 | 0.031849597 | yes | down | 0.56 | 0.34 | 0.49 | 0.1 | 0.14 | 0.12 | 0.463333333 | 0.12 |
| ENSMUSG00000028037 | Ifi44 | interferon-induced protein 44 [Source:MGI Symbol;Acc:MGI:2443016] | 0.533 | -0.907585669 | 0.000152855 | 0.010656755 | yes | down | 4.23 | 2.96 | 3.89 | 1.81 | 2.48 | 1.84 | 3.693333333 | 2.043333333 |
| ENSMUSG00000020427 | Igfbp3 | insulin-like growth factor binding protein 3 [Source:MGI Symbol;Acc:MGI:96438] | 0.568 | -0.817210372 | 3.98E-05 | 0.003580165 | yes | down | 41.47 | 51.9 | 35.58 | 24.2 | 28.9 | 18.59 | 42.98333333 | 23.89666667 |
| ENSMUSG00000003477 | Inmt | indolethylamine N-methyltransferase [Source:MGI Symbol;Acc:MGI:102963] | 0.41 | -1.284580441 | 6.43E-07 | 0.00011878 | yes | down | 1101.83 | 1208.98 | 1013.75 | 576.66 | 341.73 | 388.07 | 1108.186667 | 435.4866667 |
| ENSMUSG00000021676 | Iqgap2 | IQ motif containing GTPase activating protein 2 [Source:MGI Symbol;Acc:MGI:2449975] | 1.635 | 0.70955659 | 0.000147511 | 0.010453494 | yes | up | 3.91 | 4.06 | 4.98 | 5 | 8.61 | 7.68 | 4.316666667 | 7.096666667 |
| ENSMUSG00000037254 | Itih2 | inter-alpha trypsin inhibitor, heavy chain 2 [Source:MGI Symbol;Acc:MGI:96619] | 0.359 | -1.476199826 | 1.73E-05 | 0.001881652 | yes | down | 1.58 | 1.23 | 1.89 | 0.59 | 1.71 | 0.43 | 1.566666667 | 0.91 |
| ENSMUSG00000027332 | Ivd | isovaleryl coenzyme A dehydrogenase [Source:MGI Symbol;Acc:MGI:1929242] | 0.5 | -1.00120941 | 2.66E-10 | 1.18E-07 | yes | down | 82.57 | 86.35 | 86.08 | 39.95 | 39.76 | 44.68 | 85 | 41.46333333 |
| ENSMUSG00000019762 | Iyd | iodotyrosine deiodinase [Source:MGI Symbol;Acc:MGI:1917587] | 1.551 | 0.63303617 | 1.39E-06 | 0.000216986 | yes | up | 40.47 | 44.68 | 43.09 | 57.33 | 74.88 | 71.85 | 42.74666667 | 68.02 |
| ENSMUSG00000032758 | Kap | kidney androgen regulated protein [Source:MGI Symbol;Acc:MGI:96653] | 0.195 | -2.358656115 | 7.74E-05 | 0.006165057 | yes | down | 51438.95 | 42568.19 | 58554.1 | 15377.33 | 6098.81 | 6722.96 | 50853.74667 | 9399.7 |
| ENSMUSG00000055675 | Kbtbd11 | kelch repeat and BTB (POZ) domain containing 11 [Source:MGI Symbol;Acc:MGI:1922151] | 0.601 | -0.733833581 | 1.99E-05 | 0.002072223 | yes | down | 5.74 | 4.58 | 10.26 | 2.66 | 5.07 | 4.97 | 6.86 | 4.233333333 |
| ENSMUSG00000074575 | Kcng1 | potassium voltage-gated channel, subfamily G, member 1 [Source:MGI Symbol;Acc:MGI:3616086] | 0.032 | -4.94597239 | 7.30E-05 | 0.005872452 | yes | down | 0.27 | 0.13 | 0.23 | 0 | 0 | 0.04 | 0.21 | 0.013333333 |
| ENSMUSG00000030247 | Kcnj8 | potassium inwardly-rectifying channel, subfamily J, member 8 [Source:MGI Symbol;Acc:MGI:1100508] | 0.437 | -1.19430906 | 8.80E-06 | 0.001071964 | yes | down | 1.88 | 2.86 | 2.91 | 1.05 | 1.14 | 1.05 | 2.55 | 1.08 |
| ENSMUSG00000024694 | Keg1 | kidney expressed gene 1 [Source:MGI Symbol;Acc:MGI:1928492] | 0.345 | -1.53706332 | 1.67E-20 | 4.02E-17 | yes | down | 1304.98 | 1113.73 | 1292.33 | 347.09 | 439.93 | 485.98 | 1237.013333 | 424.3333333 |
| ENSMUSG00000026866 | Kynu | kynureninase (L-kynurenine hydrolase) [Source:MGI Symbol;Acc:MGI:1918039] | 2.519 | 1.332589078 | 8.87E-06 | 0.001072175 | yes | up | 4.98 | 9.16 | 4.05 | 8.77 | 21.71 | 14.47 | 6.063333333 | 14.98333333 |
| ENSMUSG00000041782 | Lad1 | ladinin [Source:MGI Symbol;Acc:MGI:109343] | 1.45 | 0.535557178 | 0.000196558 | 0.012891989 | yes | up | 14.92 | 19.29 | 14.34 | 18.74 | 27.13 | 24.13 | 16.18333333 | 23.33333333 |
| ENSMUSG00000026822 | Lcn2 | lipocalin 2 [Source:MGI Symbol;Acc:MGI:96757] | 13.885 | 3.795426134 | 0.000401405 | 0.022940134 | yes | up | 2.58 | 3.53 | 1.87 | 15.38 | 96.9 | 4.89 | 2.66 | 39.05666667 |
| ENSMUSG00000031958 | Ldhd | lactate dehydrogenase D [Source:MGI Symbol;Acc:MGI:106428] | 0.384 | -1.381354129 | 7.18E-15 | 7.10E-12 | yes | down | 243.48 | 259.48 | 318.29 | 106.37 | 119.1 | 90.45 | 273.75 | 105.3066667 |
| ENSMUSG00000068220 | Lgals1 | lectin, galactose binding, soluble 1 [Source:MGI Symbol;Acc:MGI:96777] | 1.849 | 0.88657503 | 0.000979474 | 0.044485435 | yes | up | 50.5 | 57.89 | 44.78 | 91.48 | 135.64 | 54.73 | 51.05666667 | 93.95 |
| ENSMUSG00000050335 | Lgals3 | lectin, galactose binding, soluble 3 [Source:MGI Symbol;Acc:MGI:96778] | 1.584 | 0.664017361 | 0.001009081 | 0.045212227 | yes | up | 22.39 | 24.9 | 19.31 | 29.44 | 46.67 | 27.41 | 22.2 | 34.50666667 |
| ENSMUSG00000024766 | Lipo3 | lipase, member O3 [Source:MGI Symbol;Acc:MGI:2147592] | 0.522 | -0.936620073 | 0.000745273 | 0.035879899 | yes | down | 10.73 | 8.73 | 18.02 | 6.27 | 6.28 | 6.72 | 12.49333333 | 6.423333333 |
| ENSMUSG00000033446 | Lpar6 | lysophosphatidic acid receptor 6 [Source:MGI Symbol;Acc:MGI:1914418] | 0.529 | -0.919492896 | 1.88E-05 | 0.001993192 | yes | down | 2.14 | 2.46 | 2.25 | 0.92 | 1.68 | 1.03 | 2.283333333 | 1.21 |
| ENSMUSG00000015568 | Lpl | lipoprotein lipase [Source:MGI Symbol;Acc:MGI:96820] | 0.405 | -1.303889689 | 2.96E-09 | 9.94E-07 | yes | down | 42.89 | 49.3 | 74.7 | 18.61 | 22.61 | 25.66 | 55.63 | 22.29333333 |
| ENSMUSG00000020377 | Ltc4s | leukotriene C4 synthase [Source:MGI Symbol;Acc:MGI:107498] | 3.803 | 1.92726161 | 9.63E-05 | 0.007472112 | yes | up | 0.93 | 1.72 | 1 | 4.93 | 5.29 | 2.98 | 1.216666667 | 4.4 |
| ENSMUSG00000032496 | Ltf | lactotransferrin [Source:MGI Symbol;Acc:MGI:96837] | 17.552 | 4.133562191 | 1.74E-05 | 0.001881652 | yes | up | 0.01 | 0.35 | 0.03 | 3.44 | 1.49 | 0.47 | 0.13 | 1.8 |
| ENSMUSG00000044313 | Mab21l3 | mab-21-like 3 (C. elegans) [Source:MGI Symbol;Acc:MGI:2446273] | 4.513 | 2.173940586 | 2.87E-09 | 9.85E-07 | yes | up | 0.3 | 0.33 | 0.3 | 1.61 | 1.61 | 0.93 | 0.31 | 1.383333333 |
| ENSMUSG00000040147 | Maob | monoamine oxidase B [Source:MGI Symbol;Acc:MGI:96916] | 4.366 | 2.126476323 | 3.57E-08 | 9.10E-06 | yes | up | 0.97 | 0.55 | 1.04 | 1.88 | 5.64 | 4.16 | 0.853333333 | 3.893333333 |
| ENSMUSG00000004864 | Mapk13 | mitogen-activated protein kinase 13 [Source:MGI Symbol;Acc:MGI:1346864] | 2.204 | 1.140051089 | 0.000554283 | 0.02937874 | yes | up | 4.21 | 5.34 | 2.98 | 7.22 | 14.75 | 5.72 | 4.176666667 | 9.23 |
| ENSMUSG00000028979 | Masp2 | mannan-binding lectin serine peptidase 2 [Source:MGI Symbol;Acc:MGI:1330832] | 0.076 | -3.723764907 | 0.000160393 | 0.011069592 | yes | down | 1.9 | 0.4 | 3.12 | 0.24 | 0.14 | 0.08 | 1.806666667 | 0.153333333 |
| ENSMUSG00000026355 | Mcm6 | minichromosome maintenance complex component 6 [Source:MGI Symbol;Acc:MGI:1298227] | 2.024 | 1.017265444 | 0.000729401 | 0.035216661 | yes | up | 2.6 | 3.56 | 3.49 | 3.14 | 10.22 | 6.74 | 3.216666667 | 6.7 |
| ENSMUSG00000002274 | Metrn | meteorin, glial cell differentiation regulator [Source:MGI Symbol;Acc:MGI:1917333] | 1.644 | 0.717305922 | 0.001170153 | 0.049686057 | yes | up | 10.82 | 13.84 | 10.56 | 20.76 | 17.48 | 17.58 | 11.74 | 18.60666667 |
| ENSMUSG00000028655 | Mfsd2a | major facilitator superfamily domain containing 2A [Source:MGI Symbol;Acc:MGI:1923824] | 0.211 | -2.246261783 | 6.02E-06 | 0.000807882 | yes | down | 10 | 2.19 | 3.46 | 0.95 | 1.14 | 1.19 | 5.216666667 | 1.093333333 |
| ENSMUSG00000066595 | Mfsd7b | major facilitator superfamily domain containing 7B [Source:MGI Symbol;Acc:MGI:2444881] | 1.852 | 0.889136643 | 0.001153954 | 0.049587574 | yes | up | 2.95 | 3.24 | 2.11 | 5.92 | 5.41 | 3.45 | 2.766666667 | 4.926666667 |
| ENSMUSG00000026110 | Mgat4a | mannoside acetylglucosaminyltransferase 4, isoenzyme A [Source:MGI Symbol;Acc:MGI:2662992] | 1.52 | 0.60365911 | 0.000963541 | 0.044066571 | yes | up | 2.2 | 2.52 | 1.75 | 3.35 | 3.5 | 2.97 | 2.156666667 | 3.273333333 |
| ENSMUSG00000054612 | Mgmt | O-6-methylguanine-DNA methyltransferase [Source:MGI Symbol;Acc:MGI:96977] | 2.427 | 1.279373453 | 0.000497565 | 0.027143113 | yes | up | 0.45 | 0.4 | 0.32 | 0.77 | 1.23 | 0.85 | 0.39 | 0.95 |
| ENSMUSG00000008540 | Mgst1 | microsomal glutathione S-transferase 1 [Source:MGI Symbol;Acc:MGI:1913850] | 2.038 | 1.027256503 | 8.73E-05 | 0.006853899 | yes | up | 137.22 | 102.6 | 111.96 | 143.95 | 412.77 | 238.22 | 117.26 | 264.98 |
| ENSMUSG00000026688 | Mgst3 | microsomal glutathione S-transferase 3 [Source:MGI Symbol;Acc:MGI:1913697] | 1.789 | 0.839521028 | 1.15E-06 | 0.000184383 | yes | up | 97.59 | 103.08 | 74.68 | 147.6 | 196.69 | 145.19 | 91.78333333 | 163.16 |
| ENSMUSG00000033307 | Mif | macrophage migration inhibitory factor (glycosylation-inhibiting factor) [Source:MGI Symbol;Acc:MGI:96982] | 1.665 | 0.735101468 | 2.72E-05 | 0.002654949 | yes | up | 336.84 | 424.25 | 325.11 | 449.61 | 819.06 | 545.68 | 362.0666667 | 604.7833333 |
| ENSMUSG00000050578 | Mmp13 | matrix metallopeptidase 13 [Source:MGI Symbol;Acc:MGI:1340026] | 0.216 | -2.210354211 | 0.000664086 | 0.033274232 | yes | down | 0.37 | 0.38 | 0.65 | 0.15 | 0.1 | 0.05 | 0.466666667 | 0.1 |
| ENSMUSG00000018623 | Mmp7 | matrix metallopeptidase 7 [Source:MGI Symbol;Acc:MGI:103189] | 90.144 | 6.494157699 | 0.00071072 | 0.034619319 | yes | up | 0.09 | 0 | 0 | 5.49 | 2.53 | 0.2 | 0.03 | 2.74 |
| ENSMUSG00000090272 | Mndal | myeloid nuclear differentiation antigen like [Source:MGI Symbol;Acc:MGI:3780953] | 0.479 | -1.061018889 | 0.000132839 | 0.009574329 | yes | down | 4.02 | 5.06 | 3.82 | 1.01 | 2.72 | 2.73 | 4.3 | 2.153333333 |
| ENSMUSG00000016386 | Mpped2 | metallophosphoesterase domain containing 2 [Source:MGI Symbol;Acc:MGI:1924265] | 0.393 | -1.34609271 | 2.51E-05 | 0.00249276 | yes | down | 2.61 | 1.39 | 1.45 | 0.82 | 1.3 | 0.73 | 1.816666667 | 0.95 |
| ENSMUSG00000022679 | Mpv17l | Mpv17 transgene, kidney disease mutant-like [Source:MGI Symbol;Acc:MGI:2135951] | 0.437 | -1.193331298 | 2.30E-05 | 0.002311768 | yes | down | 146.56 | 87.98 | 150.22 | 62.61 | 41.64 | 62.5 | 128.2533333 | 55.58333333 |
| ENSMUSG00000032092 | Mpzl2 | myelin protein zero-like 2 [Source:MGI Symbol;Acc:MGI:1289160] | 1.909 | 0.933077577 | 1.29E-05 | 0.001489113 | yes | up | 2.77 | 4.03 | 2.35 | 5.28 | 6.78 | 5.16 | 3.05 | 5.74 |
| ENSMUSG00000075279 | Mrpl23-ps1 | mitichondrial ribosomal protein L23, pseudogene 1 [Source:MGI Symbol;Acc:MGI:3650679] | 1.494 | 0.578789996 | 0.000564046 | 0.029615964 | yes | up | 63.16 | 65.61 | 51.48 | 73.64 | 106.75 | 88.88 | 60.08333333 | 89.75666667 |
| ENSMUSG00000040269 | Mrps28 | mitochondrial ribosomal protein S28 [Source:MGI Symbol;Acc:MGI:1913480] | 1.525 | 0.609270483 | 0.00010736 | 0.008089084 | yes | up | 29.71 | 36.98 | 29.82 | 38.83 | 60.22 | 47.59 | 32.17 | 48.88 |
| ENSMUSG00000056290 | Ms4a4b | membrane-spanning 4-domains, subfamily A, member 4B [Source:MGI Symbol;Acc:MGI:1913083] | 0.213 | -2.23136142 | 0.000468686 | 0.025905328 | yes | down | 1.02 | 1.09 | 1.22 | 0.24 | 0.12 | 0.29 | 1.11 | 0.216666667 |
| ENSMUSG00000031765 | Mt1 | metallothionein 1 [Source:MGI Symbol;Acc:MGI:97171] | 1.763 | 0.817626952 | 0.001030622 | 0.045690664 | yes | up | 146.15 | 108.61 | 93.91 | 196.89 | 266.22 | 156.76 | 116.2233333 | 206.6233333 |
| ENSMUSG00000028991 | Mtor | mechanistic target of rapamycin (serine/threonine kinase) [Source:MGI Symbol;Acc:MGI:1928394] | 0.675 | -0.567432673 | 0.000196615 | 0.012891989 | yes | down | 18.22 | 15.65 | 16.86 | 11.66 | 12.16 | 11.59 | 16.91 | 11.80333333 |
| ENSMUSG00000022353 | Mtss1 | metastasis suppressor 1 [Source:MGI Symbol;Acc:MGI:2384818] | 0.596 | -0.747737529 | 1.72E-06 | 0.000258321 | yes | down | 10.97 | 11.76 | 9.91 | 4.83 | 7.66 | 6.95 | 10.88 | 6.48 |
| ENSMUSG00000035638 | Muc20 | mucin 20 [Source:MGI Symbol;Acc:MGI:2385039] | 0.311 | -1.684789118 | 0.000935425 | 0.04294264 | yes | down | 5.69 | 5.82 | 0.87 | 1.13 | 1.32 | 1.27 | 4.126666667 | 1.24 |
| ENSMUSG00000034593 | Myo5a | myosin VA [Source:MGI Symbol;Acc:MGI:105976] | 0.448 | -1.157757943 | 5.90E-06 | 0.000806233 | yes | down | 5.33 | 4.53 | 6.28 | 1.96 | 2.14 | 3.95 | 5.38 | 2.683333333 |
| ENSMUSG00000002204 | Napsa | napsin A aspartic peptidase [Source:MGI Symbol;Acc:MGI:109365] | 0.65 | -0.621098514 | 1.06E-05 | 0.001235848 | yes | down | 750.51 | 757.9 | 677.57 | 438.03 | 481.12 | 482.8 | 728.66 | 467.3166667 |
| ENSMUSG00000089694 | Nat8f7 | N-acetyltransferase 8 (GCN5-related) family member 7 [Source:MGI Symbol;Acc:MGI:3782661] | 0.571 | -0.808611082 | 0.000356091 | 0.020993097 | yes | down | 11.58 | 13.46 | 8.9 | 4.38 | 6.76 | 7.85 | 11.31333333 | 6.33 |
| ENSMUSG00000004558 | Ndrg2 | N-myc downstream regulated gene 2 [Source:MGI Symbol;Acc:MGI:1352498] | 0.669 | -0.579520426 | 0.000198206 | 0.012907976 | yes | down | 26.2 | 30.36 | 24.51 | 16.38 | 17.45 | 19.44 | 27.02333333 | 17.75666667 |
| ENSMUSG00000002379 | Ndufa11 | NADH dehydrogenase (ubiquinone) 1 alpha subcomplex 11 [Source:MGI Symbol;Acc:MGI:1917125] | 1.36 | 0.443971256 | 0.000786367 | 0.0372184 | yes | up | 60.91 | 70.04 | 54.07 | 77.31 | 100.38 | 80.24 | 61.67333333 | 85.97666667 |
| ENSMUSG00000071014 | Ndufb6 | NADH dehydrogenase (ubiquinone) 1 beta subcomplex, 6 [Source:MGI Symbol;Acc:MGI:2684983] | 1.533 | 0.616458796 | 3.99E-07 | 7.53E-05 | yes | up | 169.69 | 207.64 | 177.31 | 254.28 | 319.98 | 266.59 | 184.88 | 280.2833333 |
| ENSMUSG00000033938 | Ndufb7 | NADH dehydrogenase (ubiquinone) 1 beta subcomplex, 7 [Source:MGI Symbol;Acc:MGI:1914166] | 1.392 | 0.477358511 | 0.000287341 | 0.017620074 | yes | up | 207.43 | 222.51 | 190.71 | 254.57 | 304.91 | 299.95 | 206.8833333 | 286.4766667 |
| ENSMUSG00000025204 | Ndufb8 | NADH dehydrogenase (ubiquinone) 1 beta subcomplex 8 [Source:MGI Symbol;Acc:MGI:1914514] | 1.46 | 0.545593812 | 3.47E-05 | 0.003219198 | yes | up | 400.25 | 429.68 | 368.84 | 538.8 | 674.29 | 543.24 | 399.59 | 585.4433333 |
| ENSMUSG00000026749 | Nek6 | NIMA (never in mitosis gene a)-related expressed kinase 6 [Source:MGI Symbol;Acc:MGI:1891638] | 1.532 | 0.615763308 | 0.000726221 | 0.035164142 | yes | up | 3.94 | 2.81 | 4.02 | 4.82 | 5.82 | 4.38 | 3.59 | 5.006666667 |
| ENSMUSG00000027967 | Neurog2 | neurogenin 2 [Source:MGI Symbol;Acc:MGI:109619] | 0.01 | -6.64994072 | 1.39E-06 | 0.000216986 | yes | down | 0.3 | 0.36 | 0.47 | 0 | 0 | 0 | 0.376666667 | 0 |
| ENSMUSG00000041309 | Nkx6-2 | NK6 homeobox 2 [Source:MGI Symbol;Acc:MGI:1352738] | 15.742 | 3.976554594 | 0.000438217 | 0.024864253 | yes | up | 0.04 | 0 | 0.11 | 0.98 | 0.12 | 0.69 | 0.05 | 0.596666667 |
| ENSMUSG00000042988 | Notum | notum pectinacetylesterase homolog (Drosophila) [Source:MGI Symbol;Acc:MGI:1924833] | 0.613 | -0.706326212 | 0.000775413 | 0.036907919 | yes | down | 4.83 | 6.11 | 5.67 | 2.26 | 4.14 | 3.53 | 5.536666667 | 3.31 |
| ENSMUSG00000042684 | Npl | N-acetylneuraminate pyruvate lyase [Source:MGI Symbol;Acc:MGI:1921341] | 3.047 | 1.60720166 | 4.35E-18 | 8.12E-15 | yes | up | 22.84 | 27.19 | 21.37 | 60.84 | 98.35 | 57.99 | 23.8 | 72.39333333 |
| ENSMUSG00000022206 | Npr3 | natriuretic peptide receptor 3 [Source:MGI Symbol;Acc:MGI:97373] | 0.547 | -0.871270963 | 0.000389951 | 0.02243818 | yes | down | 1.21 | 1.48 | 1.71 | 0.53 | 0.85 | 1 | 1.466666667 | 0.793333333 |
| ENSMUSG00000025582 | Nptx1 | neuronal pentraxin 1 [Source:MGI Symbol;Acc:MGI:107811] | 9.538 | 3.25375177 | 2.06E-07 | 4.23E-05 | yes | up | 0.07 | 0.03 | 0.05 | 0.74 | 0.36 | 0.27 | 0.05 | 0.456666667 |
| ENSMUSG00000022809 | Nr1i2 | nuclear receptor subfamily 1, group I, member 2 [Source:MGI Symbol;Acc:MGI:1337040] | 2.246 | 1.167220394 | 2.91E-05 | 0.002750691 | yes | up | 0.86 | 0.92 | 0.89 | 2.01 | 1.92 | 1.94 | 0.89 | 1.956666667 |
| ENSMUSG00000022186 | Oxct1 | 3-oxoacid CoA transferase 1 [Source:MGI Symbol;Acc:MGI:1914291] | 0.553 | -0.855581486 | 0.000648933 | 0.032889984 | yes | down | 333.98 | 289.24 | 385.01 | 221.24 | 145.01 | 173.06 | 336.0766667 | 179.77 |
| ENSMUSG00000028370 | Pappa | pregnancy-associated plasma protein A [Source:MGI Symbol;Acc:MGI:97479] | 2.843 | 1.50762673 | 2.03E-09 | 7.25E-07 | yes | up | 0.19 | 0.19 | 0.2 | 0.49 | 0.6 | 0.55 | 0.193333333 | 0.546666667 |
| ENSMUSG00000024899 | Papss2 | 3'-phosphoadenosine 5'-phosphosulfate synthase 2 [Source:MGI Symbol;Acc:MGI:1330223] | 0.621 | -0.688408338 | 0.000318544 | 0.019321919 | yes | down | 54.85 | 76.18 | 72.45 | 40.03 | 35.21 | 47.33 | 67.82666667 | 40.85666667 |
| ENSMUSG00000021725 | Parp8 | poly (ADP-ribose) polymerase family, member 8 [Source:MGI Symbol;Acc:MGI:1098713] | 0.536 | -0.900739197 | 3.87E-05 | 0.003511566 | yes | down | 2.66 | 3.12 | 2.63 | 1.34 | 1.43 | 1.66 | 2.803333333 | 1.476666667 |
| ENSMUSG00000030671 | Pde3b | phosphodiesterase 3B, cGMP-inhibited [Source:MGI Symbol;Acc:MGI:1333863] | 1.638 | 0.712063979 | 0.00043983 | 0.024864253 | yes | up | 1.33 | 1.15 | 1.29 | 1.8 | 2.32 | 2.13 | 1.256666667 | 2.083333333 |
| ENSMUSG00000006494 | Pdk1 | pyruvate dehydrogenase kinase, isoenzyme 1 [Source:MGI Symbol;Acc:MGI:1926119] | 2.207 | 1.142044275 | 4.77E-11 | 2.43E-08 | yes | up | 4.61 | 5.2 | 3.75 | 8.93 | 14.01 | 9.58 | 4.52 | 10.84 |
| ENSMUSG00000035232 | Pdk3 | pyruvate dehydrogenase kinase, isoenzyme 3 [Source:MGI Symbol;Acc:MGI:2384308] | 0.637 | -0.651285452 | 0.000502007 | 0.027296844 | yes | down | 17.66 | 14.15 | 16.66 | 9.29 | 12.29 | 10 | 16.15666667 | 10.52666667 |
| ENSMUSG00000041237 | Pklr | pyruvate kinase liver and red blood cell [Source:MGI Symbol;Acc:MGI:97604] | 2.406 | 1.266352334 | 1.65E-05 | 0.001811861 | yes | up | 4.79 | 8.35 | 8.44 | 9.03 | 28.59 | 12.39 | 7.193333333 | 16.67 |
| ENSMUSG00000021822 | Plau | plasminogen activator, urokinase [Source:MGI Symbol;Acc:MGI:97611] | 0.65 | -0.62227471 | 0.000652444 | 0.03292002 | yes | down | 64.91 | 111.05 | 79.28 | 40.46 | 68.55 | 55.2 | 85.08 | 54.73666667 |
| ENSMUSG00000064247 | Plcxd1 | phosphatidylinositol-specific phospholipase C, X domain containing 1 [Source:MGI Symbol;Acc:MGI:2685422] | 2.211 | 1.144473698 | 3.66E-05 | 0.003341456 | yes | up | 0.81 | 0.98 | 1.25 | 2.39 | 2.28 | 2.35 | 1.013333333 | 2.34 |
| ENSMUSG00000024521 | Pmaip1 | phorbol-12-myristate-13-acetate-induced protein 1 [Source:MGI Symbol;Acc:MGI:1930146] | 1.933 | 0.950968729 | 0.000203046 | 0.013121445 | yes | up | 2.83 | 3.21 | 4.24 | 7.29 | 5.56 | 6.42 | 3.426666667 | 6.423333333 |
| ENSMUSG00000022325 | Pop1 | processing of precursor 1, ribonuclease P/MRP family, (S. cerevisiae) [Source:MGI Symbol;Acc:MGI:1914974] | 0.538 | -0.893322186 | 0.000151405 | 0.010599592 | yes | down | 5.08 | 3.53 | 4.73 | 1.51 | 2.37 | 2.91 | 4.446666667 | 2.263333333 |
| ENSMUSG00000038151 | Prdm1 | PR domain containing 1, with ZNF domain [Source:MGI Symbol;Acc:MGI:99655] | 0.252 | -1.989661211 | 0.000998317 | 0.044849543 | yes | down | 0.13 | 0.37 | 0.36 | 0.06 | 0.12 | 0.04 | 0.286666667 | 0.073333333 |
| ENSMUSG00000032601 | Prkar2a | protein kinase, cAMP dependent regulatory, type II alpha [Source:MGI Symbol;Acc:MGI:108025] | 1.389 | 0.474404904 | 0.000576138 | 0.030062969 | yes | up | 6.58 | 7.46 | 7.25 | 8.2 | 10.37 | 13.78 | 7.096666667 | 10.78333333 |
| ENSMUSG00000070368 | Prok1 | prokineticin 1 [Source:MGI Symbol;Acc:MGI:2180370] | 0.291 | -1.779819168 | 6.11E-05 | 0.00502957 | yes | down | 2.55 | 2.81 | 2.63 | 0.18 | 1 | 1.21 | 2.663333333 | 0.796666667 |
| ENSMUSG00000027376 | Prom2 | prominin 2 [Source:MGI Symbol;Acc:MGI:2138997] | 0.493 | -1.019601662 | 0.000197193 | 0.012891989 | yes | down | 1.77 | 2.13 | 1.16 | 0.76 | 0.83 | 0.85 | 1.686666667 | 0.813333333 |
| ENSMUSG00000019464 | Ptger1 | prostaglandin E receptor 1 (subtype EP1) [Source:MGI Symbol;Acc:MGI:97793] | 0.474 | -1.075861793 | 0.000117973 | 0.008693754 | yes | down | 2.01 | 1.87 | 1.22 | 0.93 | 0.77 | 0.86 | 1.7 | 0.853333333 |
| ENSMUSG00000032487 | Ptgs2 | prostaglandin-endoperoxide synthase 2 [Source:MGI Symbol;Acc:MGI:97798] | 7.785 | 2.960744703 | 5.60E-05 | 0.004775412 | yes | up | 0.03 | 0.04 | 0.03 | 0.28 | 0.31 | 0.14 | 0.033333333 | 0.243333333 |
| ENSMUSG00000056553 | Ptprn2 | protein tyrosine phosphatase, receptor type, N polypeptide 2 [Source:MGI Symbol;Acc:MGI:107418] | 6.06 | 2.599208284 | 0.000819668 | 0.038279178 | yes | up | 0.05 | 0.14 | 0.04 | 0.33 | 0.17 | 0.54 | 0.076666667 | 0.346666667 |
| ENSMUSG00000019478 | Rab4a | RAB4A, member RAS oncogene family [Source:MGI Symbol;Acc:MGI:105069] | 1.488 | 0.573133052 | 4.52E-05 | 0.004014777 | yes | up | 18.02 | 22.3 | 22.29 | 26.6 | 35.85 | 30.65 | 20.87 | 31.03333333 |
| ENSMUSG00000020684 | Rasl10b | RAS-like, family 10, member B [Source:MGI Symbol;Acc:MGI:2685575] | 3.43 | 1.778179477 | 0.000218012 | 0.013981043 | yes | up | 0.25 | 0.25 | 0.1 | 0.56 | 0.81 | 0.55 | 0.2 | 0.64 |
| ENSMUSG00000029641 | Rasl11a | RAS-like, family 11, member A [Source:MGI Symbol;Acc:MGI:1916145] | 1.998 | 0.998662107 | 0.000703693 | 0.034470696 | yes | up | 3.27 | 3.93 | 3.11 | 7.87 | 6.59 | 5.26 | 3.436666667 | 6.573333333 |
| ENSMUSG00000041696 | Rasl12 | RAS-like, family 12 [Source:MGI Symbol;Acc:MGI:1918034] | 3 | 1.584896339 | 0.000113879 | 0.008429041 | yes | up | 1.89 | 1.36 | 1.14 | 4.37 | 7.05 | 1.94 | 1.463333333 | 4.453333333 |
| ENSMUSG00000025350 | Rdh5 | retinol dehydrogenase 5 [Source:MGI Symbol;Acc:MGI:1201412] | 0.559 | -0.83859254 | 0.000536232 | 0.028666065 | yes | down | 12.36 | 11.59 | 11.28 | 6.2 | 6.53 | 6.61 | 11.74333333 | 6.446666667 |
| ENSMUSG00000034226 | Rhov | ras homolog family member V [Source:MGI Symbol;Acc:MGI:2444227] | 0.46 | -1.120488901 | 0.001047054 | 0.046296305 | yes | down | 1.82 | 4.66 | 3.01 | 1.16 | 1.61 | 1.47 | 3.163333333 | 1.413333333 |
| ENSMUSG00000001313 | Rnd2 | Rho family GTPase 2 [Source:MGI Symbol;Acc:MGI:1338755] | 2.24 | 1.163217296 | 1.70E-10 | 7.94E-08 | yes | up | 6.37 | 8.4 | 7.8 | 14.49 | 20.97 | 14.72 | 7.523333333 | 16.72666667 |
| ENSMUSG00000048911 | Rnf24 | ring finger protein 24 [Source:MGI Symbol;Acc:MGI:1261771] | 0.462 | -1.115480865 | 2.58E-05 | 0.002536398 | yes | down | 4.74 | 2.87 | 5.07 | 1.74 | 1.92 | 2.08 | 4.226666667 | 1.913333333 |
| ENSMUSG00000056071 | S100a9 | S100 calcium binding protein A9 (calgranulin B) [Source:MGI Symbol;Acc:MGI:1338947] | 5.694 | 2.509538919 | 1.53E-06 | 0.000233665 | yes | up | 2.96 | 4.77 | 2.59 | 24.07 | 27 | 5.84 | 3.44 | 18.97 |
| ENSMUSG00000031609 | Sap30 | sin3 associated polypeptide [Source:MGI Symbol;Acc:MGI:1929129] | 0.507 | -0.97993967 | 0.000346384 | 0.020492762 | yes | down | 27.33 | 37.14 | 48.05 | 15.88 | 14.29 | 25.61 | 37.50666667 | 18.59333333 |
| ENSMUSG00000023236 | Scg5 | secretogranin V [Source:MGI Symbol;Acc:MGI:98289] | 2.662 | 1.412252954 | 2.37E-10 | 1.07E-07 | yes | up | 6.18 | 6.29 | 4.1 | 13.32 | 15.96 | 14.42 | 5.523333333 | 14.56666667 |
| ENSMUSG00000054986 | Sec14l3 | SEC14-like lipid binding 3 [Source:MGI Symbol;Acc:MGI:3617848] | 0.023 | -5.414695533 | 7.06E-11 | 3.49E-08 | yes | down | 1.65 | 1.21 | 0.58 | 0 | 0.08 | 0.05 | 1.146666667 | 0.043333333 |
| ENSMUSG00000026249 | Serpine2 | serine (or cysteine) peptidase inhibitor, clade E, member 2 [Source:MGI Symbol;Acc:MGI:101780] | 1.752 | 0.809383005 | 0.001171032 | 0.049686057 | yes | up | 7.36 | 6.79 | 4.85 | 10.36 | 14.71 | 8.2 | 6.333333333 | 11.09 |
| ENSMUSG00000038224 | Serpinf2 | serine (or cysteine) peptidase inhibitor, clade F, member 2 [Source:MGI Symbol;Acc:MGI:107173] | 0.575 | -0.799095748 | 1.07E-08 | 2.96E-06 | yes | down | 49.67 | 68.72 | 66.75 | 32.21 | 39.17 | 33.83 | 61.71333333 | 35.07 |
| ENSMUSG00000032009 | Sesn3 | sestrin 3 [Source:MGI Symbol;Acc:MGI:1922997] | 1.466 | 0.552176982 | 0.001101918 | 0.047717599 | yes | up | 2.16 | 2.8 | 2.87 | 2.79 | 4.18 | 4.33 | 2.61 | 3.766666667 |
| ENSMUSG00000021795 | Sftpd | surfactant associated protein D [Source:MGI Symbol;Acc:MGI:109515] | 43.053 | 5.428038194 | 0.000691968 | 0.034226001 | yes | up | 0 | 0.04 | 0 | 1.13 | 0.36 | 0.08 | 0.013333333 | 0.523333333 |
| ENSMUSG00000024042 | Sik1 | salt inducible kinase 1 [Source:MGI Symbol;Acc:MGI:104754] | 0.659 | -0.602312968 | 0.000660768 | 0.033240185 | yes | down | 5.06 | 5.29 | 4.24 | 2.68 | 3.32 | 3.53 | 4.863333333 | 3.176666667 |
| ENSMUSG00000001995 | Sipa1l2 | signal-induced proliferation-associated 1 like 2 [Source:MGI Symbol;Acc:MGI:2676970] | 1.749 | 0.806193516 | 0.000102053 | 0.00781705 | yes | up | 0.8 | 1.04 | 0.89 | 1.27 | 2.05 | 1.41 | 0.91 | 1.576666667 |
| ENSMUSG00000032902 | Slc16a1 | solute carrier family 16 (monocarboxylic acid transporters), member 1 [Source:MGI Symbol;Acc:MGI:106013] | 1.64 | 0.713981823 | 0.000111168 | 0.008335372 | yes | up | 3.86 | 4.9 | 4.69 | 7.33 | 8.38 | 5.93 | 4.483333333 | 7.213333333 |
| ENSMUSG00000026220 | Slc16a14 | solute carrier family 16 (monocarboxylic acid transporters), member 14 [Source:MGI Symbol;Acc:MGI:1919031] | 0.611 | -0.710159 | 0.000283552 | 0.017515576 | yes | down | 17.51 | 13.5 | 13.23 | 8.68 | 10.21 | 7.59 | 14.74666667 | 8.826666667 |
| ENSMUSG00000041920 | Slc16a6 | solute carrier family 16 (monocarboxylic acid transporters), member 6 [Source:MGI Symbol;Acc:MGI:2144585] | 1.634 | 0.70831149 | 0.000682565 | 0.033930357 | yes | up | 2.38 | 2.09 | 2.38 | 3.08 | 4.13 | 3.75 | 2.283333333 | 3.653333333 |
| ENSMUSG00000001436 | Slc19a1 | solute carrier family 19 (folate transporter), member 1 [Source:MGI Symbol;Acc:MGI:103182] | 1.438 | 0.524211644 | 0.000321143 | 0.019409529 | yes | up | 27.28 | 32.15 | 28.62 | 41.73 | 44.02 | 38.15 | 29.35 | 41.3 |
| ENSMUSG00000000154 | Slc22a18 | solute carrier family 22 (organic cation transporter), member 18 [Source:MGI Symbol;Acc:MGI:1336884] | 0.662 | -0.596127671 | 4.65E-05 | 0.004098077 | yes | down | 122.89 | 142.47 | 135.78 | 82.72 | 85.16 | 99.16 | 133.7133333 | 89.01333333 |
| ENSMUSG00000024757 | Slc22a19 | solute carrier family 22 (organic anion transporter), member 19 [Source:MGI Symbol;Acc:MGI:2442751] | 0.173 | -2.529348312 | 1.41E-15 | 1.69E-12 | yes | down | 20.57 | 15.92 | 25.31 | 4.79 | 3.15 | 2.43 | 20.6 | 3.456666667 |
| ENSMUSG00000053303 | Slc22a26 | solute carrier family 22 (organic cation transporter), member 26 [Source:MGI Symbol;Acc:MGI:2385316] | 0.068 | -3.87735448 | 1.65E-44 | 2.77E-40 | yes | down | 6.12 | 6.03 | 8.23 | 0.34 | 0.61 | 0.5 | 6.793333333 | 0.483333333 |
| ENSMUSG00000075044 | Slc22a29 | solute carrier family 22. member 29 [Source:MGI Symbol;Acc:MGI:3605624] | 2.723 | 1.445457309 | 9.65E-06 | 0.001141265 | yes | up | 1.7 | 3.44 | 1.68 | 2.42 | 6.17 | 5.93 | 2.273333333 | 4.84 |
| ENSMUSG00000052562 | Slc22a30 | solute carrier family 22, member 30 [Source:MGI Symbol;Acc:MGI:2442750] | 0.503 | -0.990384555 | 1.36E-09 | 5.21E-07 | yes | down | 54.49 | 54.85 | 65.54 | 26.7 | 29.25 | 31.41 | 58.29333333 | 29.12 |
| ENSMUSG00000067144 | Slc22a7 | solute carrier family 22 (organic anion transporter), member 7 [Source:MGI Symbol;Acc:MGI:1859559] | 0.032 | -4.969796877 | 1.11E-09 | 4.34E-07 | yes | down | 14.58 | 13.91 | 22.93 | 0.94 | 0.13 | 0.44 | 17.14 | 0.503333333 |
| ENSMUSG00000027359 | Slc27a2 | solute carrier family 27 (fatty acid transporter), member 2 [Source:MGI Symbol;Acc:MGI:1347099] | 0.679 | -0.558152992 | 1.43E-06 | 0.00022046 | yes | down | 921.36 | 1004.17 | 1015.53 | 594.38 | 723.67 | 650.2 | 980.3533333 | 656.0833333 |
| ENSMUSG00000053897 | Slc39a8 | solute carrier family 39 (metal ion transporter), member 8 [Source:MGI Symbol;Acc:MGI:1914797] | 0.585 | -0.773638608 | 0.00082017 | 0.038279178 | yes | down | 16.38 | 12.34 | 17.48 | 7.83 | 7.98 | 11.03 | 15.4 | 8.946666667 |
| ENSMUSG00000029650 | Slc46a3 | solute carrier family 46, member 3 [Source:MGI Symbol;Acc:MGI:1918956] | 0.431 | -1.213792427 | 9.88E-05 | 0.007618585 | yes | down | 11.25 | 11.08 | 14.43 | 2.28 | 5.49 | 5.04 | 12.25333333 | 4.27 |
| ENSMUSG00000053862 | Slc51b | solute carrier family 51, beta subunit [Source:MGI Symbol;Acc:MGI:3582052] | 1.726 | 0.787458103 | 1.41E-07 | 3.08E-05 | yes | up | 44.68 | 46.41 | 50.66 | 70.9 | 87.79 | 84.56 | 47.25 | 81.08333333 |
| ENSMUSG00000042371 | Slc5a10 | solute carrier family 5 (sodium/glucose cotransporter), member 10 [Source:MGI Symbol;Acc:MGI:1926089] | 2.411 | 1.269582118 | 3.73E-07 | 7.11E-05 | yes | up | 27.55 | 40.83 | 42.85 | 69.12 | 138.63 | 62.62 | 37.07666667 | 90.12333333 |
| ENSMUSG00000030109 | Slc6a12 | solute carrier family 6 (neurotransmitter transporter, betaine/GABA), member 12 [Source:MGI Symbol;Acc:MGI:95628] | 5.862 | 2.551312561 | 1.87E-09 | 6.84E-07 | yes | up | 0.67 | 0.99 | 0.46 | 4.54 | 2.4 | 2.43 | 0.706666667 | 3.123333333 |
| ENSMUSG00000041052 | Slc7a13 | solute carrier family 7, (cationic amino acid transporter, y+ system) member 13 [Source:MGI Symbol;Acc:MGI:1921337] | 0.295 | -1.758857561 | 7.15E-09 | 2.18E-06 | yes | down | 211.8 | 244.45 | 239.79 | 83.11 | 36.83 | 75.54 | 232.0133333 | 65.16 |
| ENSMUSG00000054640 | Slc8a1 | solute carrier family 8 (sodium/calcium exchanger), member 1 [Source:MGI Symbol;Acc:MGI:107956] | 2.116 | 1.08114546 | 8.61E-09 | 2.49E-06 | yes | up | 1.94 | 1.74 | 2.13 | 3.05 | 4.69 | 4.57 | 1.936666667 | 4.103333333 |
| ENSMUSG00000039463 | Slc9a8 | solute carrier family 9 (sodium/hydrogen exchanger), member 8 [Source:MGI Symbol;Acc:MGI:1924281] | 0.378 | -1.403379299 | 1.81E-05 | 0.001948004 | yes | down | 6.98 | 9.27 | 19.36 | 2.53 | 5.29 | 4.96 | 11.87 | 4.26 |
| ENSMUSG00000041698 | Slco1a1 | solute carrier organic anion transporter family, member 1a1 [Source:MGI Symbol;Acc:MGI:1351891] | 0.042 | -4.575992194 | 1.90E-05 | 0.001998422 | yes | down | 49.7 | 36.92 | 92.02 | 1.86 | 0.27 | 5.13 | 59.54666667 | 2.42 |
| ENSMUSG00000013611 | Snx31 | sorting nexin 31 [Source:MGI Symbol;Acc:MGI:1913946] | 0.537 | -0.895973868 | 5.01E-05 | 0.004354271 | yes | down | 8.31 | 7.03 | 5.59 | 3.72 | 3.37 | 3.81 | 6.976666667 | 3.633333333 |
| ENSMUSG00000043531 | Sorcs1 | sortilin-related VPS10 domain containing receptor 1 [Source:MGI Symbol;Acc:MGI:1929666] | 0.214 | -2.222883435 | 7.39E-05 | 0.005910312 | yes | down | 1.44 | 0.65 | 0.77 | 0.07 | 0.4 | 0.23 | 0.953333333 | 0.233333333 |
| ENSMUSG00000001494 | Sost | sclerostin [Source:MGI Symbol;Acc:MGI:1921749] | 4.636 | 2.212807568 | 0.000994919 | 0.04484717 | yes | up | 0.04 | 0.21 | 0.38 | 1.17 | 0.54 | 1.02 | 0.21 | 0.91 |
| ENSMUSG00000036169 | Sostdc1 | sclerostin domain containing 1 [Source:MGI Symbol;Acc:MGI:1913292] | 1.61 | 0.68674809 | 2.74E-05 | 0.002662559 | yes | up | 21.52 | 20.81 | 21.54 | 28.03 | 43.98 | 31.15 | 21.29 | 34.38666667 |
| ENSMUSG00000002055 | Spag5 | sperm associated antigen 5 [Source:MGI Symbol;Acc:MGI:1927470] | 2.955 | 1.563264551 | 0.000538144 | 0.028666065 | yes | up | 0.61 | 2.11 | 1.32 | 5.58 | 1.54 | 3.12 | 1.346666667 | 3.413333333 |
| ENSMUSG00000074476 | Spc24 | SPC24, NDC80 kinetochore complex component, homolog (S. cerevisiae) [Source:MGI Symbol;Acc:MGI:1914879] | 2.42 | 1.275088177 | 0.000442893 | 0.024887922 | yes | up | 1.36 | 1.27 | 1.43 | 2.95 | 6.5 | 2.65 | 1.353333333 | 4.033333333 |
| ENSMUSG00000005233 | Spc25 | SPC25, NDC80 kinetochore complex component, homolog (S. cerevisiae) [Source:MGI Symbol;Acc:MGI:1913692] | 2.258 | 1.175156278 | 0.000381593 | 0.022032729 | yes | up | 11.27 | 9.15 | 11.36 | 33.07 | 24.6 | 13.55 | 10.59333333 | 23.74 |
| ENSMUSG00000026295 | Spp2 | secreted phosphoprotein 2 [Source:MGI Symbol;Acc:MGI:1922646] | 1.623 | 0.698703336 | 9.15E-05 | 0.007154367 | yes | up | 189.18 | 189.49 | 174.15 | 315.63 | 281.75 | 281.18 | 184.2733333 | 292.8533333 |
| ENSMUSG00000014813 | Stc1 | stanniocalcin 1 [Source:MGI Symbol;Acc:MGI:109131] | 3.86 | 1.948652172 | 6.13E-18 | 1.03E-14 | yes | up | 0.72 | 1 | 0.86 | 3.18 | 3.14 | 3.39 | 0.86 | 3.236666667 |
| ENSMUSG00000006800 | Sulf2 | sulfatase 2 [Source:MGI Symbol;Acc:MGI:1919293] | 1.634 | 0.708158743 | 0.00022382 | 0.014137678 | yes | up | 3.61 | 4.13 | 2.49 | 4.49 | 8.73 | 5.82 | 3.41 | 6.346666667 |
| ENSMUSG00000027887 | Sypl2 | synaptophysin-like 2 [Source:MGI Symbol;Acc:MGI:1328311] | 0.675 | -0.566158404 | 0.000626991 | 0.032243647 | yes | down | 8.26 | 9.89 | 9.88 | 5.2 | 6.83 | 6.29 | 9.343333333 | 6.106666667 |
| ENSMUSG00000006095 | Tbcb | tubulin folding cofactor B [Source:MGI Symbol;Acc:MGI:1913661] | 1.526 | 0.609950521 | 3.26E-05 | 0.00305574 | yes | up | 36.7 | 45.03 | 34.09 | 54.83 | 61.71 | 58.37 | 38.60666667 | 58.30333333 |
| ENSMUSG00000029925 | Tbxas1 | thromboxane A synthase 1, platelet [Source:MGI Symbol;Acc:MGI:98497] | 0.448 | -1.159168646 | 2.21E-05 | 0.002254314 | yes | down | 3.4 | 6.53 | 5.96 | 2.68 | 2.61 | 2.81 | 5.296666667 | 2.7 |
| ENSMUSG00000021187 | Tc2n | tandem C2 domains, nuclear [Source:MGI Symbol;Acc:MGI:1921663] | 0.342 | -1.547475915 | 1.51E-05 | 0.001677853 | yes | down | 0.75 | 0.59 | 1.07 | 0.38 | 0.34 | 0.26 | 0.803333333 | 0.326666667 |
| ENSMUSG00000050410 | Tcf19 | transcription factor 19 [Source:MGI Symbol;Acc:MGI:103180] | 3.15 | 1.655156101 | 5.93E-05 | 0.004956254 | yes | up | 1.04 | 0.86 | 0.71 | 1.41 | 5.26 | 2.25 | 0.87 | 2.973333333 |
| ENSMUSG00000022797 | Tfrc | transferrin receptor [Source:MGI Symbol;Acc:MGI:98822] | 2.134 | 1.093553307 | 1.04E-06 | 0.000171969 | yes | up | 10.72 | 7.6 | 12.65 | 18.35 | 27.17 | 21.74 | 10.32333333 | 22.42 |
| ENSMUSG00000056665 | Them6 | thioesterase superfamily member 6 [Source:MGI Symbol;Acc:MGI:1925301] | 1.918 | 0.939807814 | 1.28E-06 | 0.000202645 | yes | up | 8.09 | 8.3 | 7.03 | 11.27 | 20.07 | 13.89 | 7.806666667 | 15.07666667 |
| ENSMUSG00000062580 | Timm17a | translocase of inner mitochondrial membrane 17a [Source:MGI Symbol;Acc:MGI:1343131] | 1.445 | 0.530814965 | 0.000374971 | 0.021725019 | yes | up | 47.42 | 53.26 | 64.26 | 64.92 | 79.18 | 51.26 | 54.98 | 65.12 |
| ENSMUSG00000072676 | Tmem254a | transmembrane protein 254a [Source:MGI Symbol;Acc:MGI:1196450] | 0.17 | -2.558272071 | 0.000492602 | 0.026959927 | yes | down | 19.29 | 31.79 | 29.94 | 7.53 | 4.31 | 1.83 | 27.00666667 | 4.556666667 |
| ENSMUSG00000039304 | Tnfsf10 | tumor necrosis factor (ligand) superfamily, member 10 [Source:MGI Symbol;Acc:MGI:107414] | 0.527 | -0.924726447 | 6.05E-06 | 0.000807882 | yes | down | 8.24 | 9.32 | 11.26 | 3.66 | 5.06 | 6.16 | 9.606666667 | 4.96 |
| ENSMUSG00000019787 | Trdn | triadin [Source:MGI Symbol;Acc:MGI:1924007] | 0.357 | -1.485146672 | 2.79E-05 | 0.002664937 | yes | down | 1.55 | 2.93 | 3.43 | 0.64 | 0.91 | 1.5 | 2.636666667 | 1.016666667 |
| ENSMUSG00000042265 | Trem1 | triggering receptor expressed on myeloid cells 1 [Source:MGI Symbol;Acc:MGI:1930005] | 30.084 | 4.910935983 | 0.001115851 | 0.048127096 | yes | up | 0 | 0.02 | 0 | 0.19 | 0.2 | 0.07 | 0.006666667 | 0.153333333 |
| ENSMUSG00000032554 | Trf | transferrin [Source:MGI Symbol;Acc:MGI:98821] | 2.806 | 1.488620716 | 2.86E-05 | 0.002712377 | yes | up | 13.93 | 20.5 | 5.54 | 19.11 | 51.81 | 22.11 | 13.32333333 | 31.01 |
| ENSMUSG00000068735 | Trp53i11 | transformation related protein 53 inducible protein 11 [Source:MGI Symbol;Acc:MGI:2670995] | 0.459 | -1.122538236 | 0.000133341 | 0.009574329 | yes | down | 2.17 | 2.62 | 1.58 | 0.79 | 1.18 | 0.82 | 2.123333333 | 0.93 |
| ENSMUSG00000036899 | Trpv5 | transient receptor potential cation channel, subfamily V, member 5 [Source:MGI Symbol;Acc:MGI:2429764] | 2.207 | 1.141783432 | 0.001060606 | 0.046650027 | yes | up | 1.72 | 1.85 | 2.54 | 2.48 | 8.09 | 3.26 | 2.036666667 | 4.61 |
| ENSMUSG00000028699 | Tspan1 | tetraspanin 1 [Source:MGI Symbol;Acc:MGI:1914055] | 1.76 | 0.815454513 | 4.46E-06 | 0.000624281 | yes | up | 8.38 | 8.96 | 7.54 | 12.09 | 19.15 | 14.32 | 8.293333333 | 15.18666667 |
| ENSMUSG00000027858 | Tspan2 | tetraspanin 2 [Source:MGI Symbol;Acc:MGI:1917997] | 2.409 | 1.268514452 | 8.78E-07 | 0.000152007 | yes | up | 0.93 | 0.82 | 0.79 | 2.06 | 1.78 | 2.16 | 0.846666667 | 2 |
| ENSMUSG00000058254 | Tspan7 | tetraspanin 7 [Source:MGI Symbol;Acc:MGI:1298407] | 1.655 | 0.726957439 | 0.000166703 | 0.011385945 | yes | up | 9.37 | 10.08 | 10.41 | 12.2 | 22.71 | 15.03 | 9.953333333 | 16.64666667 |
| ENSMUSG00000025747 | Tyms | thymidylate synthase [Source:MGI Symbol;Acc:MGI:98878] | 1.538 | 0.620840973 | 0.000482205 | 0.026563981 | yes | up | 12.29 | 15.65 | 13.62 | 24 | 24.64 | 17.68 | 13.85333333 | 22.10666667 |
| ENSMUSG00000047719 | Ubiad1 | UbiA prenyltransferase domain containing 1 [Source:MGI Symbol;Acc:MGI:1918957] | 0.559 | -0.840140999 | 3.36E-06 | 0.000479071 | yes | down | 11.72 | 11.47 | 14.14 | 6.5 | 6.99 | 7.22 | 12.44333333 | 6.903333333 |
| ENSMUSG00000089960 | Ugt1a1 | UDP glucuronosyltransferase 1 family, polypeptide A1 [Source:MGI Symbol;Acc:MGI:98898] | 16.251 | 4.022465954 | 7.76E-07 | 0.000137283 | yes | up | 0.91 | 1.7 | 1.49 | 3.63 | 39.36 | 26.09 | 1.366666667 | 23.02666667 |
| ENSMUSG00000090165 | Ugt1a10 | UDP glycosyltransferase 1 family, polypeptide A10 [Source:MGI Symbol;Acc:MGI:3580642] | 3.829 | 1.936828783 | 1.07E-09 | 4.34E-07 | yes | up | 19.28 | 12.49 | 11.11 | 36.62 | 94.44 | 39.97 | 14.29333333 | 57.01 |
| ENSMUSG00000090171 | Ugt1a2 | UDP glucuronosyltransferase 1 family, polypeptide A2 [Source:MGI Symbol;Acc:MGI:3576049] | 24.377 | 4.607466976 | 1.14E-06 | 0.000184383 | yes | up | 2.19 | 7.45 | 0.73 | 25.16 | 146.35 | 81.84 | 3.456666667 | 84.45 |
| ENSMUSG00000029260 | Ugt2b34 | UDP glucuronosyltransferase 2 family, polypeptide B34 [Source:MGI Symbol;Acc:MGI:2140962] | 2.185 | 1.127369336 | 3.19E-09 | 1.05E-06 | yes | up | 5.38 | 5.73 | 4.91 | 9.1 | 15.94 | 10.05 | 5.34 | 11.69666667 |
| ENSMUSG00000061906 | Ugt2b38 | UDP glucuronosyltransferase 2 family, polypeptide B38 [Source:MGI Symbol;Acc:MGI:2140794] | 0.421 | -1.246801187 | 6.79E-14 | 5.70E-11 | yes | down | 592.13 | 496.72 | 616.45 | 198.27 | 264.95 | 256.09 | 568.4333333 | 239.77 |
| ENSMUSG00000072664 | Ugt3a1 | UDP glycosyltransferases 3 family, polypeptide A1 [Source:MGI Symbol;Acc:MGI:2146055] | 0.537 | -0.896444893 | 7.85E-06 | 0.000984102 | yes | down | 418.66 | 438.46 | 442.85 | 269.23 | 185.16 | 227.65 | 433.3233333 | 227.3466667 |
| ENSMUSG00000049152 | Ugt3a2 | UDP glycosyltransferases 3 family, polypeptide A2 [Source:MGI Symbol;Acc:MGI:2145969] | 0.582 | -0.779868013 | 5.67E-05 | 0.004775412 | yes | down | 551.97 | 512.84 | 493.29 | 320.13 | 267.95 | 298.36 | 519.3666667 | 295.48 |
| ENSMUSG00000032854 | Ugt8a | UDP galactosyltransferase 8A [Source:MGI Symbol;Acc:MGI:109522] | 0.615 | -0.702081986 | 0.000226128 | 0.014229969 | yes | down | 22.38 | 24.32 | 22.91 | 10.5 | 13.25 | 17.98 | 23.20333333 | 13.91 |
| ENSMUSG00000001228 | Uhrf1 | ubiquitin-like, containing PHD and RING finger domains, 1 [Source:MGI Symbol;Acc:MGI:1338889] | 3.306 | 1.725002043 | 0.00017921 | 0.011996334 | yes | up | 0.16 | 0.19 | 0.21 | 0.39 | 1.01 | 0.59 | 0.186666667 | 0.663333333 |
| ENSMUSG00000022435 | Upk3a | uroplakin 3A [Source:MGI Symbol;Acc:MGI:98914] | 0.205 | -2.288528226 | 0.000160754 | 0.011069592 | yes | down | 5.7 | 9.73 | 1.12 | 1.06 | 0.68 | 1.49 | 5.516666667 | 1.076666667 |
| ENSMUSG00000024208 | Uqcc2 | ubiquinol-cytochrome c reductase complex assembly factor 2 [Source:MGI Symbol;Acc:MGI:1914517] | 1.687 | 0.754768025 | 0.000128426 | 0.0093412 | yes | up | 66.69 | 64.51 | 56.46 | 110.42 | 109.36 | 93.07 | 62.55333333 | 104.2833333 |
| ENSMUSG00000038775 | Vill | villin-like [Source:MGI Symbol;Acc:MGI:1201781] | 0.613 | -0.705169182 | 0.000220924 | 0.014113918 | yes | down | 15.58 | 18.41 | 19.9 | 12.99 | 11.69 | 12.1 | 17.96333333 | 12.26 |
| ENSMUSG00000042116 | Vwa1 | von Willebrand factor A domain containing 1 [Source:MGI Symbol;Acc:MGI:2179729] | 0.595 | -0.747831109 | 0.000710848 | 0.034619319 | yes | down | 6.46 | 5.31 | 8.16 | 3.28 | 4.24 | 4.34 | 6.643333333 | 3.953333333 |
| ENSMUSG00000070530 | Wfdc16 | WAP four-disulfide core domain 16 [Source:MGI Symbol;Acc:MGI:2670994] | 0.3 | -1.738140661 | 0.001092064 | 0.04741306 | yes | down | 1.55 | 4.42 | 2.87 | 0.5 | 1.67 | 0.47 | 2.946666667 | 0.88 |
| ENSMUSG00000017723 | Wfdc2 | WAP four-disulfide core domain 2 [Source:MGI Symbol;Acc:MGI:1914951] | 2.638 | 1.399478718 | 5.01E-08 | 1.17E-05 | yes | up | 72.41 | 78.05 | 65.57 | 105.42 | 291.99 | 187.53 | 72.01 | 194.98 |
| ENSMUSG00000015957 | Wnt11 | wingless-type MMTV integration site family, member 11 [Source:MGI Symbol;Acc:MGI:101948] | 0.123 | -3.02008224 | 8.27E-06 | 0.001021295 | yes | down | 1.04 | 1.03 | 1.75 | 0.07 | 0.06 | 0.16 | 1.273333333 | 0.096666667 |
| ENSMUSG00000018486 | Wnt9b | wingless-type MMTV integration site family, member 9B [Source:MGI Symbol;Acc:MGI:1197020] | 0.237 | -2.075142866 | 0.000850507 | 0.039585108 | yes | down | 0.55 | 0.52 | 0.21 | 0.03 | 0.11 | 0.17 | 0.426666667 | 0.103333333 |
| ENSMUSG00000066829 | Zfp810 | zinc finger protein 810 [Source:MGI Symbol;Acc:MGI:2384563] | 0.605 | -0.725644567 | 0.000158926 | 0.0110342 | yes | down | 9.3 | 8.3 | 7.19 | 4.03 | 6.78 | 5.42 | 8.263333333 | 5.41 |
| ENSMUSG00000056300 | Zfp981 | zinc finger protein 981 [Source:MGI Symbol;Acc:MGI:3700965] | 0.169 | -2.5636843 | 1.10E-08 | 2.99E-06 | yes | down | 3.14 | 5.21 | 1.72 | 0.24 | 0.82 | 0.67 | 3.356666667 | 0.576666667 |
