# Supplementary material for: BDH1-mediated βOHB metabolism ameliorates diabetic kidney disease by activation of NRF2-mediated antioxidative pathway
Source: Aging (Albany NY). 2023 Nov 27;15(22):13384–410. doi: 10.18632/aging.205248 (PMC11622694; doi:10.18632/aging.205248)
Supplement: Supplementary Tables 2-4 [file aging-15-205248-s004.pdf]

SUPPLEMENTARY TABLES

Supplementary Table 2. Characteristics of control subjects and patients with diabetic kidney disease (DKD).

	Control (*n* = 9)	DKD (*n* = 8)
Demographics		
Age (years)	54 ± 3.94	56.63 ± 5.41
Sex (male/female)	5/4	5/3
BMI (kg/m^2)	21.47 ± 1.71	24.86 ± 2.42
Diabetes duration (years)	N/A	14.63 ± 5.85
DKD duration (years)	N/A	5.21 ± 2.17
Retinopathy (yes/no)	0/9	5/3
SBP (mmHg)	119.78 ± 3.80	141.13 ± 10.18
DBP (mmHg)	75.33 ± 4.16	87.25 ± 14.78
Laboratory data		
UAE (mg/24 h)	N/A	3293.66 (1000–6530)
eGFR (CKD-EPI) (ml/min/1.73 m^2)	132.81 ± 7.00	72.01 ± 31.76
HbA1c (%)	N/A	8.15 ± 1.12
Serum cholesterol (mmol/L)	4.51 ± 0.86	4.73 ± 2.21
Serum triglyceride (mmol/L)	1.25 ± 0.18	3.22 ± 3.06
HDL-C (mmol/L)	1.55 ± 0.22	1.09 ± 0.45
LDL-C (mmol/L)	2.78 ± 0.45	2.58 ± 2.03
Medications		
Insulin (yes/no)	0/9	5/3
ACEI or ARB (yes/no)	0/9	3/5

Abbreviations: ACEI: angiotensin-converting enzyme inhibitor; ARB: angiotensin II receptor blocker; BMI: body mass index; DBP: diastolic blood pressure; eGFR: estimated glomerular filtration rate; HbA1c: hemoglobin A1C; N/A: not available; SBP: systolic blood pressure; UAE: urinary albumin excretion; HDL-C: high density lipoprotein cholesterol; LDL-C: low-density lipoprotein cholesterol.

Supplementary Table 3. Body weight, kidney weight and kidney to body weight ratios in each group (x̄ ± standard deviation).

Characteristics	m/m (*n* = 5)	db/db + AAV9-Vector-GFP (*n* = 4)	db/db + AAV9-BDH1-GFP (*n* = 4)	db/db + SD (*n* = 4)	db/db + KD (*n* = 4)	m/m (*n* = 5)	db/db + Vehicle (*n* = 5)	db/db + βOHB (*n* = 5)
Body weight (g)	27.02 ± 2.28	49.65 ± 2.77	52.6 ± 5.43	48.73 ± 1.47	57.18 ± 5.35	23.74 ± 1.38	63.68 ± 5.37	60.16 ± 4.52
Kidney weight (g)	0.18 ± 0.01	0.23 ± 0.02	0.24 ± 0.03	0.2 ± 0.01	0.14 ± 0.01	0.17 ± 0.02	0.25 ± 0.03	0.23 ± 0.04
Kidney weight/ Body weight (mg/g)	6.64 ± 0.83	4.62 ± 0.54	4.5 ± 0.28	4.12 ± 0.29	2.52 ± 0.41**	6.94 ± 0.79	4.04 ± 0.68	3.87 ± 0.54

**$p < 0.01$.

Supplementary Table 4. List of primers used in this study.

Species	NCBI Gene ID	Gene name	Sequence (5′ to 3′)	Length of product (bp)
Mus	71911	*Bdh1*	F: CGGCTAGTGGCAAAGCTATC<br>R: GTTGCAGACATTGAGCTGGA	194
	67041	*Oxct1*	F: CATAAGGGGTGTGTCTGCTACT<br>R: GCAAGGTTGCACCATTAGGAAT	106
	110446	*Acat1*	F: CAGGAAGTAAGATGCCTGGAAC<br>R: TTCACCCCCTTGGATGACATT	228
	208715	*Hmgcs1*	F: AACTGGTGCAGAAATCTCTAGC<br>R: GGTTGAATAGCTCAGAACTAGCC	180
	23942	*Mta2*	F: TGTACCGGGTGGGAGATTAC<br>R: CCTTCGCCGGAAAAGACAG	134
	14630	*Gclm*	F: AGGAGCTTCGGGACTGTATCC<br>R: GGGACATGGTGCATTCCAAAA	105
	625249	*Gpx4*	F: GATGGAGCCCATTCCTGAACC<br>R: CCCTGTACTTATCCAGGCAGA	185
	20656	*Sod2*	F: CAGACCTGCCTTACGACTATGG<br>R: CTCGGTGGCGTTGAGATTGTT	113
	11461	*β-Actin*	F: ACCTCTATGCCAACACAGTG<br>R: GGACTCATCGTACTCCTGCT	221
Homo	622	*BDH1*	F: GACAGCCTAAACAGTGACCGA<br>R: GAGCGGACAATCTCCACCA	89
	9219	*MTA2*	F: GCGCAGGGACATTTCTAGTAG<br>R: TGGGTGGCTGGTAATGATTCA	162
	2730	*GCLM*	F: TGTCTTGGAATGCACTGTATCTC<br>R: CCCAGTAAGGCTGTAAATGCTC	239
	2879	*GPX4*	F: GAGGCAAGACCGAAGTAAACTAC<br>R: CCGAACTGGTTACACGGGAA	100
	6648	*SOD2*	F: GCTCCGGTTTTGGGGTATCTG<br>R: GCGTTGATGTGAGGTTCCAG	92
	3553	*IL-1β*	F: ATGATGGCTTATTACAGTGGCAA<br>R: GTCGGAGATTCGTAGCTGGA	132
	3606	*IL-18*	F: TCTTCATTGACCAAGGAAATCGG<br>R: TCCGGGGTGCATTATCTCTAC	75
	60	*β-ACTIN*	F: CATGTACGTTGCTATCCAGGC<br>R: CTCCTTAATGTCACGCACGAT	250
